# Haploinsufficiency underlies the neurodevelopmental consequences of *SLC6A1* variants

**DOI:** 10.1016/j.ajhg.2024.04.021

**Published:** 2024-05-22

**Authors:** Dina Buitrago Silva, Marena Trinidad, Alicia Ljungdahl, Jezrael L. Revalde, Geoffrey Y. Berguig, William Wallace, Cory S. Patrick, Lorenzo Bomba, Michelle Arkin, Shan Dong, Karol Estrada, Keino Hutchinson, Jonathan H. LeBowitz, Avner Schlessinger, Katrine M. Johannesen, Rikke S. Møller, Kathleen M. Giacomini, Steven Froelich, Stephan J. Sanders, Arthur Wuster

**Affiliations:** 1 Department of Bioengineering and Therapeutic Sciences, University of California, San Francisco, San Francisco, CA, USA; 2 BioMarin Pharmaceutical Inc., Novato, CA, USA; 3 Department of Molecular and Cell Biology, University of California, Berkeley, Berkeley, CA, USA; 4 Innovative Genomics Institute, University of California, Berkeley, Berkeley, CA, USA; 5 Department of Psychiatry, UCSF Weill Institute for Neurosciences, University of California, San Francisco, San Francisco, CA, USA; 6 Institute of Developmental and Regenerative Medicine, Department of Paediatrics, University of Oxford, Oxford OX3 7TY, UK; 7 Department of Pharmaceutical Chemistry, University of California, San Francisco, San Francisco, CA, USA; 8 Department of Pharmacological Sciences, Icahn School of Medicine at Mount Sinai, New York, NY 10029, USA; 9 Department of Regional Health Research, Faculty of Health Sciences, University of Southern Denmark, Odense, Denmark; 10 Department of Epilepsy Genetics and Personalized Medicine, Member of ERN Epicare, Danish Epilepsy Centre, Dianalund, Denmark

**Keywords:** SLC6A1, GAT-1, GAT1, epilepsy with myoclonic-atonic seizures, autism spectrum disorders, neurodevelopmental delay, GABA uptake, missense vulnerability

## Abstract

Heterozygous variants in *SLC6A1*, encoding the GAT-1 GABA transporter, are associated with seizures, developmental delay, and autism. The majority of affected individuals carry missense variants, many of which are recurrent germline *de novo* mutations, raising the possibility of gain-of-function or dominant-negative effects. To understand the functional consequences, we performed an *in vitro* GABA uptake assay for 213 unique variants, including 24 control variants. *De novo* variants consistently resulted in a decrease in GABA uptake, in keeping with haploinsufficiency underlying all neurodevelopmental phenotypes. Where present, ClinVar pathogenicity reports correlated well with GABA uptake data; the functional data can inform future reports for the remaining 72% of unscored variants. Surface localization was assessed for 86 variants; two-thirds of loss-of-function missense variants prevented GAT-1 from being present on the membrane while GAT-1 was on the surface but with reduced activity for the remaining third. Surprisingly, recurrent *de novo* missense variants showed moderate loss-of-function effects that reduced GABA uptake with no evidence for dominant-negative or gain-of-function effects. Using linear regression across multiple missense severity scores to extrapolate the functional data to all potential *SLC6A1* missense variants, we observe an abundance of GAT-1 residues that are sensitive to substitution. The extent of this missense vulnerability accounts for the clinically observed missense enrichment; overlap with hypermutable CpG sites accounts for the recurrent missense variants. Strategies to increase the expression of the wild-type *SLC6A1* allele are likely to be beneficial across neurodevelopmental disorders, though the developmental stage and extent of required rescue remain unknown.

## Graphical abstract

**Figure:**
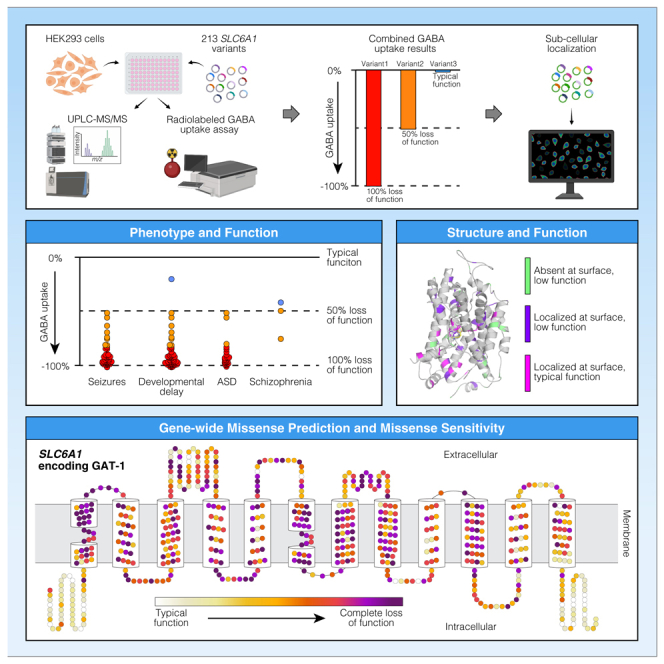

## Teaser

Germline *de novo* variants in *SLC6A1* are a major cause of neurodevelopmental disorders, including seizures. Enrichment for missense variants, many of which are recurrent, suggests a gain-of-function mechanism. However, here, we show that such variants consistently decreased GABA uptake *in vitro*, supporting a haploinsufficiency mechanism underlying the observed neurodevelopmental phenotypes.

## Introduction

Large-scale exome sequencing studies have identified the gene *SLC6A1* (solute carrier family 6 member 1, ENSG00000157103), which encodes the GABA transporter protein “GAT-1,” as a major cause of neurodevelopmental disorders (NDD). Genome-wide significant association has been reported for rare heterozygous variants in independent cohorts of developmental delay (DD),^1^ autism spectrum disorder (ASD),^2^ and pediatric-onset epilepsy, particularly epilepsy with myoclonic-atonic seizures (EMAS, previously myoclonic-atonic seizures or MAE)^3^; it has also been implicated in schizophrenia.^4^^,^^5^ Across these disorders, the incidence related to *SLC6A1* variants is estimated to be 2.4–2.9 in 100,000 births,^6^ making it a relatively common single-gene disorder. These features make *SLC6A1*, encoding GAT-1, a promising target for novel therapeutics.

Realizing the therapeutic potential of *SLC6A1* requires understanding the functional impact of the genetic variants observed in different phenotypes. GAT-1 is predominantly found embedded in the cell surface membrane and transports the inhibitory neurotransmitter GABA from the synaptic cleft into presynaptic neurons,^7^ which may limit the inhibition of postsynaptic neurons and prepare the presynaptic neuron for further GABA release. *SLC6A1* is very highly expressed in *SV2C*/*LAMP5*-expressing GABAergic (inhibitory) neurons, highly expressed in other classes of GABAergic neurons (expressing *PVALB*, *VIP*, or *SST*), and weakly expressed in multiple non-neuronal cell types, including astrocytes, oligodendrocytes, oligodendrocyte precursor cells, and endothelial cells^8^; there is minimal expression in excitatory glutamatergic neurons. It is widely expressed across brain regions, with expression increasing rapidly during mid to late fetal development, especially in the striatum, before reaching a steady state from birth to late adulthood.^9^

The majority of *SLC6A1* variants associated with human disorders are predicted to be missense variants or in-frame indels, some of which are recurrent *de novo* mutations (e.g., 11 individuals with c.863C>T [p.Ala288Val] and 10 individuals with c.1024G>A [p.Val342Met], Figure S1). This distribution is highly suggestive of a gain-of-function or dominant-negative mechanism, as has been observed in numerous other rare genetic disorders across many phenotypes,^1^^,^^2^^,^^3^ and has motivated a clinical trial to inhibit GAT-1 in schizophrenia.^4^ Conversely, there are also multiple individuals with protein-truncating variants (PTVs, including stop gain, frameshift, and canonical splice site variants), suggesting a co-existing loss-of-function mechanism. The function of 29 *SLC6A1* variants has previously been characterized using GABA uptake assays, including four PTVs resulting in complete loss of GABA uptake and 25 missense/in-frame variants with varying degrees of loss-of-function^10^^,^^11^^,^^12^^,^^13^^,^^14^ (Table S2). Given the loss-of-function outcomes observed, it remains unclear why *SLC6A1* is strongly enriched for missense/in-frame variants rather than the PTVs with clear loss-of-function mechanisms that are observed in most genes associated with neurodevelopmental disorders.^2^ Possibilities include undiscovered gain-of-function variants, genotype-phenotype relationships that vary by disorder, as observed in *SCN2A*,^15^ prenatal lethality, or a dominant-negative effect, as observed in *SLC30A2*.^16^

Here, we present data on the functional impact on GABA uptake of 213 *SLC6A1* variants, 185 of which have not previously been characterized,^10^^,^^11^^,^^12^^,^^13^^,^^14^ including variants associated with schizophrenia, the majority of recurrent variants, and “control” variants that are synonymous, common, or documented as benign in ClinVar. We assess the surface localization of 86 of these variants and explore the results in the context of the previously published GAT-1 structure^17^ with updated annotations of transmembrane (TM) domains, and intra- and extra-cellular loops. In addition, we explore the possibility of gain-of-function effects in stable lines for ten variants and dominant-negative effects for seven variants. Finally, through integrative analysis of genomic data, we demonstrate that GAT-1 is “delicate” so that multiple missense variants can lead to clinically relevant haploinsufficiency. This leads to a parsimonious model of *SLC6A1*-related disorders via a heterozygous loss-of-function mechanism with the observed missense enrichment explained by GAT-1 missense sensitivity and the recurrent missense variants explained by hypermutable CpG loci.

## Material and methods

Variant selection and functional assays were performed independently by teams at BioMarin and UCSF before integrating the data for a combined analysis of 213 unique variants. Combined methods are described below, with distinctions highlighted between groups as required. For detailed methods from each research group please see supplemental material and methods at the end of this manuscript.

### Variant selection

Individuals with variants in *SLC6A1* were identified from multiple sources, including ClinVar,^18^ gnomAD,^19^ multiple cohort studies,^1^^,^^2^^,^^3^^,^^4^ case series and reports,^20^ and three previously undescribed individuals from the Møller group in Denmark. Across these sources, 400 unique individuals were identified in total (Table S1), mapping to 323 unique variants in individuals plus a further ten common missense variants from gnomAD (Table S2). Variants were selected for functional interpretation based on recurrence across multiple individuals, variants in individuals with detailed phenotyping data, distribution across phenotypes, distribution across GAT-1, distribution of predicted effect on GAT-1, and distribution of population frequency. The BioMarin group assayed 181 variants, the UCSF group assayed 100 variants, and 68 of these variants were assayed by both groups (Table S2). Twenty-four variants were selected as controls, composed of six synonymous variants, ten missense variants observed in multiple individuals in gnomAD population controls,^19^ and seven missense variants predicted to be benign from clinical sequencing as reported in ClinVar.^18^ The full list of individuals and phenotypes is reported in Table S1, and the full list of variants, genomic annotations, and functional results are reported in Table S2.

### Annotation of *SLC6A1* variants

Variant impact was estimated using the ENST00000287766.10 transcript (RefSeq: NM_003042.4) for the ENSG00000157103.12 *SLC6A1* as defined by GENCODE v39. All reported *SLC6A1* variants in ClinVar were downloaded on June 22, 2022 to annotate clinical significance. All *SLC6A1* variants in the “non-neuro” version of gnomADv2.1.1 were downloaded on August 18, 2022 to annotate gnomAD population allele frequency. gnomAD variant frequency estimates across all ancestries were used. Predicted missense severity was annotated using ANNOVar protocol “dbnsfp42a” and build “hg38.”^21^

### Gene constructs and GFP tagging

The *SLC6A1* consensus coding sequence (CCDS: CCDS2603.1, NM_003042) was used for the creation of both gene constructs from each group under the cytomegalovirus promoter, but with different vectors. The resulting wild-type (WT) *SLC6A1* constructs from each group were sent to Genscript (Piscataway, NJ) to perform site-specific mutagenesis to generate 181 distinct variants (BioMarin team) and 100 distinct variants (UCSF group). Genscript internally confirmed the quality and sequence of all plasmids, and the expected variant was further confirmed by Sanger sequencing.

For the GFP-tagged plasmids for 86 missense variants, the 249 amino acid monomeric superfolder green fluorescent protein (sfGFP, ASSN: ASL68970) was first synthesized by Genscript. Separately, the UCSF research group sent the WT-SLC6A1 construct back to Genscript, and the sfGFP was cloned into the C-terminal end of the *SLC6A1* transcript with no spacer or linker. The final plasmid, WT-SLC6A1-sfGFP, was verified by Genscript and tested in the [^3^H]-GABA transport assay to compare relative uptake to WT-SLC6A1. No difference in GABA uptake was observed. To create the 86 GFP-tagged variant plasmids, Genscript performed site-directed mutagenesis on the WT-SLC6A1-sfGFP plasmid and verified every individual variant by Sanger sequencing.

### Preparation of cell lines and transfection of variants

Both research groups prepared human embryonic kidney cells (HEK293) separately to study the functional effects of *SLC6A1* variants. The BioMarin team employed CRISPR/Cas9 to generate a *SLC6A1*-deficient HEK293T cell line (see supplemental material and methods). The GABA uptake assay was used to confirm loss of GAT-1 activity in the knockout line. The UCSF research group used the HEK293 Flp-In integration system and an empty vector (EV) control in every transfection experiment to control for any background GABA uptake in the cells (see supplemental material and methods). The [^3^H]-GABA transport assay was used before experimental design to compare background activity (EV) to WT activity (WT-*SLC6A1*). Plasmid sequences are reported in Table S3.

### GABA uptake assay and [^3^H]-GABA transport assay

The BioMarin research team employed ultra-high performance liquid chromatography-MS/MS (UPLC-MS/MS) in their experimental design to functionally characterize controls and variants. Beta-lactamase (BLA) activity was measured to account for transfection variability. The UCSF research group used a radiolabeled uptake assay to characterize GABA transport of variants and controls. Both groups used the Pierce BCA Protein Assay (Thermo Fisher Scientific, Cat. 23227) to measure total protein in each well, accounting for plating variability. Refer to the supplemental material and methods for further detailing of each assay.

### Combining uptake data across cohorts

For each cohort (BioMarin and UCSF) the data from the three to six replicates (Table S4) that passed quality control (count) was used to estimate the mean. Log-transformed mean estimates were compared between the two groups. Based on the strong correlation values observed between the functional data generated by BioMarin and UCSF (Figure S2), we calculated the mean of GABA uptake from the 68 variants where both groups had estimated GABA uptake. We made one exception, the insertion chr3:11020264:T:TA (hg38), c.523_524insA (p.Ser175Tyrfs^∗^32), which is predicted to induce a frameshift variant and premature stop codon in GAT-1. The UCSF data predicted no GABA uptake (−102.4%) while the BioMarin data predicted reduced update (−73.2%). Given that 22 other PTVs had yielded a GABA uptake value below −92.3%, we elected to choose the UCSF value of −102.4% for the combined result.

### Defining thresholds for functional impact

The GABA uptake values for 24 control variants (Table S2) were normally distributed around a mean of −0.8% with a standard deviation of 24.7%. We used *Z* score thresholds of 1.96 standard deviations (48.4%, *p* ≤ 0.05, two-sided) to define loss-of-function (−0.8% – 48.4% = −49.2%) and gain-of-function (−0.8% + 48.4% = 47.5%) and 3.29 standard deviations (81.2%, *p* ≤ 0.001, two-sided) to define severe loss-of-function (−0.8% – 81.2% = −82.1%). No variants were above 80.4%, which would be the equivalent severe gain-of-function threshold.

### Immunostaining

For immunostaining experiments, HEK293 cells were plated on poly-D-lysine-treated 96-well black PhenoPlate (PerkinElmer Health Sciences) with an optically clear flat-bottom and at an optimized cell density of 2.4 × 10^4^ cells/well. All wells on the edges of the plate were excluded to prevent edge effects. Cells were reverse transfected with respective SLC6A1-sfGFP-mutant plasmid and controls (WT-SLC6A1-sfGFP and EV-sfGFP). Mutant plasmids were transfected in 3 biological replicates and performed in duplicate. WT and EV were treated the same but had an additional plate with 12 biological replicates per plasmid and performed in duplicate. After 48 h, cells were washed once with Hank’s Balanced Salt Solution (HBSS), and then the plasma membrane was stained with Wheat Germ Agglutin Alexa Fluor 647 conjugate (Invitrogen Life Sciences) diluted (1:1000) in cold HBSS for 5 min at room temperature. The stain was then aspirated, and cells were carefully washed three times with HBSS. Cells were fixed with 3.7% formaldehyde in HBSS for 15 min. The fixing solution was then aspirated, and cells were washed again three times with HBSS. Next, the cytoplasm was stained using HCS CellMask Orange Stain (Invitrogen Life Sciences) in HBSS (1:1000) for 5 min at room temperature and kept in darkness. The stain was aspirated, and cells were washed two more times with HBSS. Lastly, the nucleus was stained with Hoescht in HBSS (1:2000) for 20 min at room temperature and kept in darkness. Cells were washed twice with HBSS, and 100 μL of buffer was left in the wells for imaging. Imaging was performed within 24 h of staining or the same day and then stored in a 4°C fridge wrapped in aluminum foil.

### High-throughput confocal imaging with IN Cell 6500HS Analyzer and IN Carta Software analysis

Immunostained plates were imaged with the IN Cell Analyzer 6500HS (GE Healthcare, WA, USA). All images were taken with a Nikon 40× objective with the laser and software autofocus (AF) applied. A total of eight fields of view were captured per well across all plates (*n* = 48 total per plasmid: eight fields of view, three biological replicates, performed twice). Four channels were used to capture the respective stain or fluorescence. The blue laser (405 nM excitation and 455/50 nM emission) captured the Hoechst stain. The orange laser (561 nM excitation and 605/51 nM emission) captured the orange HCS CellMask stain. The far-red laser (642 nM excitation and 682/59 nM emission) captured the Alexa Fluor 647 conjugate. The green laser (488 nM excitation and 524/48 nM emission) captured GFP fluorescence.

The resulting images were visualized and analyzed with the IN Carta image analysis software (Molecular Devices, San Jose, CA). The protocol segmented the images by cell components. The blue wavelength was used to segment the mono-nucleated cells. The orange wavelength was used to segment the whole cell using the orange HCS CellMask. Once the software identified the nucleus and whole cell, the membrane was segmented using the red wavelength. For example images, see Figure S3. The protocol was run, and Pearson correlation values were generated for GFP fluorescence colocalization with the plasma membrane stain (Alexa Fluor 647). Pearson correlation values are calculated through the software using total intensities (total intensity = intensity × cell area) of the respective wavelengths captured in the field of view(s). After all values were generated from this protocol, the classifier tool within the IN Carta software was applied to further refine Pearson correlation raw scores. This tool allows the user to define upper and lower bounds for intensities detected across the entire plate, helping to account for plate-to-plate variability. Lower bounds were classified as “low GFP” fluorescence or background that is detected by the software. Upper bounds were classified as an “overexposed GFP” signal, which is bright puncta detected. Between the upper and lower bounds, there was a “high GFP” signal, and the classifier tool reran the protocol with these newly defined classes. Newly generated values consider Pearson correlation scores by class. All final analyses in this study were conducted based on the “high GFP” class (Figure S4).

### Protein structure mapping in 2D and 3D (combined datasets)

To visualize the topology of GAT-1 and percent-wild-type uptake activity of missense variants, the UCSF TM protein display tool TOPO2 was used (https://www.cgl.ucsf.edu/Overview/ftp/topo2.tar.gz). The topological domains of *SLC6A1* were annotated in two different ways (Tables S1 and S2). The initial annotations were taken from UniProt (ID P30532). The second annotations, used for our primary analysis and all topology figures and 3D representations, used the recently published 3D structure of GAT-1.^17^ Molecular docking was performed using Glide^22^ from the Schrödinger suite. GABA was docked to the cryo-EM structure of GAT-1 (PDB: 7SK2). The ligand binding site was defined based on the coordinates of Tiagabine in the published structure. The docking results, figures, and videos were visualized via PyMOL, version 2.5.

### Data analysis and figures

Data were analyzed using Python (including pandas, NumPy, statsmodels, matplotlib, and seaborn libraries). Figures were composed using Adobe Illustrator. Further details on individual analysis can be found under supplemental material and methods.

## Results

### Impact of 213 variants in *SLC6A1* on GABA uptake activity

A combined search of publications and large-scale genomic cohorts identified 400 individuals with variants in *SLC6A1*, which mapped to 323 unique variants^4^^,^^10^^,^^11^^,^^19^^,^^20^^,^^23^^,^^24^^,^^25^^,^^26^^,^^27^^,^^28^^,^^29^^,^^30^ (Table S1). Our two groups (BioMarin, UCSF) independently selected subsets of these variants for functional analysis of GABA uptake using a [^3^H]-GABA transport assay in HEK293 cells (supplemental material and methods). Assays of GABA uptake are reported as the percent difference from WT, with 0% being the same as WT and −100% being the complete absence of GABA uptake. In total, 213 variants in *SLC6A1* were assessed, and the 68 variants analyzed by both groups yielded consistent results (R^2^ = 0.79, *p* = 5.3 × 10^−24^, Figure S2). Our data were also consistent with previously published results (*n* = 21; R^2^ = 0.51,^12^ *n* = 7; R^2^ = 0.67,^11^ Figure S2).

**Figure 1:**
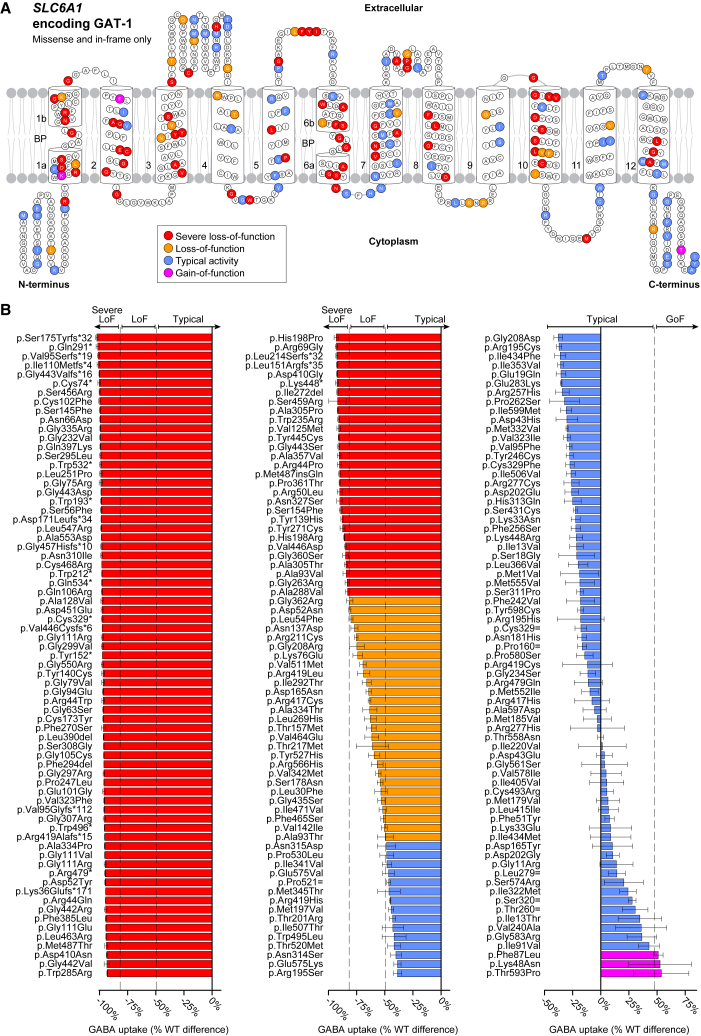
Topology of GAT-1 and GABA uptake values by functional type (A) 2D representation of GAT-1 organized by 12 TM domains and linking or terminal chains. Only missense and in-frame variants are highlighted. (B) GABA uptake functional data as a percentage of WT and highlighted by activity type for 213 variants tested across the 599 amino acids of GAT-1. Individual data bars represent mean ± SEM of three biological replicates performed in triplicates. GoF, gain-of-function; LoF, loss-of-function; WT, wildtype.

The 213 variants included 24 control variants predicted not to contribute substantial risk for neurodevelopmental disorders: six synonymous variants, ten missense variants selected because they were observed in gnomAD population controls,^19^ and seven missense variants predicted to be benign from clinical sequencing reported in ClinVar.^18^ Across these 24 control variants, GABA uptake was equivalent to the reference (non-variant) version of *SLC6A1* (mean = −0.8%, SD = 24.7%, range = −46.6%–51.0%). We used these variants to define thresholds based on Z scores for severe loss-of-function (≤−82.1%, *p* ≤ 0.001, 100 variants), loss-of-function (>−82.1% and ≤−49.2%, *p* ≤ 0.05, 25 variants), and gain-of-function (>47.5%, *p* ≤ 0.05, 3 variants), with 85 variants in the typical range (>−49.2% to ≤47.5%) (Figure 1B). These four variant effect types are highlighted on a 2D-topology representation of GAT-1 for missense variants (Figure 1A). To see 2D topology of other previously published groups,^11^^,^^12^ refer to Figure S5.

### Limited evidence for gain-of-function variants

Three variants exceeded our gain-of-function threshold of >47.5% (Figure 1). One of these, c.259T>C (p.Phe87Leu), was a control variant selected from population controls in gnomAD. Another, c.144G>T (p.Lys48Asn), was a variant of unknown significance and unknown inheritance that is also present in gnomAD that was identified in an individual with early-onset epileptic encephalopathy.^30^ The third one, c.1777A>C (p.Thr593Pro), was a variant of unknown significance absent from gnomAD that was reported in ClinVar without clinical details. Given the caveats of upregulating GAT-1 in HEK293 cells for uptake assays, we sought to assess whether these represented genuine gain-of-function effects. Stable cell lines were generated for the ten variants with the highest GABA uptake values in the UCSF cohort, including p.Phe87Leu and p.Thr593Pro but not p.Lys48Asn. These ten stable cell lines were retested for GABA uptake activity (Figure S6). Across all ten, the uptake values were substantially lower than those observed in the transiently transfected cell lines and exhibited a non-significant trend to being above the WT levels (i.e., 100%). If variants do increase GABA uptake, the effects are small and unlikely to contribute substantially to neurodevelopmental phenotypes.

### Neurodevelopmental phenotypes are consistently associated with loss-of-function effects

**Figure 2:**
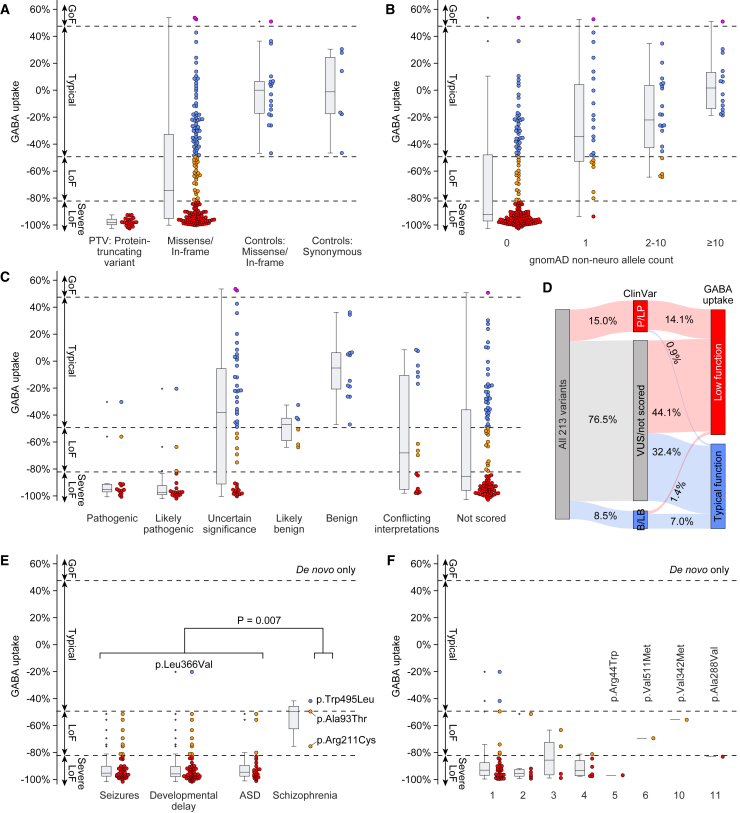
GABA uptake of variants by phenotype and recurrence (A) GABA uptake data for 213 variants by predicted impact on GAT-1. PTVs and missense/in-frame variants are ascertained from clinical populations (left two categories) while 24 variants selected as controls were split between missense/in-frame and synonymous variants (right two categories) and used to set thresholds for loss of function and gain of function (dashed lines). (B and C) GABA uptake values by population allele count in 114,704 non-neuropsychiatric samples in gnomAD and by ClinVar clinical significance. (D) A Sankey plot summarizing the proportion of variants where ClinVar category could be reclassified. (E) GABA uptake values for *de novo* variants by the presence of seizures, DD, autism spectrum disorder (ASD), or schizophrenia. If phenotypes are comorbid (e.g., seizures and DD) the variant is shown for all phenotypes for which it is reported. All variants are shown in Figure S8B. (F) GABA uptake values of *de novo* variants observed in single individuals (1) or multiple individuals (2–11). Each variant is shown once. The four most recurrent variants (5, 6, 10, and 11) are labeled. Abbreviations: GoF, gain-of-function; LoF, loss-of-function; A93T, p.Ala93Thr; R211C, p.Arg211Cys; W495L, p.Trp495Leu. Statistical tests: E, two-sided Wilcoxon test. Colors: red, severe LoF; orange, LoF; blue, typical activity; pink, GoF. Boxplot whiskers are based on maximum and mimimum values within 1.5 times the interquartile range.

All 23 PTVs assessed resulted in almost no GABA uptake (i.e., severe loss-of-function), consistent with nonsense-mediated decay or non-functional GAT-1 (Figure 2A). Of 166 clinically ascertained missense variants/in-frame indels, 77 were severe loss-of-function, 27 were loss-of-function, two were gain-of-function, and 60 were in the typical range. Even missense variants/in-frame indels in the typical range had a median GABA uptake of −25.7%, suggesting that a subset of these may also contribute some neurodevelopmental risk via mild loss-of-function (Figure 2A). Across all variants assayed, GABA uptake was correlated with population allele frequency (R^2^ = 0.37, *p* = 8.4 × 10^−24^, Figure S7). Of the 156 variants absent in 114,704 population controls, 115 (73.7%) resulted in severe loss-of-function or loss-of-function. In contrast, none of the variants observed in ten or more individuals showed evidence of reduced GABA uptake (Figure 2B).

Clinical significance has previously been stated for 113 variants (Tables S2 and S5). Where clear determinations were made, these were generally supported by the functional assay: 32 (5 PTVs, 27 missense) were reported as pathogenic or likely pathogenic, and 30 (93.8%) of these were indeed found to be loss-of-function, while 18 missense variants were reported as benign or likely benign of which 15 (83.3%) were in the typical range (Figure 2C). The remaining 163 variants had uncertain (*n* = 46), conflicting (*n* = 16), or absent (*n* = 101) clinical significance; 94 (57.7%) of these resulted in severe loss-of-function or loss-of-function. Thus, our functional data are consistent with 97 (45.5%) of the remaining variants being clinically relevant and 71 (33.3%) variants having minimal impact on function (Figure 2D).

To minimize the ascertainment bias, we assessed genotype-phenotype correlations focusing on the 67 *de novo* variants (Figures 2E and S8); all variants are shown in Figure S8B. All but one of these variants were identified in an individual diagnosed with a neurodevelopmental disorder, the exception being c.752T>C (p.Leu251Pro), reported in an individual with no seizures at 21 months of age and no data reported regarding ASD or DD.^20^ The completeness of the phenotyping data varied by variant (Tables S1 and S2). Seizures were reported in 46 of the 55 (83.6%) with data, ASD reported in 24 of 44 (54.5%), and DD in 58 of 59 (98.3%). Variants were consistently loss-of-function in individuals with seizures, ASD, and DD (median GABA uptake −0.95, −0.95, and −0.96, respectively). The most frequently reported seizure type was EMAS, reported in 22 of the 37 (59.5%) for whom seizure type was available. In addition, early-onset absence epilepsy (EOAE), childhood absence epilepsy (CAE), developmental and epileptic encephalopathy (DEE), and Lennox-Gastaut syndrome (LGS) were related to reduced GABA uptake. In contrast, non-familial, non-acquired focal epilepsy (NAFE) consistently resulted in typical levels of GABA uptake (Figure S8), suggesting *SLC6A1* disruption is probably not associated with NAFE. No clear patterns were observed between variant effect and age of seizure onset (Figure S9).

### Some variants observed in schizophrenia have loss-of-function effects

Two schizophrenia cohorts contributed *SLC6A1* missense variants that were predicted to be damaging (MPC score ≥2).^31^ The first focused on *de novo* variants from 3,444 individuals and reported that *SLC6A1* was associated with schizophrenia (*p* = 7.9 × 10^−5^, uncorrected).^4^ Two of the three *de novo* variants resulted in loss-of-function effects (c.277G>A [p.Ala93Thr] and c.631C>T [p.Arg211Cys]), while one was in the typical range (c.1484G>T [p.Trp495Leu]). The median GABA uptake across these variants is −49.5%—a value in the loss-of-function range but higher than that observed for the neurodevelopmental phenotypes (*p* = 0.007, two-sided Wilcoxon test; Figure 2E). The second cohort was a case-control analysis of 24,248 individuals (including the previous 3,444); they reported enrichment for *SLC6A1* variants that did not meet genome-wide correction (*p* = 0.006, uncorrected; *p* = 0.50, corrected).^5^ While these additional variants were not reported to be *de novo* in the individuals with schizophrenia, two of the missense variants from this cohort were at the same location as *de novo* variants identified in neurodevelopmental disorders: c.913G>A (p.Ala305Thr) (severe loss-of-function) and c.1000G>A (p.Ala334Thr) (loss-of-function). Of the remaining 11 variants in the second cohort, two resulted in severe loss-of-function (c.1228G>A [p.Asp410Asn] and c.1324G>A [p.Gly442Arg]), two in loss-of-function (c.409A>G [p.Asn137Asp] and c.1249C>T [p.Arg417Cys]), and seven were in the typical range (Table S2; Figure S8).

### Correlation of functional data and surface localization of 86 missense variants

Given the role of loss-of-function effects for the clinically relevant *SLC6A1* missense variants, we sought to understand the relationship of protein trafficking mechanisms to these functional outcomes. A reduction in GAT-1 stability could lead to a relative deficit in surface colocalization.^10^^,^^12^ Surface localization was assessed for 86 missense variants using a high-content imaging approach to assess the relative co-localization of GFP-tagged GAT-1 with a membrane stain. While WT GAT-1 is located on the plasma membrane, it is also distributed at lower levels throughout the cell body in HEK293 cells (Figure S4). Given this mixed localization, the high-content imaging experimental design utilized segmentation as part of the image analysis to quantify the colocalization of GFP tag and plasma membrane stain through the Pearson correlation colocalization (PCC) metric. The WT GAT-1 GFP-tagged plasmid yielded a PCC value of 0.32 (*n* = 26), which we defined as 100% WT surface localization. As a negative control, we used an EV plasmid that contained GFP but not GAT-1; the resulting GFP is distributed non-specifically throughout the cell, with only a small amount on the membrane (Figure S4). The EV plasmid resulted in a PCC value of 0.15 (*n* = 26 replicates), 46.9% below the WT GAT-1 value (Figure S10). For each of the 86 missense variants, we tested six replicates (mean of 5.9 replicates after data cleaning) from which we calculated both the PCC and the percentage WT surface localization (Figure S10). For further analysis, the percentage WT surface localization values below that of EV (46.9%) were increased to 46.9%.

**Figure 3:**
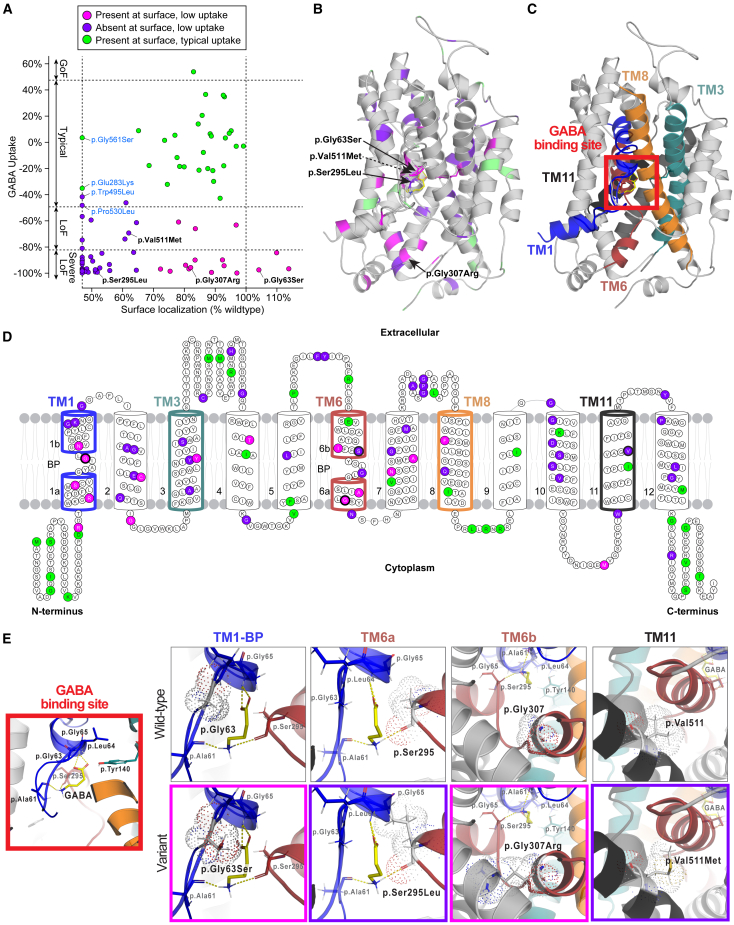
Clustering of surface localization and GABA uptake results of 86 missense variants and highlighted variants in GAT-1 structure and binding site (A) Individual GABA uptake values (y axis) correlated to surface localization results (x axis) and classified by K-means clustering as either present at the surface and low uptake (magenta), absent at the surface and low uptake (purple), or present at the surface and typical uptake (green). Variants with names in blue denote outliers. All other called variants are further illustrated in (B) and (E). (B) GAT-1 3D structure in its inward-open conformation with highlighted variants by cluster. (C) GAT-1 3D structure repeated and highlighted by TM domains. The red square indicates the binding site. (D) 2D topology of GAT-1 structure showing individual variants colored by cluster and corresponding TM domains highlighted from (C). (E) The GAT-1 binding site inset from (C) (red square) and relevant amino acids is shown with GABA bound. The WT and variant 3D structures of four variants are shown, and the variant insets are boxed in colors relevant to their clustering from (A) (magenta or purple). Two variants form part of the binding site for GABA (p.Gly63Ser and p.Ser295Leu), one variant is part of TM6 (p.Gly307Arg), and the variant in TM11 is the top recurrent variant (p.Val511Met, *n* = 6) with available surface localization data. Abbreviations: GoF, gain-of-function; LoF, loss-of-function; TM1, TM domain 1; TM3, TM domain 3; TM6, TM domain 6; TM8, TM domain 8; TM11, TM domain 11.

GABA uptake data were correlated with surface localization results (R^2^ = 0.23, *p* = 3 × 10^−6^; Figure 3A). Variants with typical GABA uptake generally had high surface localization, while loss-of-function variants had highly variable surface localization. To interpret these relationships, we applied a K-means algorithm to define three groups of variants (K = 3; Figure 3A): group 1 variants present on the surface with typical uptake (*n* = 30), group 2 variants absent from the surface with low uptake (*n* = 40), and group 3 variants that were present on the surface but had low uptake (*n* = 16). Four variants with typical GABA uptake were outliers with very low surface localization: c.1681G>A (p.Gly561Ser) (PCC = 0.11), c.847G>A (p.Glu283Lys) (PCC = 0.12), and c.1589C>T (p.Pro530Leu) (PCC = 0.06) identified in a large-scale epilepsy sequencing project^24^ and c.1484G>T (p.Trp495Leu) (PCC = 0.09), a *de novo* variant identified in schizophrenia^4^ (Figure S11). In groups 1 and 2, a substantial fraction of the variability in GABA uptake can be explained by relative surface localization (R^2^ = 0.69, *p* = 8 × 10^−19^) but a different mechanism is required to explain the loss-of-function effects of the 16 missense variants in group 3.

### Mapping of 86 variants to human GAT-1 structure

The human GAT-1 transporter is a 599 amino acid protein that is a member of the solute carrier 6 (SLC6) transporter family, also known as sodium and chloride (Na^+^/Cl^−^) coupled transporter proteins. The SLC6 family contains 20 transporter members that are further divided into four subgroups based on substrate selectivity and sequence homology. This includes the neurotransmitter transporters *SLC6A3* (DAT, dopamine transporter) and *SLC6A4* (SERT, serotonin transporter), which share ∼50% sequence identity to GAT-1. Like all other SLC6 family members, GAT-1 shares the conserved 12 TM domain conformation with amino (N-) and carboxyl (C-) termini located intracellularly.^32^ Previous studies have mapped out important GAT-1 substrate binding site components using the leucine transporter LeuT as a structural model, a bacterial ortholog of the eukaryotic Na^+^/Cl^−^ transporters.^33^^,^^34^ From this model, the main four TM domains that form the binding pocket, or cylindrical ring, of GAT-1 are identified as TM1, TM3, TM6, and TM8. The most conserved regions across the Na^+^/Cl^−^ transporter family include TM1 and TM6, reflecting their importance in binding substrates and inhibitors.^33^ Both TM1 and TM6 helices have unwound “hinge regions” that separate the helices, causing a split of these regions, with the two halves labeled TM1a/TM1b and TM6a/TM6b (Figure 3D). These hinge regions are the formal binding site for GAT-1 substrates and its co-transported ions sodium (2 Na^+^) and chloride (Cl^−^). More recently, the cryo-EM structure of WT human GAT-1 was determined in the inward-open conformation, giving additional insight into specific binding modalities and GAT-1 mechanics.^17^ We mapped all 86 variants to the cryo-EM-derived structure (PDB: 7SK2) and closely analyzed structure-function relationships to determine if this could provide insight into the variants we identified in group 3 (Figures 3B–3D).

Considering the 3D structure (Figures 3B and 3C), we visually calculated the number of variants located on the outer surface of GAT-1 versus the inner surface for each group^35^ (Figure S12, Video S1). We distinguished that 80% of group 2 variants (low uptake and poor protein trafficking) and 93.8% of group 3 variants (low uptake but proper protein trafficking) were found buried in the interior of the 3D structure, in contrast to only 36.4% of variants from group 1 (typical uptake and proper protein trafficking). This suggests that variants positioned within the internal structure can have variable impact on protein trafficking but are more likely to lead to reduced function of GAT-1, whereas variants that fall on the outer surface of the protein structure, and more notably the unwound intracellular and extracellular loops (group 1; Figures 3D and S12A), are less likely to cause deficits in protein trafficking or GABA uptake. Additionally, of the 16 variants in group 3, 13 were located within TM domains, and eight of these are found in the TM domains most closely associated with the GABA binding pocket of GAT-1 (TM1, TM3, TM6, and TM8; Figure 3D), strongly suggesting that their loss-of-function effects were through disruption of this binding modality rather than protein trafficking to the membrane.

Video S1. Loss-of-function variants on rotational GAT-1 structure with GABA docked Severe loss-of-function and loss-of-function variants are highlighted (red and orange, respectively).

To assess this hypothesis, variants within the TM domains that form the binding pocket and main intracellular gate (TM1 and TM6, c.187G>A [p.Gly63Ser], c.884C>T [p.Ser295Leu], and c.919G>A [p.Gly307Arg]) were analyzed in more detail considering the 3D structure (Figure 3E). Variant p.Gly63Ser (group 3), a recurrent *de novo* variant seen in two individuals with seizures, severe DD, and ASD, forms part of the TM1 GABA binding site (Figure 3E). The WT residue (p.Gly63) is predicted to form a hydrogen bond with the carboxylic acid of GABA.^17^ When altered to p.Gly63Ser, a polar functional group is added, increasing strain on the binding site, which may interfere with the native hydrogen bonds to GABA and reduce uptake. Variant p.Ser295Leu (group 2) also forms part of the GABA binding site but within TM6. In its WT form, p.Ser295 forms hydrogen bonds with the backbone of GABA at the amine (-NH_2_). The polar to nonpolar p.Ser295Leu variant is likely to weaken this GABA and/or ion binding. Furthermore, the change to the longer functional group in leucine likely causes substantial strain that interferes with the TM1 helix (Figure 3E), potentially impairing protein trafficking and disrupting the opening and closing mechanics of the transporter necessary for GABA uptake. This same phenomenon can be seen in the variant p.Gly307Arg (group 3) where changing glycine with the positively charged side chain from arginine creates a large strain and interference between TM6 (red) and TM2 (gray).

Additionally, we examined the most recurrent missense variant that was available within these 86 variants (c.1531G>A [p.Val511Met], group 2, *n* = 6; Table 1) to investigate whether its position outside of the binding pocket (TM11) might suggest an additional role underlying the observed recurrence. Interestingly, the addition of a sulfur group gives rise to one of the most hydrophobic amino acids, methionine, and is positioned within TM11. Logically, we would not expect such a substitution, where both WT and mutant have amino acids with hydrophobic side chains, to impact protein trafficking as we observed. Hydrophobic amino acids are known to interact with hydrophobic ligands such as lipids or the plasma membrane.^36^ No clear explanation for recurrence was observed from this analysis.

**Table 1:** Recurrent *de novo* mutations in *SLC6A1*

***SLC6A1* variant**	**GAT-1 variant**	**Count**	**Seizures**	**EMAS**	**Absence**	**Mean seizure onset**	**Develop-mental delay**	**ASD**	**Schizo- phrenia**	***De novo***	**Inherited**	**GABA uptake**	**Mutability**
c.863C>T	p.Ala288Val	11	yes	yes	yes	25 months	yes	yes	–	yes	yes	−83.0%	0.04
c.1024G>A	p.Val342Met	10	yes	yes	yes	28 months	yes	yes	–	yes	yes	−55.7%	0.04
c.1531G>A	p.Val511Met	6	yes	–	yes	12 months	yes	–	–	yes	yes	−69.4%	0.04
c.130C>T	p.Arg44Trp	5	yes	yes	yes	28 months	yes	–	–	yes	–	−96.8%	0.06
c.331G>A	p.Gly111Arg	4	yes	–	–	–	yes	–	–	yes	–	−97.5%	0.06
c.889G>A	p.Gly297Arg	4	yes	yes	yes	31 months	yes	yes	–	yes	–	−96.1%	0.06
c.913G>A	p.Ala305Thr	4	yes	–	–	–	yes	–	yes	yes	yes	−84.5%	0.04
c.1070C>T	p.Ala357Val	4	yes	yes	yes	17 months	yes	–	–	yes	–	−90.6%	0.04
c.1084G>A	p.Gly362Arg	4	yes	–	yes	48 months	yes	–	–	yes	–	−81.1%	0.06
c.1648G>A	p.Gly550Arg	4	yes	–	–	–	yes	yes	–	yes	–	−97.4%	0.06

Variants were mapped to ENST00000287766.10, GenBank: NM_003042.4. EMAS, epilepsy with myoclonic-atonic seizures; ASD, autism spectrum disorder.

Taken altogether, our data suggest that variants clustered within the binding pocket of GAT-1 can properly traffic to the membrane but have poor GABA uptake due to a disruption in substrate binding mechanics (group 3). Additionally, those variants that are buried within the inner surfaces of the protein are more likely to impact protein trafficking to the membrane (group 2) over variants found on the outer surface of the protein (group 1), which have minimal impacts on GABA uptake or protein trafficking. As an additional metric, protein stability algorithms deepDDG^37^ and DynaMut^38^ were performed on GAT-1, and their score outputs were analyzed against our functional data; we did not observe such correlations (Table S2).

### Prenatal lethality, gene-level mutability, and dominant-negative effects do not contribute to missense enrichment

Our functional data strongly suggest that loss-of-function effects underlie seizures, DD, and ASD phenotypes, excluding the possibility of undiscovered gain-of-function variants and complex genotype-phenotype relationships as explanations for the enrichment for missense variants observed in *SLC6A1*. We next considered whether PTVs might increase the rate of prenatal lethality leaving a disproportionate number of missense variants in cohorts of children with neurodevelopmental disorders. Adjusting for gene length and DNA sequence, we find the rate of *de novo* PTVs in *SLC6A1* is equivalent to that of other NDD-associated genes (Figure S13A), suggesting it is an excess of *de novo* missense variants rather than a deficit of *de novo* PTVs driving the enrichment. However, the predicted mutability of *SLC6A1* for both missense and PTV variants is equivalent to that of other NDD genes (Figure S13B). As further evidence against prenatal lethality, the diagnostic rates of seizures, DD, and ASD are similar for *de novo* PTVs (92%, 60%, and 100%, respectively) and *de novo* missense (81%, 53%, and 98%, respectively); furthermore, other genes associated with neurodevelopmental delay have a greater impact on early developmental milestones.^39^

We next considered whether missense variants might lead to an ascertainment bias driven by more severe symptoms due to a dominant-negative effect, as observed in *SLC30A2*.^16^ We tested this hypothesis by assessing co-transfections of WT and missense variants for two missense variants with typical GABA uptake (c.129C>A [p.Asp43Glu] and c.1302C>G [p.Ile434Met]) and five severe loss-of-function missense variants (p.Gly63Ser, c.419A>G [p.Tyr140Cys], p.Ser295Leu, c.1640T>G [p.Leu547Arg], and c.1648G>A [p.Gly550Arg]). None of the variants showed evidence in keeping with dominant-negative effects via co-transfection and uptake assays (Figure S14).

### GAT-1 vulnerability to missense variations explains missense enrichment

**Figure 4:**
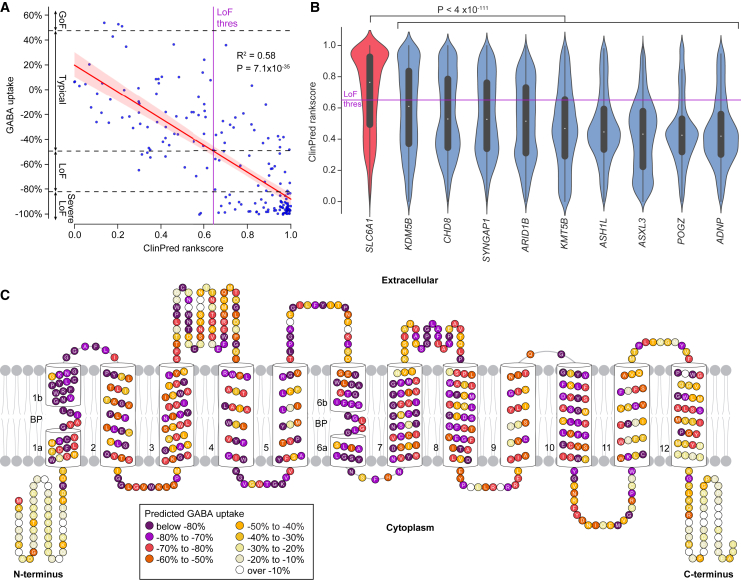
GAT-1 vulnerability underlies the enrichment of *SLC6A1* missense variants (A) Correlation of individual GABA uptake data and ClinPred rankscore for 180 *SCL6A1*/GAT-1 missense variants. The red line shows the linear regression model with 95% confidence intervals represented by the red shading. The ClinPred value at the estimated loss-of-function threshold is indicated by the purple line (“LoF thres”). (B) Violin plots represent the ClinPred rankscore distribution for all possible missense variants in *SLC6A1* and nine equivalent PTV-enriched ASD- and NDD-associated genes. The LoF thres line from (A) is also shown. (C) All 599 amino acids of GAT-1 are colored by the mean predicted GABA uptake of all possible missense variants using the linear regression model from (A) from the annotated ClinPred rankscores. Abbreviations: GoF, gain-of-function; LoF, loss-of-function. Statistical tests: A, linear regression; B, two-sided Wilcoxon test.

The GAT-1 transporter is a highly specific, dynamic, and complicated molecular machine.^17^ Could the intricate protein structure render the protein unusually susceptible to loss-of-function missense variation? Exploring this possibility would require extrapolating the GABA uptake results from the 180 assayed missense variants to all possible missense variants for the gene. Multiple algorithms have been developed to estimate the functional impact of missense variants, largely based on conservation across species and constraint in human populations. We used ANNOVar to annotate the 180 missense variants against the output of multiple such algorithms (Table S2) and stepwise linear regression (Table S6) and random forest machine learning (Table S7) to build a predictive model. The R^2^ was above 0.55 for three linear regression models independently, ClinPred: R^2^ = 0.58, *p* = 7 × 10^−35^ (Figure 4A),^40^ MetaSVM: R^2^ = 0.57, *p* = 8 × 10^−34^,^41^ and MetaRNN: R^2^ = 0.55, *p* = 1 × 10^−32^,^42^ of note, all three are ensemble models integrating multiple other algorithms trained against large databases, such as ClinVar. Selecting ClinPred and adding additional predictors (MetaSVM,^41^ fitCons,^43^ phyloP30way mammalian,^44^ and LIST-S2^45^) only marginally improved the prediction to R^2^ = 0.62 probably because of the high correlations between these algorithms (Figure S15). Using a random forest approach to model fitting for the 180 missense variants yielded an R^2^ = 0.55. Given the risk of overfitting to a relatively small dataset, we elected to use the ClinPred model with linear regression alone (scaled from no predicted impact at 0 to severe predicted impact at 1).

To select comparator genes, we identified nine genes with equivalent evidence of association with ASD (FDR ≤ 1 × 10^−10^) and NDD (FDR ≤ 1 × 10^−10^) that were enriched for PTVs (*de novo* PTV count/*de novo* missense count >2)^2^ and had ClinPred scores available. For *SLC6A1* and these nine genes, we used the longest protein-coding isoform from GENCODE v33 to predict all possible missense variants and plotted the ClinPred score distributions (Figure 4B). In keeping with a higher vulnerability to missense variation, ClinPred scores are substantially higher for *SLC6A1* than for the other nine genes, with a median of 0.76 for *SCL6A1* versus 0.60 for *KDM5B* (*p* = 4 × 10^−111^), the next highest gene (Figure 4B). In NDD, we observed a 7-fold enrichment of *de novo* missense variants compared to *de novo* PTVs (43 missense, 6 PTVs, ratio = 7.17).^2^ Based on a ClinPred score of ≥0.642, set by the loss-of-function threshold (Figure 4A), we observed the total number of predicted loss-of-function missense sites to be 7-fold higher than the total number of predicted PTV missense sites (2,481 missense, 349 PTVs, ratio = 7.11; Table S7) with a similar ratio after adjusting for mutability (ratio = 7.06), suggesting the magnitude of this missense vulnerability is sufficient to explain the observed missense enrichment. Other measures of missense severity highly correlated to the functional data yield similar patterns (Figure S16).

Using the linear model to convert the ClinPred scores to estimated GABA uptake, we plotted the mean functional impact for each of the 599 amino acids in GAT-1 (Figure 4C). In keeping with the 3D structure results, we see substantial vulnerability throughout TM domains 1 to 10, along with some of the cytoplasmic and extracellular loops. In contrast, the N- and C-terminus loops show limited vulnerability to missense variants, consistent with our directly assayed functional data (Figure 1A).

### Recurrent missense variants in *SLC6A1* are at hypermutable sites

**Figure 5:**
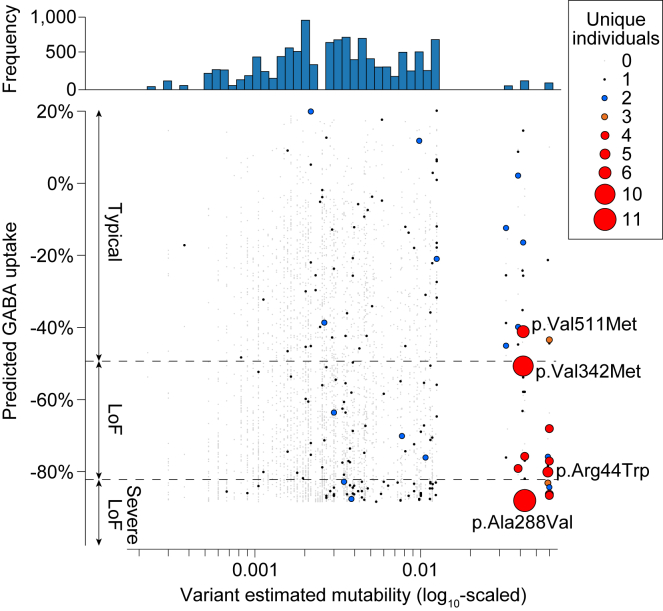
Recurrent missense variants in *SLC6A1* are at hypermutable loci Estimated mutability based on three base-pair DNA sequences is shown for all 3,952 possible missense variants in *SLC6A1* (x axis). A small number of variants have a ∼10-fold higher mutation rate due to the presence of CpG, as shown by the histogram (top). Predicted GABA uptake is predicted for each of these missense variants based on ClinPred (Figure 4). The number of unique individuals is indicated by size and color for each variant; the four most frequent are labeled (Figure 2; Table 1). LoF, loss-of-function.

GAT-1 vulnerability can account for the enrichment of missense variants in *SLC6A1*; however, this still does not account for the observation of recurrent *de novo* missense mutations (Table 1; Figures 2 and S1). To assess whether mutability may be a factor, we estimated the mutability of all possible missense variants in *SLC6A1* and plotted these estimates against the predicted GABA uptake from ClinPred (Figure 5). A small fraction of missense variants in *SLC6A1* (75 out of 3,943, 1.9%) are at CpG sites that are known to be hypermutable^46^ due to the spontaneous deamination of 5-methylcytosine (5mC) to thymine (Figure 5). However, 22 of the 31 recurrent missense variants (71.6%) and all 14 (100%) of the missense variants identified in three or more individuals are in this small hypermutable subset (Figures 5 and S17). The majority of these are at sites we predict to be loss-of-function.

## Discussion

*SLC6A1* is a promising therapeutic target due to its strong association with neurodevelopmental disorders and seizures and the clear role of encoded GAT-1 in transporting GABA. However, translational progress requires a clear understanding of genotype-phenotype relationships. Some individuals have PTVs, suggesting autosomal dominant loss of function (haploinsufficiency, i.e., insufficient GABA transport) as the mechanism; however, the vast majority of variants are missense. This enrichment of missense variants, combined with multiple recurrent missense mutations, implies a gain-of-function or dominant-negative mechanism (i.e., some missense variants have a greater impact on phenotype than PTVs). The application of a therapy designed to potentiate GABA uptake in a person with a gain-of-function variant could have devastating consequences.

By performing a comprehensive functional screen for *SLC6A1*, we see clear evidence that a reduction in GABA uptake underlies neurodevelopmental and seizure symptoms. Of the 66 germline *de novo* variants assessed, 64 (97.0%) resulted in loss of function (Figure 2E), and the remaining two showed a trend toward reduced GABA uptake (−20.2% for c.1096C>G [p.Leu366Val], −41.7% for c.1484G>T [p.Trp495Leu]). This consistent functional result, alongside the observation that seizures, DD, and ASD are often co-morbid in the same individual, supports a common mechanism of reduced GABA uptake leading to seizures. Associated seizure types include EMAS, EOAE, CAE, DEE, and LGS but not NAFE (Figure S8). Of note, no association was seen between the age of seizure onset and the degree of GABA uptake impairment (Figure S8), and other metrics of phenotypic severity were not available for assessment. While the results are suggestive that reduced GABA uptake contributes to schizophrenia risk (Figures 2E and S8), further genomic, functional, and longitudinal phenotyping will be required for definitive answers.

In contrast to loss of function, we find limited evidence for phenotypic effects from variants that increase GABA uptake (e.g., gain of function). Nine missense variants were estimated to increase in GABA uptake by ≥ 20% (Figure 1); however, analysis of ten variants using stable transfections, a more accurate method, showed more modest changes, ranging from 8% to 28% over WT (Table S4). While several of these variants that modestly increase GABA uptake are identified in individuals with seizures, none of them are reported to have EMAS, none are classified as pathogenic/likely pathogenic in ClinVar, none are known to be *de novo*, and most are observed in the general population (gnomAD). Based on these data, the neurodevelopmental risk mediated by these variants is probably very small, if any. We considered the possibility that increased GABA uptake might have a protective effect. Genome-wide association studies (GWASs) for epilepsy do not identify significant association at the *SLC6A1* locus^47^; however, the allele frequency of these variants remains below the GWAS detection threshold of ∼2% AF.

While the functional data provide a clear answer for the impact on GABA uptake, they do not account for the observed enrichment of missense variants compared with other neurodevelopmental genes; failure to explain this phenomenon could reduce confidence in future therapies aiming to increase GABA uptake. Co-transfection of WT and missense variants did not find evidence of dominant-negative effects (Figure S14), and genomic and phenotype data do not support prenatal lethality from PTVs. In contrast, using computational predictors of missense severity to extrapolate the functional data to all possible missense variants provides a clear answer: *SLC6A1* is simply more sensitive to disruption by missense variants than many genes. This observation also explains the relatively high frequency of *SLC6A1*-related disorders in large cohorts despite the comparatively small size of the gene (599 amino acids). While missense sensitivity can explain the missense enrichment, at face value it does not account for the recurrent missense variants. However, integrating mutability data, we observe that these can be explained by a small number of missense variants that both reduce GABA uptake and occur at highly mutable CpG sites. This missense sensitivity may also explain other genes enriched for missense variants despite predominantly loss-of-function effects, such as *SCN1A* (Dravet syndrome).

Our functional data provide “strong” information to guide clinical interpretation based on the ACMG guidelines (specifically, categories PS3 and BS3). Of the 213 variants we tested, 163 (76.5%) did not have ACMG classifications (e.g., likely pathogenic) in the ClinVar database (Figure 2D). Considering the 127 we identified as loss of function or severe loss of function, only 30 (23.6%) had previously been classified as pathogenic or likely pathogenic with the remainder being unscored (66, 52.0%), uncertain significance (18, 14.2%), conflicting interpretations (10, 7.9%), or likely benign (3, 2.4%). In contrast, of the 86 variants that did not show significant loss of function, only 15 (17.4%) were classified as benign or likely benign with the remainder being unscored (35, 40.7%), uncertain significance (28, 32.6%), conflicting interpretations (6, 7.0%), likely pathogenic (1, 1.2%), or pathogenic (1, 1.2%) (Figure 2D; Table S2).

Overall, for the 60 variants that were scored, only three had opposing clinical and functional interpretations, recorded as likely benign in ClinVar, but observed to be loss-of-function for GABA uptake: c.424G>A (p.Val142Ile) (−50.4% GABA uptake), c.1249C>T (p.Arg417Cys) (−63.5%), and c.1391T>A (p.Val464Glu) (−61.5%). All three variants were submitted to ClinVar by the same submitter without additional evidence for the variant being benign. The associated condition was EMAS/MAE and therefore consistent with *SLC6A1* haploinsufficiency. The clinical interpretation was not supported by the functional data for two further variants: p.Leu366Val (likely pathogenic, −20.2% GABA uptake, not in gnomAD, germline *de novo* in an individual with DD, but no further clinical details) and c.967G>A (p.Val323Ile) (pathogenic, −30.0%, rare in gnomAD [7 × 10^−6^], unknown inheritance in an individual with DD, but no further clinical details). We note that our loss-of-function threshold of −49.2% is probably conservative and that milder impairments may be clinically relevant, especially on a sensitized genetic background.

To gain further insight into the functional impact of *SLC6A1* variants causing these phenotypes, we studied whether there were any structure-function relationships. Previous studies have shown that variants in SLC6 family members, including *SLC6A1*, can cause a disruption in protein trafficking to the membrane, and thus impact function and lead to disease.^10^^,^^12^^,^^48^^,^^49^ Our study demonstrated a broad clustering of functionally studied variants by whether or not they were detected on the plasma membrane through high-content imaging of GFP fluorescence. We showed that variants clustered in three distinct groups (Figure 3A): group 1 variants present on the cell surface with typical uptake (*n* = 30), group 2 variants absent from the cell surface with low uptake (*n* = 40), and group 3 variants that were present on the cell surface but had low uptake (*n* = 16). Notably, group 3 variants that showed proper protein trafficking and low transporter function were clustered within the binding pocket of GAT-1 and/or the interior of the 3D structure. This suggests that variants in these structural locations are disrupting proper substrate binding and/or transport mechanics (Figures 3D and 3E). Those in group 2 (low uptake and low cell surface localization) shared similar mapping on the 3D structure to group 3, found mainly in positions embedded within the inner surface of the protein and on TM domains (Figures 3D, S12B, and S12D). This shows the importance of those regions, most notably the TM domains, in proper transporter function and perhaps stability and proper folding before trafficking.

Understanding where variants are structurally located and whether they impact trafficking is important for the design of future therapies. For example, before proteins can traffic to their destination, they must properly fold post-translation, and in the presence of variants, this folding can be disrupted. In this case, a therapy that increased the expression of both alleles might have unexpected consequences. Certain SLC6 family members have been previously rescued from folding-deficient and disease-associated variants through the use of pharmaco-chaperoning.^50^ To date, GAT-1 has not been studied enough to understand whether using chaperones could help improve symptoms. Additionally, while we broadly studied the structure-function relationships of a subset of variants, many other pathways influence transport and trafficking mechanisms of GAT-1. This includes N-glycosylation, oligomerization disruption, protein kinase C-mediated phosphorylation, endocytic trafficking, and more.^49^^,^^51^^,^^52^^,^^53^^,^^54^^,^^55^ With the latest advances in GAT-1 structure, new studies need to be conducted to better understand how GAT-1 works in cells mechanistically.

As ever, our experiment has limitations. The functional studies were performed in HEK cells rather than the cell types in the brain that mediate symptoms. Also, we only assayed GABA uptake; gain-of-function consequences could include the uptake of another molecule or an entirely orthogonal function altogether. However, gain of function seems unlikely given the specificity of the transporter to GABA, the functional enrichment of TM domains, the limited evidence of phenotypic consequences of variants in the N- and C-termini, and the observation that missense sensitivity and mutability can account for the missense enrichment. Our dominant-negative assay relies on co-transfection assays that are sensitive to relative plasmid concentrations; studies assessing dimerization directly might reach different conclusions.

### Conclusion

In summary, by integrating genomic, functional, and phenotype data, we observe unambiguous evidence that reduced GABA uptake underlies neurodevelopmental sequelae associated with *SLC6A1* variants, including seizures, DD, and ASD. Furthermore, the observed enrichment of missense variants and recurrent missense variants can be explained by the sensitivity of *SLC6A1* to missense variants and mutable CpG sites, respectively. Based on these results, therapeutic strategies for *SLC6A1*-related neurodevelopmental disorders should aim to increase GABA uptake.

## Data and code availability

All data used in these analyses is publicly available and reported in the supplemental tables. The code generated during this study is available at https://github.com/sanderslab/slc6a1.git.

## Supplemental information

Document S1. Figures S1–S17 and supplemental material and methods

Table S1. Individual-level data Excel spreadsheet in which each row represents an individual with data on the SLC6A1 variant and phenotype.

Table S2. Variant-level data Excel spreadsheet in which each row represents a unique variant with data on the SLC6A1 variant and phenotype combined across individuals.

Table S3. Plasmid sequences Excel spreadsheet with four sheets detailing the plasmids used for the GABA uptake and surface expression assays.

Table S4. GABA uptake and surface-expression data Excel spreadsheet with four sheets detailing the results of the GABA uptake and surface-expression assays.

Table S5. ClinVar categories Excel spreadsheet showing the relationship between ClinVar category and GABA uptake.

Table S6. Linear regression Excel spreadsheet with 10 sheets detailing the stepwise linear regression model building for missense variants.

Table S7. All SLC6A1 SNVs Excel spreadsheet showing functional annotation, GAT-1 location, and missense severity scores (including predicted from linear regression model and random forest model) for all possible SNVs in SLC6A1 cDNA.

Document S2. Article plus supplemental information
