# Supplementary material for: Haploinsufficiency underlies the neurodevelopmental consequences of SLC6A1 variants
Source: Am J Hum Genet. 2024 May 22;111(6):1222–38. doi: 10.1016/j.ajhg.2024.04.021 (PMC11179425; doi:10.1016/j.ajhg.2024.04.021)
Supplement: Document S2. Article plus supplemental information [file mmc10.pdf]

Haploinsufficiency underlies the neurodevelopmental consequences of *SLC6A1* variants

Graphical abstract

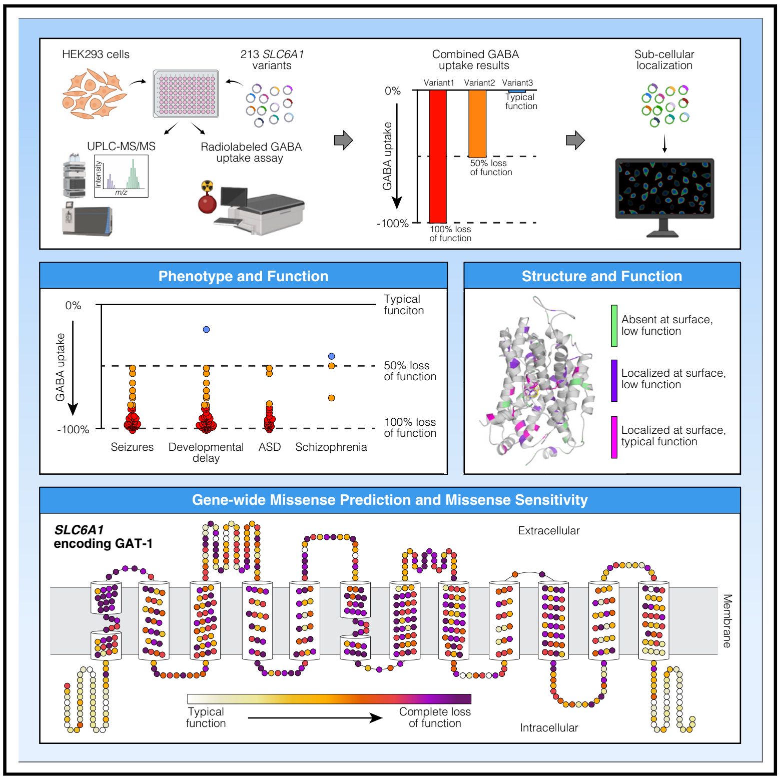

Authors

Dina Buitrago Silva, Marena Trinidad, Alicia Ljungdahl, ..., Steven Froelich, Stephan J. Sanders, Arthur Wuster

Correspondence

stephan.sanders@paediatrics.ox.ac.uk (S.J.S.),
arthur.wuster@bmrn.com (A.W.)

Germline *de novo* variants in *SLC6A1* are a major cause of neurodevelopmental disorders, including seizures. Enrichment for missense variants, many of which are recurrent, suggests a gain-of-function mechanism. However, here, we show that such variants consistently decreased GABA uptake *in vitro*, supporting a haploinsufficiency mechanism underlying the observed neurodevelopmental phenotypes.

Silva et al., 2024, The American Journal of Human Genetics *111*, 1222–1238
June 6, 2024 © 2024 The Authors.
https://doi.org/10.1016/j.ajhg.2024.04.021

ARTICLE

Haploinsufficiency underlies the neurodevelopmental consequences of *SLC6A1* variants

Dina Buitrago Silva,[1,11] Marena Trinidad,[2,3,4,11] Alicia Ljungdahl,[5,6] Jezrael L. Revalde,[7] Geoffrey Y. Berguig,[2] William Wallace,[2] Cory S. Patrick,[5] Lorenzo Bomba,[2] Michelle Arkin,[7] Shan Dong,[5] Karol Estrada,[2] Keino Hutchinson,[8] Jonathan H. LeBowitz,[2] Avner Schlessinger,[8] Katrine M. Johannesen,[9] Rikke S. Møller,[9,10] Kathleen M. Giacomini,[1] Steven Froelich,[2] Stephan J. Sanders,[5,6,*] and Arthur Wuster[2,*]

Summary

Heterozygous variants in *SLC6A1*, encoding the GAT-1 GABA transporter, are associated with seizures, developmental delay, and autism. The majority of affected individuals carry missense variants, many of which are recurrent germline *de novo* mutations, raising the possibility of gain-of-function or dominant-negative effects. To understand the functional consequences, we performed an *in vitro* GABA uptake assay for 213 unique variants, including 24 control variants. *De novo* variants consistently resulted in a decrease in GABA uptake, in keeping with haploinsufficiency underlying all neurodevelopmental phenotypes. Where present, ClinVar pathogenicity reports correlated well with GABA uptake data; the functional data can inform future reports for the remaining 72% of unscored variants. Surface localization was assessed for 86 variants; two-thirds of loss-of-function missense variants prevented GAT-1 from being present on the membrane while GAT-1 was on the surface but with reduced activity for the remaining third. Surprisingly, recurrent *de novo* missense variants showed moderate loss-of-function effects that reduced GABA uptake with no evidence for dominant-negative or gain-of-function effects. Using linear regression across multiple missense severity scores to extrapolate the functional data to all potential *SLC6A1* missense variants, we observe an abundance of GAT-1 residues that are sensitive to substitution. The extent of this missense vulnerability accounts for the clinically observed missense enrichment; overlap with hypermutable CpG sites accounts for the recurrent missense variants. Strategies to increase the expression of the wild-type *SLC6A1* allele are likely to be beneficial across neurodevelopmental disorders, though the developmental stage and extent of required rescue remain unknown.

Introduction

Large-scale exome sequencing studies have identified the gene *SLC6A1* (solute carrier family 6 member 1, ENSG00000157103), which encodes the GABA transporter protein "GAT-1," as a major cause of neurodevelopmental disorders (NDD). Genome-wide significant association has been reported for rare heterozygous variants in independent cohorts of developmental delay (DD),[1] autism spectrum disorder (ASD),[2] and pediatric-onset epilepsy, particularly epilepsy with myoclonic-atonic seizures (EMAS, previously myoclonic-atonic seizures or MAE)[3]; it has also been implicated in schizophrenia.[4,5] Across these disorders, the incidence related to *SLC6A1* variants is estimated to be 2.4–2.9 in 100,000 births,[6] making it a relatively common single-gene disorder. These features make *SLC6A1*, encoding GAT-1, a promising target for novel therapeutics.

Realizing the therapeutic potential of *SLC6A1* requires understanding the functional impact of the genetic variants observed in different phenotypes. GAT-1 is predominantly found embedded in the cell surface membrane and transports the inhibitory neurotransmitter GABA from the synaptic cleft into presynaptic neurons,[7] which may limit the inhibition of postsynaptic neurons and prepare the presynaptic neuron for further GABA release. *SLC6A1* is very highly expressed in *SV2C*/*LAMP5*-expressing GABAergic (inhibitory) neurons, highly expressed in other classes of GABAergic neurons (expressing *PVALB*, *VIP*, or *SST*), and weakly expressed in multiple non-neuronal cell types, including astrocytes, oligodendrocytes, oligodendrocyte precursor cells, and endothelial cells[8]; there is minimal expression in excitatory glutamatergic neurons. It is widely expressed across brain regions, with expression increasing rapidly during mid to late fetal development, especially in the striatum, before reaching a steady state from birth to late adulthood.[9]

The majority of *SLC6A1* variants associated with human disorders are predicted to be missense variants or

[1]Department of Bioengineering and Therapeutic Sciences, University of California, San Francisco, San Francisco, CA, USA; [2]BioMarin Pharmaceutical Inc., Novato, CA, USA; [3]Department of Molecular and Cell Biology, University of California, Berkeley, Berkeley, CA, USA; [4]Innovative Genomics Institute, University of California, Berkeley, Berkeley, CA, USA; [5]Department of Psychiatry, UCSF Weill Institute for Neurosciences, University of California, San Francisco, San Francisco, CA, USA; [6]Institute of Developmental and Regenerative Medicine, Department of Paediatrics, University of Oxford, Oxford OX3 7TY, UK; [7]Department of Pharmaceutical Chemistry, University of California, San Francisco, San Francisco, CA, USA; [8]Department of Pharmacological Sciences, Icahn School of Medicine at Mount Sinai, New York, NY 10029, USA; [9]Department of Regional Health Research, Faculty of Health Sciences, University of Southern Denmark, Odense, Denmark; [10]Department of Epilepsy Genetics and Personalized Medicine, Member of ERN Epicare, Danish Epilepsy Centre, Dianalund, Denmark
[11]These authors contributed equally
*Correspondence: stephan.sanders@paediatrics.ox.ac.uk (S.J.S.), arthur.wuster@bmrn.com (A.W.)
https://doi.org/10.1016/j.ajhg.2024.04.021.
© 2024 The Authors. This is an open access article under the CC BY license (http://creativecommons.org/licenses/by/4.0/).

in-frame indels, some of which are recurrent *de novo* mutations (e.g., 11 individuals with c.863C>T [p.Ala288Val] and 10 individuals with c.1024G>A [p.Val342Met], Figure S1). This distribution is highly suggestive of a gain-of-function or dominant-negative mechanism, as has been observed in numerous other rare genetic disorders across many phenotypes,[1–3] and has motivated a clinical trial to inhibit GAT-1 in schizophrenia.[4] Conversely, there are also multiple individuals with protein-truncating variants (PTVs, including stop gain, frameshift, and canonical splice site variants), suggesting a co-existing loss-of-function mechanism. The function of 29 *SLC6A1* variants has previously been characterized using GABA uptake assays, including four PTVs resulting in complete loss of GABA uptake and 25 missense/in-frame variants with varying degrees of loss-of-function[10–14] (Table S2). Given the loss-of-function outcomes observed, it remains unclear why *SLC6A1* is strongly enriched for missense/in-frame variants rather than the PTVs with clear loss-of-function mechanisms that are observed in most genes associated with neurodevelopmental disorders.[2] Possibilities include undiscovered gain-of-function variants, genotype-phenotype relationships that vary by disorder, as observed in *SCN2A*,[15] prenatal lethality, or a dominant-negative effect, as observed in *SLC30A2*.[16]

Here, we present data on the functional impact on GABA uptake of 213 *SLC6A1* variants, 185 of which have not previously been characterized,[10–14] including variants associated with schizophrenia, the majority of recurrent variants, and "control" variants that are synonymous, common, or documented as benign in ClinVar. We assess the surface localization of 86 of these variants and explore the results in the context of the previously published GAT-1 structure[17] with updated annotations of transmembrane (TM) domains, and intra- and extra-cellular loops. In addition, we explore the possibility of gain-of-function effects in stable lines for ten variants and dominant-negative effects for seven variants. Finally, through integrative analysis of genomic data, we demonstrate that GAT-1 is "delicate" so that multiple missense variants can lead to clinically relevant haploinsufficiency. This leads to a parsimonious model of *SLC6A1*-related disorders via a heterozygous loss-of-function mechanism with the observed missense enrichment explained by GAT-1 missense sensitivity and the recurrent missense variants explained by hypermutable CpG loci.

Material and methods

Variant selection and functional assays were performed independently by teams at BioMarin and UCSF before integrating the data for a combined analysis of 213 unique variants. Combined methods are described below, with distinctions highlighted between groups as required. For detailed methods from each research group please see supplemental material and methods at the end of this manuscript.

Variant selection

Individuals with variants in *SLC6A1* were identified from multiple sources, including ClinVar,[18] gnomAD,[19] multiple cohort studies,[1–4] case series and reports,[20] and three previously undescribed individuals from the Møller group in Denmark. Across these sources, 400 unique individuals were identified in total (Table S1), mapping to 323 unique variants in individuals plus a further ten common missense variants from gnomAD (Table S2). Variants were selected for functional interpretation based on recurrence across multiple individuals, variants in individuals with detailed phenotyping data, distribution across phenotypes, distribution across GAT-1, distribution of predicted effect on GAT-1, and distribution of population frequency. The BioMarin group assayed 181 variants, the UCSF group assayed 100 variants, and 68 of these variants were assayed by both groups (Table S2). Twenty-four variants were selected as controls, composed of six synonymous variants, ten missense variants observed in multiple individuals in gnomAD population controls,[19] and seven missense variants predicted to be benign from clinical sequencing as reported in ClinVar.[18] The full list of individuals and phenotypes is reported in Table S1, and the full list of variants, genomic annotations, and functional results are reported in Table S2.

Annotation of *SLC6A1* variants

Variant impact was estimated using the ENST00000287766.10 transcript (RefSeq: NM_003042.4) for the ENSG00000157103.12 *SLC6A1* as defined by GENCODE v39. All reported *SLC6A1* variants in ClinVar were downloaded on June 22, 2022 to annotate clinical significance. All *SLC6A1* variants in the "non-neuro" version of gnomADv2.1.1 were downloaded on August 18, 2022 to annotate gnomAD population allele frequency. gnomAD variant frequency estimates across all ancestries were used. Predicted missense severity was annotated using ANNOVar protocol "dbnsfp42a" and build "hg38."[21]

Gene constructs and GFP tagging

The *SLC6A1* consensus coding sequence (CCDS: CCDS2603.1, NM_003042) was used for the creation of both gene constructs from each group under the cytomegalovirus promoter, but with different vectors. The resulting wild-type (WT) *SLC6A1* constructs from each group were sent to Genscript (Piscataway, NJ) to perform site-specific mutagenesis to generate 181 distinct variants (BioMarin team) and 100 distinct variants (UCSF group). Genscript internally confirmed the quality and sequence of all plasmids, and the expected variant was further confirmed by Sanger sequencing.

For the GFP-tagged plasmids for 86 missense variants, the 249 amino acid monomeric superfolder green fluorescent protein (sfGFP, ASSN: ASL68970) was first synthesized by Genscript. Separately, the UCSF research group sent the WT-SLC6A1 construct back to Genscript, and the sfGFP was cloned into the C-terminal end of the *SLC6A1* transcript with no spacer or linker. The final plasmid, WT-SLC6A1-sfGFP, was verified by Genscript and tested in the [^{3}H]-GABA transport assay to compare relative uptake to WT-SLC6A1. No difference in GABA uptake was observed. To create the 86 GFP-tagged variant plasmids, Genscript performed site-directed mutagenesis on the WT-SLC6A1-sfGFP plasmid and verified every individual variant by Sanger sequencing.

Preparation of cell lines and transfection of variants

Both research groups prepared human embryonic kidney cells (HEK293) separately to study the functional effects of *SLC6A1*

variants. The BioMarin team employed CRISPR/Cas9 to generate a *SLC6A1*-deficient HEK293T cell line (see supplemental material and methods). The GABA uptake assay was used to confirm loss of GAT-1 activity in the knockout line. The UCSF research group used the HEK293 Flp-In integration system and an empty vector (EV) control in every transfection experiment to control for any background GABA uptake in the cells (see supplemental material and methods). The [^{3}H]-GABA transport assay was used before experimental design to compare background activity (EV) to WT activity (WT-*SLC6A1*). Plasmid sequences are reported in Table S3.

GABA uptake assay and [^{3}H]-GABA transport assay

The BioMarin research team employed ultra-high performance liquid chromatography-MS/MS (UPLC-MS/MS) in their experimental design to functionally characterize controls and variants. Beta-lactamase (BLA) activity was measured to account for transfection variability. The UCSF research group used a radiolabeled uptake assay to characterize GABA transport of variants and controls. Both groups used the Pierce BCA Protein Assay (Thermo Fisher Scientific, Cat. 23227) to measure total protein in each well, accounting for plating variability. Refer to the supplemental material and methods for further detailing of each assay.

Combining uptake data across cohorts

For each cohort (BioMarin and UCSF) the data from the three to six replicates (Table S4) that passed quality control (count) was used to estimate the mean. Log-transformed mean estimates were compared between the two groups. Based on the strong correlation values observed between the functional data generated by BioMarin and UCSF (Figure S2), we calculated the mean of GABA uptake from the 68 variants where both groups had estimated GABA uptake. We made one exception, the insertion chr3:11020264:T:TA (hg38), c.523_524insA (p.Ser175Tyrfs*32), which is predicted to induce a frameshift variant and premature stop codon in GAT-1. The UCSF data predicted no GABA uptake (−102.4%) while the BioMarin data predicted reduced update (−73.2%). Given that 22 other PTVs had yielded a GABA uptake value below −92.3%, we elected to choose the UCSF value of −102.4% for the combined result.

Defining thresholds for functional impact

The GABA uptake values for 24 control variants (Table S2) were normally distributed around a mean of −0.8% with a standard deviation of 24.7%. We used *Z* score thresholds of 1.96 standard deviations (48.4%, $p \leq 0.05$, two-sided) to define loss-of-function (−0.8% − 48.4% = −49.2%) and gain-of-function (−0.8% + 48.4% = 47.5%) and 3.29 standard deviations (81.2%, $p \leq 0.001$, two-sided) to define severe loss-of-function (−0.8% − 81.2% = −82.1%). No variants were above 80.4%, which would be the equivalent severe gain-of-function threshold.

Immunostaining

For immunostaining experiments, HEK293 cells were plated on poly-D-lysine-treated 96-well black PhenoPlate (PerkinElmer Health Sciences) with an optically clear flat-bottom and at an optimized cell density of 2.4×10^4 cells/well. All wells on the edges of the plate were excluded to prevent edge effects. Cells were reverse transfected with respective SLC6A1-sfGFP-mutant plasmid and controls (WT-SLC6A1-sfGFP and EV-sfGFP). Mutant plasmids were transfected in 3 biological replicates and performed in duplicate. WT and EV were treated the same but had an additional plate with 12 biological replicates per plasmid and performed in duplicate. After 48 h, cells were washed once with Hank's Balanced Salt Solution (HBSS), and then the plasma membrane was stained with Wheat Germ Agglutin Alexa Fluor 647 conjugate (Invitrogen Life Sciences) diluted (1:1000) in cold HBSS for 5 min at room temperature. The stain was then aspirated, and cells were carefully washed three times with HBSS. Cells were fixed with 3.7% formaldehyde in HBSS for 15 min. The fixing solution was then aspirated, and cells were washed again three times with HBSS. Next, the cytoplasm was stained using HCS CellMask Orange Stain (Invitrogen Life Sciences) in HBSS (1:1000) for 5 min at room temperature and kept in darkness. The stain was aspirated, and cells were washed two more times with HBSS. Lastly, the nucleus was stained with Hoescht in HBSS (1:2000) for 20 min at room temperature and kept in darkness. Cells were washed twice with HBSS, and 100 μL of buffer was left in the wells for imaging. Imaging was performed within 24 h of staining or the same day and then stored in a 4°C fridge wrapped in aluminum foil.

High-throughput confocal imaging with IN Cell 6500HS Analyzer and IN Carta Software analysis

Immunostained plates were imaged with the IN Cell Analyzer 6500HS (GE Healthcare, WA, USA). All images were taken with a Nikon 40× objective with the laser and software autofocus (AF) applied. A total of eight fields of view were captured per well across all plates ($n = 48$ total per plasmid: eight fields of view, three biological replicates, performed twice). Four channels were used to capture the respective stain or fluorescence. The blue laser (405 nM excitation and 455/50 nM emission) captured the Hoechst stain. The orange laser (561 nM excitation and 605/51 nM emission) captured the orange HCS CellMask stain. The far-red laser (642 nM excitation and 682/59 nM emission) captured the Alexa Fluor 647 conjugate. The green laser (488 nM excitation and 524/48 nM emission) captured GFP fluorescence.

The resulting images were visualized and analyzed with the IN Carta image analysis software (Molecular Devices, San Jose, CA). The protocol segmented the images by cell components. The blue wavelength was used to segment the mono-nucleated cells. The orange wavelength was used to segment the whole cell using the orange HCS CellMask. Once the software identified the nucleus and whole cell, the membrane was segmented using the red wavelength. For example images, see Figure S3. The protocol was run, and Pearson correlation values were generated for GFP fluorescence colocalization with the plasma membrane stain (Alexa Fluor 647). Pearson correlation values are calculated through the software using total intensities (total intensity = intensity × cell area) of the respective wavelengths captured in the field of view(s). After all values were generated from this protocol, the classifier tool within the IN Carta software was applied to further refine Pearson correlation raw scores. This tool allows the user to define upper and lower bounds for intensities detected across the entire plate, helping to account for plate-to-plate variability. Lower bounds were classified as "low GFP" fluorescence or background that is detected by the software. Upper bounds were classified as an "overexposed GFP" signal, which is bright puncta detected. Between the upper and lower bounds, there was a "high GFP" signal, and the classifier tool reran the protocol with these newly defined classes. Newly generated values consider Pearson correlation scores by class. All final analyses in this study were conducted based on the "high GFP" class (Figure S4).

Protein structure mapping in 2D and 3D (combined datasets)

To visualize the topology of GAT-1 and percent-wild-type uptake activity of missense variants, the UCSF TM protein display tool TOPO2 was used (https://www.cgl.ucsf.edu/Overview/ftp/topo2.tar.gz). The topological domains of *SLC6A1* were annotated in two different ways (Tables S1 and S2). The initial annotations were taken from UniProt (ID P30532). The second annotations, used for our primary analysis and all topology figures and 3D representations, used the recently published 3D structure of GAT-1.[17] Molecular docking was performed using Glide[22] from the Schrödinger suite. GABA was docked to the cryo-EM structure of GAT-1 (PDB: 7SK2). The ligand binding site was defined based on the coordinates of Tiagabine in the published structure. The docking results, figures, and videos were visualized via PyMOL, version 2.5.

Data analysis and figures

Data were analyzed using Python (including pandas, NumPy, statsmodels, matplotlib, and seaborn libraries). Figures were composed using Adobe Illustrator. Further details on individual analysis can be found under supplemental material and methods.

Results

Impact of 213 variants in *SLC6A1* on GABA uptake activity

A combined search of publications and large-scale genomic cohorts identified 400 individuals with variants in *SLC6A1*, which mapped to 323 unique variants[4,10,11,19,20,23–30] (Table S1). Our two groups (BioMarin, UCSF) independently selected subsets of these variants for functional analysis of GABA uptake using a $[^3H]$-GABA transport assay in HEK293 cells (supplemental material and methods). Assays of GABA uptake are reported as the percent difference from WT, with 0% being the same as WT and −100% being the complete absence of GABA uptake. In total, 213 variants in *SLC6A1* were assessed, and the 68 variants analyzed by both groups yielded consistent results ($R^2 = 0.79$, $p = 5.3 \times 10^{-24}$, Figure S2). Our data were also consistent with previously published results ($n = 21$; $R^2 = 0.51$,[12] $n = 7$; $R^2 = 0.67$,[11] Figure S2).

The 213 variants included 24 control variants predicted not to contribute substantial risk for neurodevelopmental disorders: six synonymous variants, ten missense variants selected because they were observed in gnomAD population controls,[19] and seven missense variants predicted to be benign from clinical sequencing reported in ClinVar.[18] Across these 24 control variants, GABA uptake was equivalent to the reference (non-variant) version of *SLC6A1* (mean = −0.8%, SD = 24.7%, range = −46.6%–51.0%). We used these variants to define thresholds based on Z scores for severe loss-of-function (≤−82.1%, $p \leq 0.001$, 100 variants), loss-of-function (>−82.1% and ≤−49.2%, $p \leq 0.05$, 25 variants), and gain-of-function (>47.5%, $p \leq 0.05$, 3 variants), with 85 variants in the typical range (>−49.2% to ≤47.5%) (Figure 1B). These four variant effect types are highlighted on a 2D-topology representation of GAT-1 for missense variants (Figure 1A). To see 2D topology of other previously published groups,[11,12] refer to Figure S5.

Limited evidence for gain-of-function variants

Three variants exceeded our gain-of-function threshold of >47.5% (Figure 1). One of these, c.259T>C (p.Phe87Leu), was a control variant selected from population controls in gnomAD. Another, c.144G>T (p.Lys48Asn), was a variant of unknown significance and unknown inheritance that is also present in gnomAD that was identified in an individual with early-onset epileptic encephalopathy.[30] The third one, c.1777A>C (p.Thr593Pro), was a variant of unknown significance absent from gnomAD that was reported in ClinVar without clinical details. Given the caveats of upregulating GAT-1 in HEK293 cells for uptake assays, we sought to assess whether these represented genuine gain-of-function effects. Stable cell lines were generated for the ten variants with the highest GABA uptake values in the UCSF cohort, including p.Phe87Leu and p.Thr593Pro but not p.Lys48Asn. These ten stable cell lines were retested for GABA uptake activity (Figure S6). Across all ten, the uptake values were substantially lower than those observed in the transiently transfected cell lines and exhibited a non-significant trend to being above the WT levels (i.e., 100%). If variants do increase GABA uptake, the effects are small and unlikely to contribute substantially to neurodevelopmental phenotypes.

Neurodevelopmental phenotypes are consistently associated with loss-of-function effects

All 23 PTVs assessed resulted in almost no GABA uptake (i.e., severe loss-of-function), consistent with nonsense-mediated decay or non-functional GAT-1 (Figure 2A). Of 166 clinically ascertained missense variants/in-frame indels, 77 were severe loss-of-function, 27 were loss-of-function, two were gain-of-function, and 60 were in the typical range. Even missense variants/in-frame indels in the typical range had a median GABA uptake of −25.7%, suggesting that a subset of these may also contribute some neurodevelopmental risk via mild loss-of-function (Figure 2A). Across all variants assayed, GABA uptake was correlated with population allele frequency ($R^2 = 0.37$, $p = 8.4 \times 10^{-24}$, Figure S7). Of the 156 variants absent in 114,704 population controls, 115 (73.7%) resulted in severe loss-of-function or loss-of-function. In contrast, none of the variants observed in ten or more individuals showed evidence of reduced GABA uptake (Figure 2B).

Clinical significance has previously been stated for 113 variants (Tables S2 and S5). Where clear determinations were made, these were generally supported by the functional assay: 32 (5 PTVs, 27 missense) were reported as pathogenic or likely pathogenic, and 30 (93.8%) of these were indeed found to be loss-of-function, while 18 missense variants were reported as benign or likely benign of which 15 (83.3%) were in the typical range (Figure 2C). The remaining 163 variants had uncertain ($n = 46$),

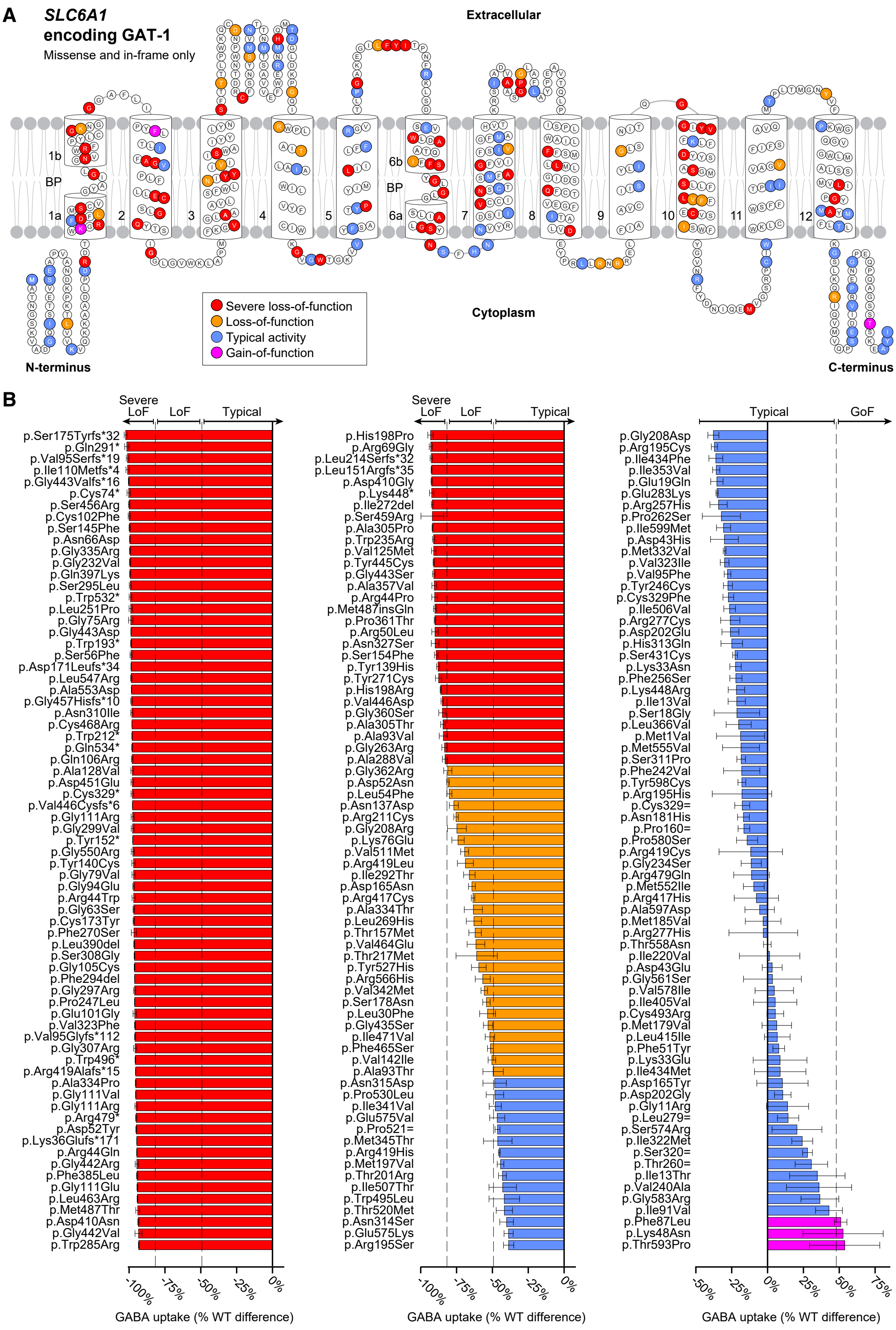

Figure 1. Topology of GAT-1 and GABA uptake values by functional type

(A) 2D representation of GAT-1 organized by 12 TM domains and linking or terminal chains. Only missense and in-frame variants are highlighted.

(B) GABA uptake functional data as a percentage of WT and highlighted by activity type for 213 variants tested across the 599 amino acids of GAT-1. Individual data bars represent mean ± SEM of three biological replicates performed in triplicates. GoF, gain-of-function; LoF, loss-of-function; WT, wildtype.

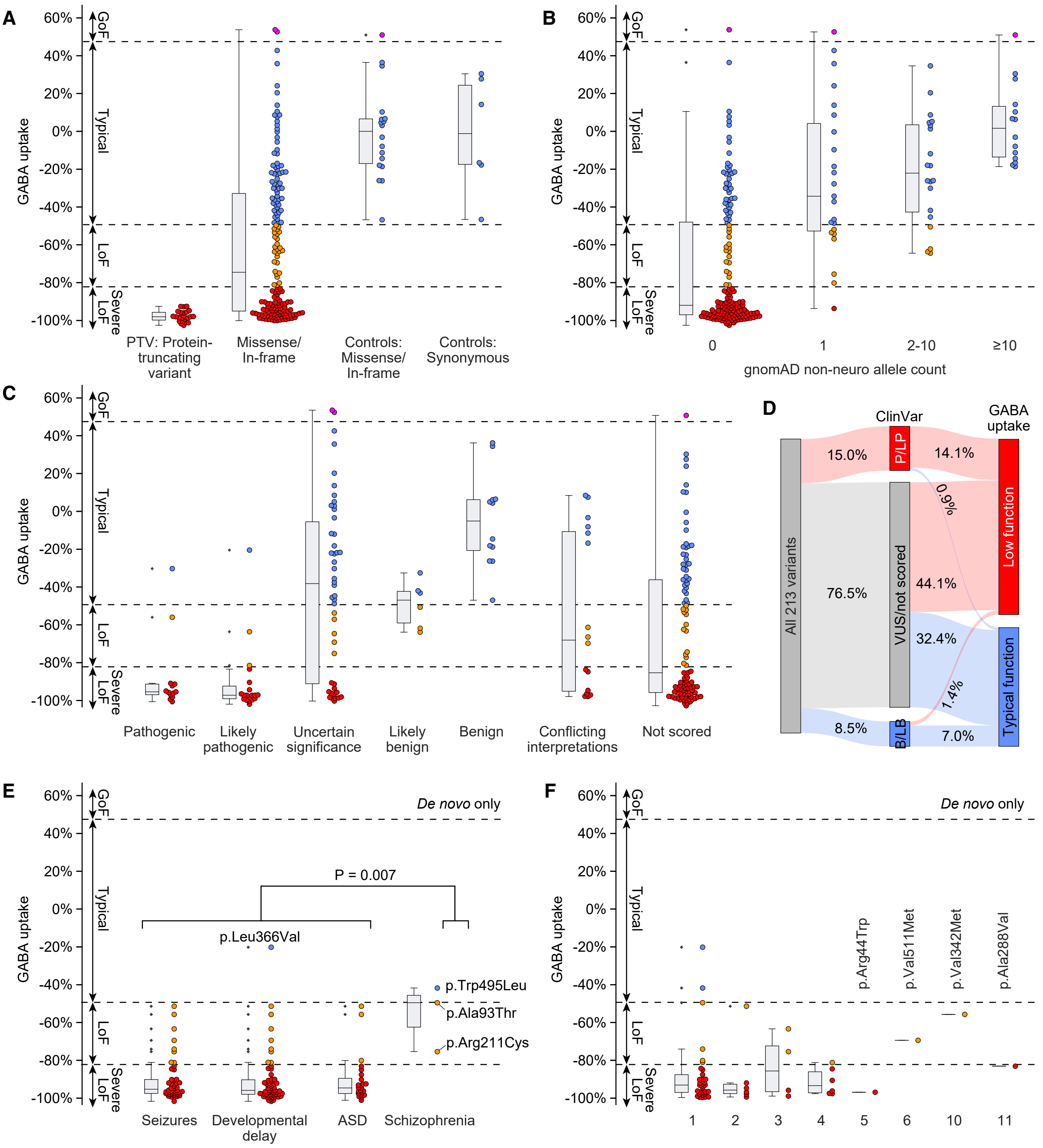

Figure 2. GABA uptake of variants by phenotype and recurrence

(A) GABA uptake data for 213 variants by predicted impact on GAT-1. PTVs and missense/in-frame variants are ascertained from clinical populations (left two categories) while 24 variants selected as controls were split between missense/in-frame and synonymous variants (right two categories) and used to set thresholds for loss of function and gain of function (dashed lines).

(B and C) GABA uptake values by population allele count in 114,704 non-neuropsychiatric samples in gnomAD and by ClinVar clinical significance.

(D) A Sankey plot summarizing the proportion of variants where ClinVar category could be reclassified.

(E) GABA uptake values for *de novo* variants by the presence of seizures, DD, autism spectrum disorder (ASD), or schizophrenia. If phenotypes are comorbid (e.g., seizures and DD) the variant is shown for all phenotypes for which it is reported. All variants are shown in Figure S8B.

(legend continued on next page)

conflicting ($n = 16$), or absent ($n = 101$) clinical significance; 94 (57.7%) of these resulted in severe loss-of-function or loss-of-function. Thus, our functional data are consistent with 97 (45.5%) of the remaining variants being clinically relevant and 71 (33.3%) variants having minimal impact on function (Figure 2D).

To minimize the ascertainment bias, we assessed genotype-phenotype correlations focusing on the 67 *de novo* variants (Figures 2E and S8); all variants are shown in Figure S8B. All but one of these variants were identified in an individual diagnosed with a neurodevelopmental disorder, the exception being c.752T>C (p.Leu251Pro), reported in an individual with no seizures at 21 months of age and no data reported regarding ASD or DD.[20] The completeness of the phenotyping data varied by variant (Tables S1 and S2). Seizures were reported in 46 of the 55 (83.6%) with data, ASD reported in 24 of 44 (54.5%), and DD in 58 of 59 (98.3%). Variants were consistently loss-of-function in individuals with seizures, ASD, and DD (median GABA uptake −0.95, −0.95, and −0.96, respectively). The most frequently reported seizure type was EMAS, reported in 22 of the 37 (59.5%) for whom seizure type was available. In addition, early-onset absence epilepsy (EOAE), childhood absence epilepsy (CAE), developmental and epileptic encephalopathy (DEE), and Lennox-Gastaut syndrome (LGS) were related to reduced GABA uptake. In contrast, non-familial, non-acquired focal epilepsy (NAFE) consistently resulted in typical levels of GABA uptake (Figure S8), suggesting *SLC6A1* disruption is probably not associated with NAFE. No clear patterns were observed between variant effect and age of seizure onset (Figure S9).

Some variants observed in schizophrenia have loss-of-function effects

Two schizophrenia cohorts contributed *SLC6A1* missense variants that were predicted to be damaging (MPC score ≥ 2).[31] The first focused on *de novo* variants from 3,444 individuals and reported that *SLC6A1* was associated with schizophrenia ($p = 7.9 \times 10^{-5}$, uncorrected).[4] Two of the three *de novo* variants resulted in loss-of-function effects (c.277G>A [p.Ala93Thr] and c.631C>T [p.Arg211Cys]), while one was in the typical range (c.1484G>T [p.Trp495Leu]). The median GABA uptake across these variants is −49.5%—a value in the loss-of-function range but higher than that observed for the neurodevelopmental phenotypes ($p = 0.007$, two-sided Wilcoxon test; Figure 2E). The second cohort was a case-control analysis of 24,248 individuals (including the previous 3,444); they reported enrichment for *SLC6A1* variants that did not meet genome-wide correction ($p = 0.006$, uncorrected; $p = 0.50$, corrected).[5] While these additional variants were not reported to be *de novo* in the individuals with schizophrenia, two of the missense variants from this cohort were at the same location as *de novo* variants identified in neurodevelopmental disorders: c.913G>A (p.Ala305Thr) (severe loss-of-function) and c.1000G>A (p.Ala334Thr) (loss-of-function). Of the remaining 11 variants in the second cohort, two resulted in severe loss-of-function (c.1228G>A [p.Asp410Asn] and c.1324G>A [p.Gly442Arg]), two in loss-of-function (c.409A>G [p.Asn137Asp] and c.1249C>T [p.Arg417Cys]), and seven were in the typical range (Table S2; Figure S8).

Correlation of functional data and surface localization of 86 missense variants

Given the role of loss-of-function effects for the clinically relevant *SLC6A1* missense variants, we sought to understand the relationship of protein trafficking mechanisms to these functional outcomes. A reduction in GAT-1 stability could lead to a relative deficit in surface colocalization.[10,12] Surface localization was assessed for 86 missense variants using a high-content imaging approach to assess the relative co-localization of GFP-tagged GAT-1 with a membrane stain. While WT GAT-1 is located on the plasma membrane, it is also distributed at lower levels throughout the cell body in HEK293 cells (Figure S4). Given this mixed localization, the high-content imaging experimental design utilized segmentation as part of the image analysis to quantify the colocalization of GFP tag and plasma membrane stain through the Pearson correlation colocalization (PCC) metric. The WT GAT-1 GFP-tagged plasmid yielded a PCC value of 0.32 ($n = 26$), which we defined as 100% WT surface localization. As a negative control, we used an EV plasmid that contained GFP but not GAT-1; the resulting GFP is distributed non-specifically throughout the cell, with only a small amount on the membrane (Figure S4). The EV plasmid resulted in a PCC value of 0.15 ($n = 26$ replicates), 46.9% below the WT GAT-1 value (Figure S10). For each of the 86 missense variants, we tested six replicates (mean of 5.9 replicates after data cleaning) from which we calculated both the PCC and the percentage WT surface localization (Figure S10). For further analysis, the percentage WT surface localization values below that of EV (46.9%) were increased to 46.9%.

GABA uptake data were correlated with surface localization results ($R^2 = 0.23$, $p = 3 \times 10^{-6}$; Figure 3A). Variants with typical GABA uptake generally had high surface localization, while loss-of-function variants had highly variable surface localization. To interpret these relationships, we applied a K-means algorithm to define three groups of variants ($K = 3$; Figure 3A): group 1 variants present on the surface with typical uptake ($n = 30$), group 2 variants absent from the surface with low uptake ($n = 40$), and group

(F) GABA uptake values of *de novo* variants observed in single individuals (1) or multiple individuals (2–11). Each variant is shown once. The four most recurrent variants (5, 6, 10, and 11) are labeled.
Abbreviations: GoF, gain-of-function; LoF, loss-of-function; A93T, p.Ala93Thr; R211C, p.Arg211Cys; W495L, p.Trp495Leu. Statistical tests: E, two-sided Wilcoxon test. Colors: red, severe LoF; orange, LoF; blue, typical activity; pink, GoF. Boxplot whiskers are based on maximum and mimimum values within 1.5 times the interquartile range.

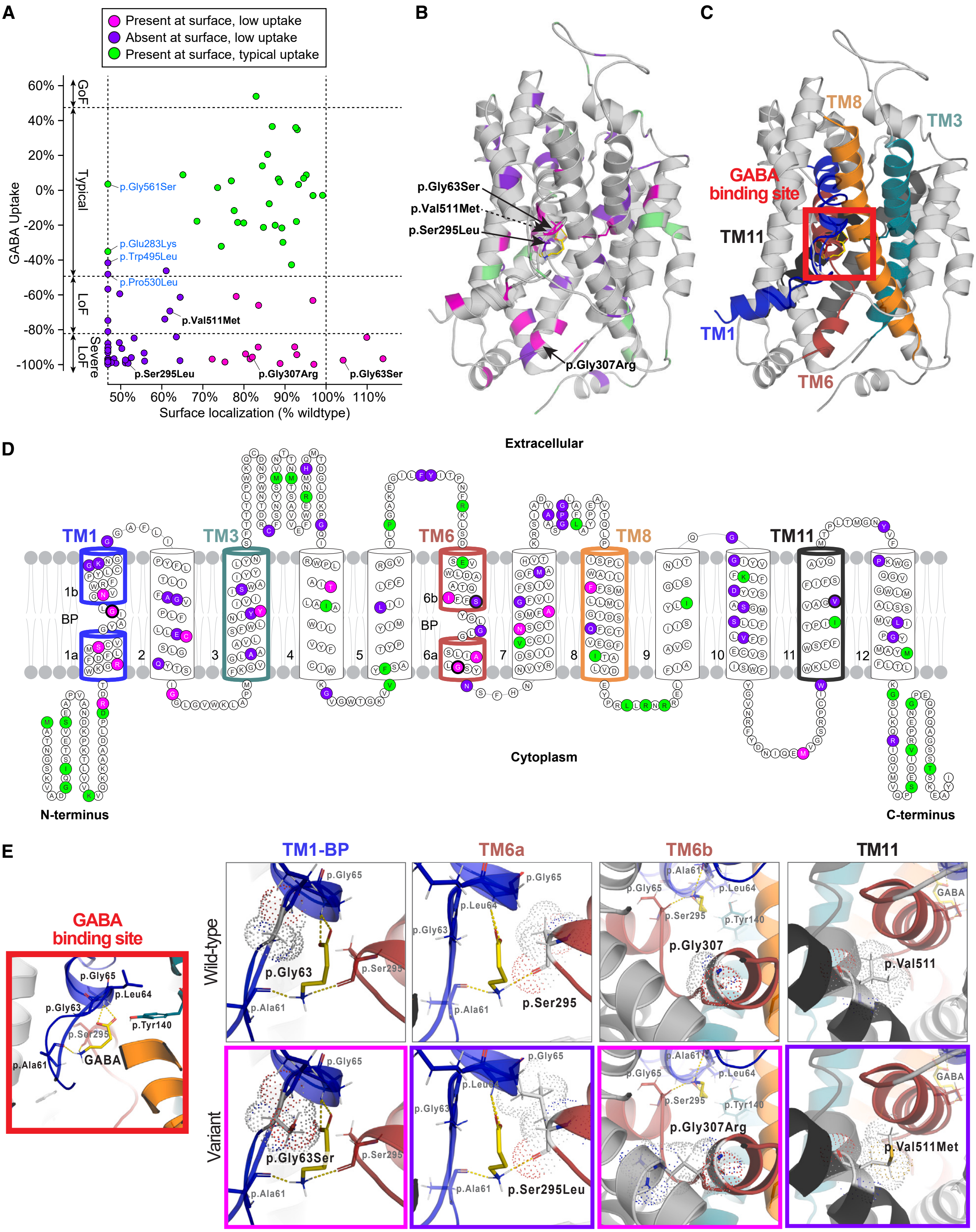

Figure 3. Clustering of surface localization and GABA uptake results of 86 missense variants and highlighted variants in GAT-1 structure and binding site

(A) Individual GABA uptake values (y axis) correlated to surface localization results (x axis) and classified by K-means clustering as either present at the surface and low uptake (magenta), absent at the surface and low uptake (purple), or present at the surface and typical uptake (green). Variants with names in blue denote outliers. All other called variants are further illustrated in (B) and (E).

(B) GAT-1 3D structure in its inward-open conformation with highlighted variants by cluster.

(legend continued on next page)

3 variants that were present on the surface but had low uptake ($n = 16$). Four variants with typical GABA uptake were outliers with very low surface localization: c.1681G>A (p.Gly561Ser) (PCC = 0.11), c.847G>A (p.Glu283Lys) (PCC = 0.12), and c.1589C>T (p.Pro530Leu) (PCC = 0.06) identified in a large-scale epilepsy sequencing project[24] and c.1484G>T (p.Trp495Leu) (PCC = 0.09), a *de novo* variant identified in schizophrenia[4] (Figure S11). In groups 1 and 2, a substantial fraction of the variability in GABA uptake can be explained by relative surface localization ($R^2 = 0.69$, $p = 8 \times 10^{-19}$) but a different mechanism is required to explain the loss-of-function effects of the 16 missense variants in group 3.

Mapping of 86 variants to human GAT-1 structure

The human GAT-1 transporter is a 599 amino acid protein that is a member of the solute carrier 6 (SLC6) transporter family, also known as sodium and chloride (Na^+/Cl^-) coupled transporter proteins. The SLC6 family contains 20 transporter members that are further divided into four subgroups based on substrate selectivity and sequence homology. This includes the neurotransmitter transporters *SLC6A3* (DAT, dopamine transporter) and *SLC6A4* (SERT, serotonin transporter), which share ~50% sequence identity to GAT-1. Like all other SLC6 family members, GAT-1 shares the conserved 12 TM domain conformation with amino (N-) and carboxyl (C-) termini located intracellularly.[32] Previous studies have mapped out important GAT-1 substrate binding site components using the leucine transporter LeuT as a structural model, a bacterial ortholog of the eukaryotic Na^+/Cl^- transporters.[33,34] From this model, the main four TM domains that form the binding pocket, or cylindrical ring, of GAT-1 are identified as TM1, TM3, TM6, and TM8. The most conserved regions across the Na^+/Cl^- transporter family include TM1 and TM6, reflecting their importance in binding substrates and inhibitors.[33] Both TM1 and TM6 helices have unwound "hinge regions" that separate the helices, causing a split of these regions, with the two halves labeled TM1a/TM1b and TM6a/TM6b (Figure 3D). These hinge regions are the formal binding site for GAT-1 substrates and its co-transported ions sodium (2 Na^+) and chloride (Cl^-). More recently, the cryo-EM structure of WT human GAT-1 was determined in the inward-open conformation, giving additional insight into specific binding modalities and GAT-1 mechanics.[17] We mapped all 86 variants to the cryo-EM-derived structure (PDB: 7SK2) and closely analyzed structure-function relationships to determine if this could provide insight into the variants we identified in group 3 (Figures 3B–3D).

Considering the 3D structure (Figures 3B and 3C), we visually calculated the number of variants located on the outer surface of GAT-1 versus the inner surface for each group[35] (Figure S12, Video S1). We distinguished that 80% of group 2 variants (low uptake and poor protein trafficking) and 93.8% of group 3 variants (low uptake but proper protein trafficking) were found buried in the interior of the 3D structure, in contrast to only 36.4% of variants from group 1 (typical uptake and proper protein trafficking). This suggests that variants positioned within the internal structure can have variable impact on protein trafficking but are more likely to lead to reduced function of GAT-1, whereas variants that fall on the outer surface of the protein structure, and more notably the unwound intracellular and extracellular loops (group 1; Figures 3D and S12A), are less likely to cause deficits in protein trafficking or GABA uptake. Additionally, of the 16 variants in group 3, 13 were located within TM domains, and eight of these are found in the TM domains most closely associated with the GABA binding pocket of GAT-1 (TM1, TM3, TM6, and TM8; Figure 3D), strongly suggesting that their loss-of-function effects were through disruption of this binding modality rather than protein trafficking to the membrane.

To assess this hypothesis, variants within the TM domains that form the binding pocket and main intracellular gate (TM1 and TM6, c.187G>A [p.Gly63Ser], c.884C>T [p.Ser295Leu], and c.919G>A [p.Gly307Arg]) were analyzed in more detail considering the 3D structure (Figure 3E). Variant p.Gly63Ser (group 3), a recurrent *de novo* variant seen in two individuals with seizures, severe DD, and ASD, forms part of the TM1 GABA binding site (Figure 3E). The WT residue (p.Gly63) is predicted to form a hydrogen bond with the carboxylic acid of GABA.[17] When altered to p.Gly63Ser, a polar functional group is added, increasing strain on the binding site, which may interfere with the native hydrogen bonds to GABA and reduce uptake. Variant p.Ser295Leu (group 2) also forms part of the GABA binding site but within TM6. In its WT form, p.Ser295 forms hydrogen bonds with the backbone of GABA at the amine ($-NH_2$). The polar to nonpolar p.Ser295Leu variant is likely to weaken this GABA and/or ion binding. Furthermore, the change to the longer functional group in leucine likely causes substantial strain that interferes with the TM1 helix (Figure 3E), potentially impairing protein trafficking and disrupting the opening and closing mechanics of the

(C) GAT-1 3D structure repeated and highlighted by TM domains. The red square indicates the binding site.
(D) 2D topology of GAT-1 structure showing individual variants colored by cluster and corresponding TM domains highlighted from (C).
(E) The GAT-1 binding site inset from (C) (red square) and relevant amino acids is shown with GABA bound. The WT and variant 3D structures of four variants are shown, and the variant insets are boxed in colors relevant to their clustering from (A) (magenta or purple). Two variants form part of the binding site for GABA (p.Gly63Ser and p.Ser295Leu), one variant is part of TM6 (p.Gly307Arg), and the variant in TM11 is the top recurrent variant (p.Val511Met, $n = 6$) with available surface localization data.
Abbreviations: GoF, gain-of-function; LoF, loss-of-function; TM1, TM domain 1; TM3, TM domain 3; TM6, TM domain 6; TM8, TM domain 8; TM11, TM domain 11.

transporter necessary for GABA uptake. This same phenomenon can be seen in the variant p.Gly307Arg (group 3) where changing glycine with the positively charged side chain from arginine creates a large strain and interference between TM6 (red) and TM2 (gray).

Additionally, we examined the most recurrent missense variant that was available within these 86 variants (c.1531G>A [p.Val511Met], group 2, $n = 6$; Table 1) to investigate whether its position outside of the binding pocket (TM11) might suggest an additional role underlying the observed recurrence. Interestingly, the addition of a sulfur group gives rise to one of the most hydrophobic amino acids, methionine, and is positioned within TM11. Logically, we would not expect such a substitution, where both WT and mutant have amino acids with hydrophobic side chains, to impact protein trafficking as we observed. Hydrophobic amino acids are known to interact with hydrophobic ligands such as lipids or the plasma membrane.[36] No clear explanation for recurrence was observed from this analysis.

Taken altogether, our data suggest that variants clustered within the binding pocket of GAT-1 can properly traffic to the membrane but have poor GABA uptake due to a disruption in substrate binding mechanics (group 3). Additionally, those variants that are buried within the inner surfaces of the protein are more likely to impact protein trafficking to the membrane (group 2) over variants found on the outer surface of the protein (group 1), which have minimal impacts on GABA uptake or protein trafficking. As an additional metric, protein stability algorithms deepDDG[37] and DynaMut[38] were performed on GAT-1, and their score outputs were analyzed against our functional data; we did not observe such correlations (Table S2).

Prenatal lethality, gene-level mutability, and dominant-negative effects do not contribute to missense enrichment

Our functional data strongly suggest that loss-of-function effects underlie seizures, DD, and ASD phenotypes, excluding the possibility of undiscovered gain-of-function variants and complex genotype-phenotype relationships as explanations for the enrichment for missense variants observed in *SLC6A1*. We next considered whether PTVs might increase the rate of prenatal lethality leaving a disproportionate number of missense variants in cohorts of children with neurodevelopmental disorders. Adjusting for gene length and DNA sequence, we find the rate of *de novo* PTVs in *SLC6A1* is equivalent to that of other NDD-associated genes (Figure S13A), suggesting it is an excess of *de novo* missense variants rather than a deficit of *de novo* PTVs driving the enrichment. However, the predicted mutability of *SLC6A1* for both missense and PTV variants is equivalent to that of other NDD genes (Figure S13B). As further evidence against prenatal lethality, the diagnostic rates of seizures, DD, and ASD are similar for *de novo* PTVs (92%, 60%, and 100%, respectively) and *de novo* missense (81%, 53%, and 98%, respectively); furthermore, other genes associated with neurodevelopmental delay have a greater impact on early developmental milestones.[39]

We next considered whether missense variants might lead to an ascertainment bias driven by more severe symptoms due to a dominant-negative effect, as observed in *SLC30A2*.[16] We tested this hypothesis by assessing co-transfections of WT and missense variants for two missense variants with typical GABA uptake (c.129C>A [p.Asp43Glu] and c.1302C>G [p.Ile434Met]) and five severe loss-of-function missense variants (p.Gly63Ser, c.419A>G [p.Tyr140Cys], p.Ser295Leu, c.1640T>G [p.Leu547Arg], and c.1648G>A [p.Gly550Arg]). None of the variants showed evidence in keeping with dominant-negative effects via co-transfection and uptake assays (Figure S14).

GAT-1 vulnerability to missense variations explains missense enrichment

The GAT-1 transporter is a highly specific, dynamic, and complicated molecular machine.[17] Could the intricate protein structure render the protein unusually susceptible to loss-of-function missense variation? Exploring this possibility would require extrapolating the GABA uptake results from the 180 assayed missense variants to all possible missense variants for the gene. Multiple algorithms have been developed to estimate the functional impact of missense variants, largely based on conservation across species and constraint in human populations. We used ANNOVar to annotate the 180 missense variants against the output of multiple such algorithms (Table S2) and stepwise linear regression (Table S6) and random forest machine learning (Table S7) to build a predictive model. The R^2 was above 0.55 for three linear regression models independently, ClinPred: $R^2 = 0.58$, $p = 7 \times 10^{-35}$ (Figure 4A),[40] MetaSVM: $R^2 = 0.57$, $p = 8 \times 10^{-34}$,[41] and MetaRNN: $R^2 = 0.55$, $p = 1 \times 10^{-32}$,[42] of note, all three are ensemble models integrating multiple other algorithms trained against large databases, such as ClinVar. Selecting ClinPred and adding additional predictors (MetaSVM,[41] fitCons,[43] phyloP30way mammalian,[44] and LIST-S2[45]) only marginally improved the prediction to $R^2 = 0.62$ probably because of the high correlations between these algorithms (Figure S15). Using a random forest approach to model fitting for the 180 missense variants yielded an $R^2 = 0.55$. Given the risk of overfitting to a relatively small dataset, we elected to use the ClinPred model with linear regression alone (scaled from no predicted impact at 0 to severe predicted impact at 1).

To select comparator genes, we identified nine genes with equivalent evidence of association with ASD (FDR $\leq 1 \times 10^{-10}$) and NDD (FDR $\leq 1 \times 10^{-10}$) that were enriched for PTVs (*de novo* PTV count/*de novo* missense count >2)[2] and had ClinPred scores available. For *SLC6A1* and these nine genes, we used the longest protein-coding isoform from GENCODE v33 to predict all possible missense variants and plotted the ClinPred score distributions

Table 1. **Recurrent *de novo* mutations in *SLC6A1***

SLC6A1 variant	GAT-1 variant	Count	Seizures	EMAS	Absence	Mean seizure onset	Developmental delay	ASD	Schizophrenia	*De novo*	Inherited	GABA uptake	Mutability
c.863C>T	p.Ala288Val	11	yes	yes	yes	25 months	yes	yes	–	yes	yes	−83.0%	0.04
c.1024G>A	p.Val342Met	10	yes	yes	yes	28 months	yes	yes	–	yes	yes	−55.7%	0.04
c.1531G>A	p.Val511Met	6	yes	–	yes	12 months	yes	–	–	yes	yes	−69.4%	0.04
c.130C>T	p.Arg44Trp	5	yes	yes	yes	28 months	yes	–	–	yes	–	−96.8%	0.06
c.331G>A	p.Gly111Arg	4	yes	–	–	–	yes	–	–	yes	–	−97.5%	0.06
c.889G>A	p.Gly297Arg	4	yes	yes	yes	31 months	yes	yes	–	yes	–	−96.1%	0.06
c.913G>A	p.Ala305Thr	4	yes	–	–	–	yes	–	yes	yes	yes	−84.5%	0.04
c.1070C>T	p.Ala357Val	4	yes	yes	yes	17 months	yes	–	–	yes	–	−90.6%	0.04
c.1084G>A	p.Gly362Arg	4	yes	–	yes	48 months	yes	–	–	yes	–	−81.1%	0.06
c.1648G>A	p.Gly550Arg	4	yes	–	–	–	yes	yes	–	yes	–	−97.4%	0.06

Variants were mapped to ENST00000287766.10, GenBank: NM_003042.4. EMAS, epilepsy with myoclonic-atonic seizures; ASD, autism spectrum disorder.

(Figure 4B). In keeping with a higher vulnerability to missense variation, ClinPred scores are substantially higher for *SLC6A1* than for the other nine genes, with a median of 0.76 for *SCL6A1* versus 0.60 for *KDM5B* ($p = 4 \times 10^{-111}$), the next highest gene (Figure 4B). In NDD, we observed a 7-fold enrichment of *de novo* missense variants compared to *de novo* PTVs (43 missense, 6 PTVs, ratio = 7.17).[2] Based on a ClinPred score of ≥0.642, set by the loss-of-function threshold (Figure 4A), we observed the total number of predicted loss-of-function missense sites to be 7-fold higher than the total number of predicted PTV missense sites (2,481 missense, 349 PTVs, ratio = 7.11; Table S7) with a similar ratio after adjusting for mutability (ratio = 7.06), suggesting the magnitude of this missense vulnerability is sufficient to explain the observed missense enrichment. Other measures of missense severity highly correlated to the functional data yield similar patterns (Figure S16).

Using the linear model to convert the ClinPred scores to estimated GABA uptake, we plotted the mean functional impact for each of the 599 amino acids in GAT-1 (Figure 4C). In keeping with the 3D structure results, we see substantial vulnerability throughout TM domains 1 to 10, along with some of the cytoplasmic and extracellular loops. In contrast, the N- and C-terminus loops show limited vulnerability to missense variants, consistent with our directly assayed functional data (Figure 1A).

Recurrent missense variants in *SLC6A1* are at hypermutable sites

GAT-1 vulnerability can account for the enrichment of missense variants in *SLC6A1*; however, this still does not account for the observation of recurrent *de novo* missense mutations (Table 1; Figures 2 and S1). To assess whether mutability may be a factor, we estimated the mutability of all possible missense variants in *SLC6A1* and plotted these estimates against the predicted GABA uptake from ClinPred (Figure 5). A small fraction of missense variants in *SLC6A1* (75 out of 3,943, 1.9%) are at CpG sites that are known to be hypermutable[46] due to the spontaneous deamination of 5-methylcytosine (5mC) to thymine (Figure 5). However, 22 of the 31 recurrent missense variants (71.6%) and all 14 (100%) of the missense variants identified in three or more individuals are in this small hypermutable subset (Figures 5 and S17). The majority of these are at sites we predict to be loss-of-function.

Discussion

SLC6A1 is a promising therapeutic target due to its strong association with neurodevelopmental disorders and seizures and the clear role of encoded GAT-1 in transporting GABA. However, translational progress requires a clear understanding of genotype-phenotype relationships. Some individuals have PTVs, suggesting autosomal dominant loss of function (haploinsufficiency, i.e., insufficient GABA transport) as the mechanism; however, the vast majority of variants are missense. This enrichment of missense variants, combined with multiple recurrent missense mutations, implies a gain-of-function or dominant-negative mechanism (i.e., some missense variants have a greater impact on phenotype than PTVs). The application of a therapy designed to potentiate GABA uptake in a person with a gain-of-function variant could have devastating consequences.

By performing a comprehensive functional screen for *SLC6A1*, we see clear evidence that a reduction in GABA uptake underlies neurodevelopmental and seizure symptoms. Of the 66 germline *de novo* variants assessed, 64 (97.0%) resulted in loss of function (Figure 2E), and the remaining two showed a trend toward reduced GABA uptake (−20.2% for c.1096C>G [p.Leu366Val], −41.7% for c.1484G>T [p.Trp495Leu]). This consistent functional result, alongside the observation that seizures, DD, and

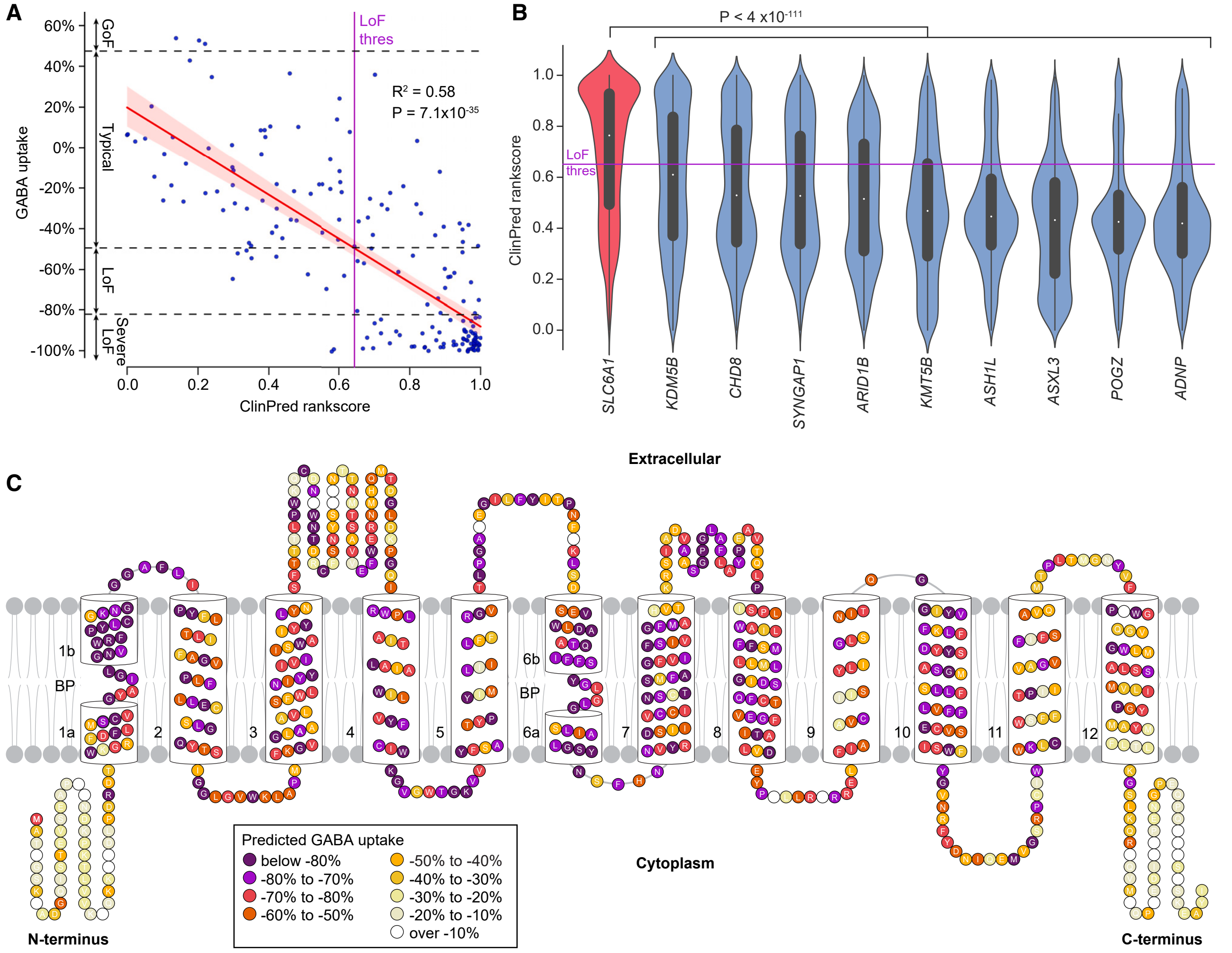

Figure 4. GAT-1 vulnerability underlies the enrichment of *SLC6A1* missense variants
(A) Correlation of individual GABA uptake data and ClinPred rankscore for 180 *SCL6A1*/GAT-1 missense variants. The red line shows the linear regression model with 95% confidence intervals represented by the red shading. The ClinPred value at the estimated loss-of-function threshold is indicated by the purple line (“LoF thres”).
(B) Violin plots represent the ClinPred rankscore distribution for all possible missense variants in *SLC6A1* and nine equivalent PTV-enriched ASD- and NDD-associated genes. The LoF thres line from (A) is also shown.
(C) All 599 amino acids of GAT-1 are colored by the mean predicted GABA uptake of all possible missense variants using the linear regression model from (A) from the annotated ClinPred rankscores.
Abbreviations: GoF, gain-of-function; LoF, loss-of-function. Statistical tests: A, linear regression; B, two-sided Wilcoxon test.

ASD are often co-morbid in the same individual, supports a common mechanism of reduced GABA uptake leading to seizures. Associated seizure types include EMAS, EOAE, CAE, DEE, and LGS but not NAFE (Figure S8). Of note, no association was seen between the age of seizure onset and the degree of GABA uptake impairment (Figure S8), and other metrics of phenotypic severity were not available for assessment. While the results are suggestive that reduced GABA uptake contributes to schizophrenia risk (Figures 2E and S8), further genomic, functional, and longitudinal phenotyping will be required for definitive answers.

In contrast to loss of function, we find limited evidence for phenotypic effects from variants that increase GABA uptake (e.g., gain of function). Nine missense variants were estimated to increase in GABA uptake by ≥ 20% (Figure 1); however, analysis of ten variants using stable transfections, a more accurate method, showed more modest changes, ranging from 8% to 28% over WT (Table S4). While several of these variants that modestly increase GABA uptake are identified in individuals with seizures, none of them are reported to have EMAS, none are classified as pathogenic/likely pathogenic in ClinVar, none are known to be *de novo*, and most are observed in the general population (gnomAD). Based on these data, the neurodevelopmental risk mediated by these variants is probably very small, if any. We considered the possibility that increased GABA uptake might have a protective effect. Genome-wide association studies (GWASs) for epilepsy do not identify significant association at the *SLC6A1* locus[47]; however, the allele frequency of these variants remains below the GWAS detection threshold of ∼2% AF.

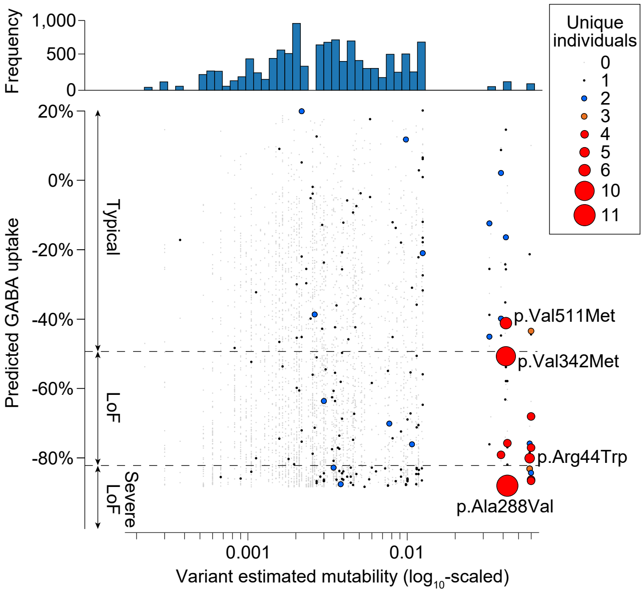

Figure 5. Recurrent missense variants in *SLC6A1* are at hypermutable loci
Estimated mutability based on three base-pair DNA sequences is shown for all 3,952 possible missense variants in *SLC6A1* (x axis). A small number of variants have a ~10-fold higher mutation rate due to the presence of CpG, as shown by the histogram (top). Predicted GABA uptake is predicted for each of these missense variants based on ClinPred (Figure 4). The number of unique individuals is indicated by size and color for each variant; the four most frequent are labeled (Figure 2; Table 1). LoF, loss-of-function.

While the functional data provide a clear answer for the impact on GABA uptake, they do not account for the observed enrichment of missense variants compared with other neurodevelopmental genes; failure to explain this phenomenon could reduce confidence in future therapies aiming to increase GABA uptake. Co-transfection of WT and missense variants did not find evidence of dominant-negative effects (Figure S14), and genomic and phenotype data do not support prenatal lethality from PTVs. In contrast, using computational predictors of missense severity to extrapolate the functional data to all possible missense variants provides a clear answer: *SLC6A1* is simply more sensitive to disruption by missense variants than many genes. This observation also explains the relatively high frequency of *SLC6A1*-related disorders in large cohorts despite the comparatively small size of the gene (599 amino acids). While missense sensitivity can explain the missense enrichment, at face value it does not account for the recurrent missense variants. However, integrating mutability data, we observe that these can be explained by a small number of missense variants that both reduce GABA uptake and occur at highly mutable CpG sites. This missense sensitivity may also explain other genes enriched for missense variants despite predominantly loss-of-function effects, such as *SCN1A* (Dravet syndrome).

Our functional data provide "strong" information to guide clinical interpretation based on the ACMG guidelines (specifically, categories PS3 and BS3). Of the 213 variants we tested, 163 (76.5%) did not have ACMG classifications (e.g., likely pathogenic) in the ClinVar database (Figure 2D). Considering the 127 we identified as loss of function or severe loss of function, only 30 (23.6%) had previously been classified as pathogenic or likely pathogenic with the remainder being unscored (66, 52.0%), uncertain significance (18, 14.2%), conflicting interpretations (10, 7.9%), or likely benign (3, 2.4%). In contrast, of the 86 variants that did not show significant loss of function, only 15 (17.4%) were classified as benign or likely benign with the remainder being unscored (35, 40.7%), uncertain significance (28, 32.6%), conflicting interpretations (6, 7.0%), likely pathogenic (1, 1.2%), or pathogenic (1, 1.2%) (Figure 2D; Table S2).

Overall, for the 60 variants that were scored, only three had opposing clinical and functional interpretations, recorded as likely benign in ClinVar, but observed to be loss-of-function for GABA uptake: c.424G>A (p.Val142Ile) (−50.4% GABA uptake), c.1249C>T (p.Arg417Cys) (−63.5%), and c.1391T>A (p.Val464Glu) (−61.5%). All three variants were submitted to ClinVar by the same submitter without additional evidence for the variant being benign. The associated condition was EMAS/MAE and therefore consistent with *SLC6A1* haploinsufficiency. The clinical interpretation was not supported by the functional data for two further variants: p.Leu366Val (likely pathogenic, −20.2% GABA uptake, not in gnomAD, germline *de novo* in an individual with DD, but no further clinical details) and c.967G>A (p.Val323Ile) (pathogenic, −30.0%, rare in gnomAD [7×10^{-6}], unknown inheritance in an individual with DD, but no further clinical details). We note that our loss-of-function threshold of −49.2% is probably conservative and that milder impairments may be clinically relevant, especially on a sensitized genetic background.

To gain further insight into the functional impact of *SLC6A1* variants causing these phenotypes, we studied whether there were any structure-function relationships. Previous studies have shown that variants in SLC6 family members, including *SLC6A1*, can cause a disruption in protein trafficking to the membrane, and thus impact function and lead to disease.[10,12,48,49] Our study demonstrated a broad clustering of functionally studied variants by whether or not they were detected on the plasma membrane through high-content imaging of GFP fluorescence. We showed that variants clustered in three distinct groups (Figure 3A): group 1 variants present on the cell surface with typical uptake ($n = 30$), group 2 variants absent from the cell surface with low uptake ($n = 40$), and group 3 variants that were present on the cell surface but had low uptake ($n = 16$). Notably, group 3 variants that showed proper protein trafficking and low transporter function were clustered within the binding pocket of GAT-1 and/or the interior of the 3D structure. This suggests that variants in these structural locations are disrupting proper substrate binding and/or transport mechanics (Figures 3D and

3E). Those in group 2 (low uptake and low cell surface localization) shared similar mapping on the 3D structure to group 3, found mainly in positions embedded within the inner surface of the protein and on TM domains (Figures 3D, S12B, and S12D). This shows the importance of those regions, most notably the TM domains, in proper transporter function and perhaps stability and proper folding before trafficking.

Understanding where variants are structurally located and whether they impact trafficking is important for the design of future therapies. For example, before proteins can traffic to their destination, they must properly fold post-translation, and in the presence of variants, this folding can be disrupted. In this case, a therapy that increased the expression of both alleles might have unexpected consequences. Certain SLC6 family members have been previously rescued from folding-deficient and disease-associated variants through the use of pharmacochaperoning.[50] To date, GAT-1 has not been studied enough to understand whether using chaperones could help improve symptoms. Additionally, while we broadly studied the structure-function relationships of a subset of variants, many other pathways influence transport and trafficking mechanisms of GAT-1. This includes N-glycosylation, oligomerization disruption, protein kinase C-mediated phosphorylation, endocytic trafficking, and more.[49,51–55] With the latest advances in GAT-1 structure, new studies need to be conducted to better understand how GAT-1 works in cells mechanistically.

As ever, our experiment has limitations. The functional studies were performed in HEK cells rather than the cell types in the brain that mediate symptoms. Also, we only assayed GABA uptake; gain-of-function consequences could include the uptake of another molecule or an entirely orthogonal function altogether. However, gain of function seems unlikely given the specificity of the transporter to GABA, the functional enrichment of TM domains, the limited evidence of phenotypic consequences of variants in the N- and C-termini, and the observation that missense sensitivity and mutability can account for the missense enrichment. Our dominant-negative assay relies on co-transfection assays that are sensitive to relative plasmid concentrations; studies assessing dimerization directly might reach different conclusions.

Conclusion

In summary, by integrating genomic, functional, and phenotype data, we observe unambiguous evidence that reduced GABA uptake underlies neurodevelopmental sequelae associated with *SLC6A1* variants, including seizures, DD, and ASD. Furthermore, the observed enrichment of missense variants and recurrent missense variants can be explained by the sensitivity of *SLC6A1* to missense variants and mutable CpG sites, respectively. Based on these results, therapeutic strategies for *SLC6A1*-related neurodevelopmental disorders should aim to increase GABA uptake.

Data and code availability

All data used in these analyses is publicly available and reported in the supplemental tables. The code generated during this study is available at https://github.com/sanderslab/slc6a1.git.

Supplemental information

Supplemental information can be found online at https://doi.org/10.1016/j.ajhg.2024.04.021.

Acknowledgments

The BioMarin group wants to thank Amber Freed of SLC6A1Connect, Prof. Dennis Lal, and Prof. Jing-Qiong Kang for reviewing the experimental design. This study incorporates data generated by the DECIPHER community. A full list of centers who contributed to the generation of the data is available from https://deciphergenomics.org/about/stats and via email from contact@deciphergenomics.org. Funding for the DECIPHER project was provided by the Wellcome Trust. We used the Invitae Explorer to explore variants and aggregate testing data from Invitae participants, including genomic and demographic data. We thank Alliance Pharma for their support in developing and running the BLA and GABA mass spectrometry assays.

The UCSF group would also like to thank the SLC6A1 Connect organization and Amber Freed and the American Epilepsy Society (seed grant to S.J.S.) and the National Institute of Mental Health (R01MH116999 and R01MH129751 to S.J.S.) for funding. A special thanks to Cornelius Gati for kindly sharing his GAT-1 structure annotations with us.

Author contributions

A.W. and S.F. designed and supervised the study. M.T. contributed to study design, generated constructs, and executed functional experiments. M.T. and A.W. analyzed the data. A.W. and L.B. compiled the list of variants characterized by BioMarin. W.W. performed cell-based uptake assays, and G.Y.B. designed the mass spectrometry assays. J.H.L. and K.E. conceived the project and contributed to study design. M.T., A.W., and S.F. contributed to manuscript writing.

S.J.S. designed and supervised the study, analyzed data, and wrote the manuscript. K.M.G. supervised the study and revised the manuscript. D.B.S. designed the experimental study, performed transport uptake assays, performed high-content image experiments, analyzed data, and wrote the manuscript. A.L. analyzed the data and wrote the manuscript. C.S.P. assessed the random forest model, and S.D. contributed the mutability analysis. J.L.R. supervised, provided expertise, and analyzed high-content imaging experiments. M.A. provided instrumentation (IN Cell Analyzer 6500 and IN Carta Software). A.S. provided expertise on transporter structure and interpretation of variant impact. K.H. provided insight to variant impact on structure, DynaMut2 results and docked GABA onto the GAT-1 PDB file.

Declaration of interests

D.B.T., G.Y.B., W.W., L.B., J.H.L., S.F., and A.W. are current or past employees of BioMarin Pharmaceutical. S.J.S receives research funding from BioMarin Pharmaceuticals.

Received: September 19, 2023
Accepted: April 26, 2024
Published: May 22, 2024

References

1. Kaplanis, J., Samocha, K.E., Wiel, L., Zhang, Z., Arvai, K.J., Eberhardt, R.Y., Gallone, G., Lelieveld, S.H., Martin, H.C., McRae, J.F., et al. (2020). Evidence for 28 genetic disorders discovered by combining healthcare and research data. Nature *586*, 757–762. https://doi.org/10.1038/s41586-020-2832-5.
2. Fu, J.M., Satterstrom, F.K., Peng, M., Brand, H., Collins, R.L., Dong, S., Wamsley, B., Klei, L., Wang, L., Hao, S.P., et al. (2022). Rare coding variation provides insight into the genetic architecture and phenotypic context of autism. Nat. Genet. *54*, 1320–1331. https://doi.org/10.1038/s41588-022-01104-0.
3. Epi25 Collaborative Electronic address jm4279@cumccolumbiaedu; and Epi25 Collaborative (2021). Sub-genic intolerance, ClinVar, and the epilepsies: A whole-exome sequencing study of 29,165 individuals. Am. J. Hum. Genet. *108*, 965–982. https://doi.org/10.1016/j.ajhg.2021.04.009.
4. Rees, E., Han, J., Morgan, J., Carrera, N., Escott-Price, V., Pocklington, A.J., Duffield, M., Hall, L.S., Legge, S.E., Pardiñas, A.F., et al. (2020). De novo mutations identified by exome sequencing implicate rare missense variants in SLC6A1 in schizophrenia. Nat. Neurosci. *23*, 179–184. https://doi.org/10.1038/s41593-019-0565-2.
5. Singh, T., Poterba, T., Curtis, D., Akil, H., Al Eissa, M., Barchas, J.D., Bass, N., Bigdeli, T.B., Breen, G., Bromet, E.J., et al. (2022). Rare coding variants in ten genes confer substantial risk for schizophrenia. Nature *604*, 509–516. https://doi.org/10.1038/s41586-022-04556-w.
6. López-Rivera, J.A., Pérez-Palma, E., Symonds, J., Lindy, A.S., McKnight, D.A., Leu, C., Zuberi, S., Brunklaus, A., Møller, R.S., and Lal, D. (2020). A catalogue of new incidence estimates of monogenic neurodevelopmental disorders caused by de novo variants. Brain *143*, 1099–1105. https://doi.org/10.1093/brain/awaa051.
7. Bröer, S., and Gether, U. (2012). The solute carrier 6 family of transporters. Br. J. Pharmacol. *167*, 256–278. https://doi.org/10.1111/j.1476-5381.2012.01975.x.
8. Yao, Z., van Velthoven, C.T.J., Nguyen, T.N., Goldy, J., Sedeno-Cortes, A.E., Baftizadeh, F., Bertagnolli, D., Casper, T., Chiang, M., Crichton, K., et al. (2021). A taxonomy of transcriptomic cell types across the isocortex and hippocampal formation. Cell *184*, 3222–3241.e26. https://doi.org/10.1016/j.cell.2021.04.021.
9. Kang, H.J., Kawasawa, Y.I., Cheng, F., Zhu, Y., Xu, X., Li, M., Sousa, A.M.M., Pletikos, M., Meyer, K.A., Sedmak, G., et al. (2011). Spatio-temporal transcriptome of the human brain. Nature *478*, 483–489. https://doi.org/10.1038/nature10523.
10. Cai, K., Wang, J., Eissman, J., Wang, J., Nwosu, G., Shen, W., Liang, H.-C., Li, X.-J., Zhu, H.-X., Yi, Y.-H., et al. (2019). A missense mutation in SLC6A1 associated with Lennox-Gastaut syndrome impairs GABA transporter 1 protein trafficking and function. Exp. Neurol. *320*, 112973. https://doi.org/10.1016/j.expneurol.2019.112973.
11. Mattison, K.A., Butler, K.M., Inglis, G.A.S., Dayan, O., Boussidan, H., Bhambhani, V., Philbrook, B., da Silva, C., Alexander, J.J., Kanner, B.I., and Escayg, A. (2018). SLC6A1 variants identified in epilepsy patients reduce γ-aminobutyric acid transport. Epilepsia *59*, e135–e141. https://doi.org/10.1111/epi.14531.
12. Mermer, F., Poliquin, S., Rigsby, K., Rastogi, A., Shen, W., Romero-Morales, A., Nwosu, G., McGrath, P., Demerast, S., Aoto, J., et al. (2021). Common molecular mechanisms of SLC6A1 variant-mediated neurodevelopmental disorders in astrocytes and neurons. Brain *144*, 2499–2512. https://doi.org/10.1093/brain/awab207.
13. Poliquin, S., Hughes, I., Shen, W., Mermer, F., Wang, J., Mack, T., Xu, D., and Kang, J.-Q. (2021). Genetic mosaicism, intrafamilial phenotypic heterogeneity, and molecular defects of a novel missense SLC6A1 mutation associated with epilepsy and ADHD. Exp. Neurol. *342*, 113723. https://doi.org/10.1016/j.expneurol.2021.113723.
14. Wang, J., Poliquin, S., Mermer, F., Eissman, J., Delpire, E., Wang, J., Shen, W., Cai, K., Li, B.-M., Li, Z.-Y., et al. (2020). Endoplasmic reticulum retention and degradation of a mutation in SLC6A1 associated with epilepsy and autism. Mol. Brain *13*, 76. https://doi.org/10.1186/s13041-020-00612-6.
15. Ben-Shalom, R., Keeshen, C.M., Berrios, K.N., An, J.Y., Sanders, S.J., and Bender, K.J. (2017). Opposing Effects on NaV1.2 Function Underlie Differences Between SCN2A Variants Observed in Individuals With Autism Spectrum Disorder or Infantile Seizures. Biol. Psychiatry *82*, 224–232. https://doi.org/10.1016/j.biopsych.2017.01.009.
16. Lasry, I., Seo, Y.A., Ityel, H., Shalva, N., Pode-Shakked, B., Glaser, F., Berman, B., Berezovsky, I., Goncearenco, A., Klar, A., et al. (2012). A dominant negative heterozygous G87R mutation in the zinc transporter, ZnT-2 (SLC30A2), results in transient neonatal zinc deficiency. J. Biol. Chem. *287*, 29348–29361. https://doi.org/10.1074/jbc.M112.368159.
17. Motiwala, Z., Aduri, N.G., Shaye, H., Han, G.W., Lam, J.H., Katritch, V., Cherezov, V., and Gati, C. (2022). Structural basis of GABA reuptake inhibition. Nature *606*, 820–826. https://doi.org/10.1038/s41586-022-04814-x.
18. Landrum, M.J., Lee, J.M., Benson, M., Brown, G.R., Chao, C., Chitipiralla, S., Gu, B., Hart, J., Hoffman, D., Jang, W., et al. (2018). ClinVar: improving access to variant interpretations and supporting evidence. Nucleic Acids Res. *46*, D1062–D1067. https://doi.org/10.1093/nar/gkx1153.
19. Karczewski, K.J., Francioli, L.C., Tiao, G., Cummings, B.B., Alföldi, J., Wang, Q., Collins, R.L., Laricchia, K.M., Ganna, A., Birnbaum, D.P., et al. (2020). The mutational constraint spectrum quantified from variation in 141,456 humans. Nature *581*, 434–443. https://doi.org/10.1038/s41586-020-2308-7.
20. Goodspeed, K., Pérez-Palma, E., Iqbal, S., Cooper, D., Scimemi, A., Johannesen, K.M., Stefanski, A., Demarest, S., Helbig, K.L., Kang, J., et al. (2020). Current knowledge of SLC6A1-related neurodevelopmental disorders. Brain Commun. *2*, fcaa170. https://doi.org/10.1093/braincomms/fcaa170.
21. Wang, K., Li, M., and Hakonarson, H. (2010). ANNOVAR: functional annotation of genetic variants from high-throughput sequencing data. Nucleic Acids Res. *38*, e164. https://doi.org/10.1093/nar/gkq603.
22. Friesner, R.A., Murphy, R.B., Repasky, M.P., Frye, L.L., Greenwood, J.R., Halgren, T.A., Sanschagrin, P.C., and Mainz, D.T. (2006). Extra precision glide: docking and scoring incorporating a model of hydrophobic enclosure for protein-ligand complexes. J. Med. Chem. *49*, 6177–6196. https://doi.org/10.1021/jm051256o.
23. Carvill, G.L., McMahon, J.M., Schneider, A., Zemel, M., Myers, C.T., Saykally, J., Nguyen, J., Robbiano, A., Zara, F., Specchio,

N., et al. (2015). Mutations in the GABA Transporter SLC6A1 Cause Epilepsy with Myoclonic-Atonic Seizures. Am. J. Hum. Genet. *96*, 808–815. https://doi.org/10.1016/j.ajhg.2015.02.016.

24. Epi25 Collaborative Electronic address sberkovic@unimelbeduau; and Epi25 Collaborative (2019). Ultra-Rare Genetic Variation in the Epilepsies: A Whole-Exome Sequencing Study of 17,606 Individuals. Am. J. Hum. Genet. *105*, 267–282. https://doi.org/10.1016/j.ajhg.2019.05.020.
25. Firth, H.V., Richards, S.M., Bevan, A.P., Clayton, S., Corpas, M., Rajan, D., Van Vooren, S., Moreau, Y., Pettett, R.M., and Carter, N.P. (2009). DECIPHER: Database of Chromosomal Imbalance and Phenotype in Humans Using Ensembl Resources. Am. J. Hum. Genet. *84*, 524–533. https://doi.org/10.1016/j.ajhg.2009.03.010.
26. Heyne, H.O., Singh, T., Stamberger, H., Abou Jamra, R., Caglayan, H., Craiu, D., De Jonghe, P., Guerrini, R., Helbig, K.L., Koeleman, B.P.C., et al. (2018). De novo variants in neurodevelopmental disorders with epilepsy. Nat. Genet. *50*, 1048–1053. https://doi.org/10.1038/s41588-018-0143-7.
27. Johannesen, K.M., Gardella, E., Linnankivi, T., Courage, C., de Saint Martin, A., Lehesjoki, A.-E., Mignot, C., Afenjar, A., Lesca, G., Abi-Warde, M.-T., et al. (2018). Defining the phenotypic spectrum of SLC6A1 mutations. Epilepsia *59*, 389–402. https://doi.org/10.1111/epi.13986.
28. Satterstrom, F.K., Kosmicki, J.A., Wang, J., Breen, M.S., De Rubeis, S., An, J.-Y., Peng, M., Collins, R., Grove, J., Klei, L., et al. (2020). Large-Scale Exome Sequencing Study Implicates Both Developmental and Functional Changes in the Neurobiology of Autism. Cell *180*, 568–584.e23. https://doi.org/10.1016/j.cell.2019.12.036.
29. Symonds, J.D., Zuberi, S.M., Stewart, K., McLellan, A., O'Regan, M., MacLeod, S., Jollands, A., Joss, S., Kirkpatrick, M., Brunklaus, A., et al. (2019). Incidence and phenotypes of childhood-onset genetic epilepsies: a prospective population-based national cohort. Brain *142*, 2303–2318. https://doi.org/10.1093/brain/awz195.
30. Truty, R., Patil, N., Sankar, R., Sullivan, J., Millichap, J., Carvill, G., Entezam, A., Esplin, E.D., Fuller, A., Hogue, M., et al. (2019). Possible precision medicine implications from genetic testing using combined detection of sequence and intragenic copy number variants in a large cohort with childhood epilepsy. Epilepsia Open *4*, 397–408. https://doi.org/10.1002/epi4.12348.
31. Samocha, K.E., Kosmicki, J.A., Karczewski, K.J., O'Donnell-Luria, A.H., Pierce-Hoffman, E., MacArthur, D.G., Neale, B.M., and Daly, M.J. (2017). Regional missense constraint improves variant deleteriousness prediction. Preprint at bioRxiv. https://doi.org/10.1101/148353.
32. Pramod, A.B., Foster, J., Carvelli, L., and Henry, L.K. (2013). SLC6 transporters: structure, function, regulation, disease association and therapeutics. Mol. Aspects Med. *34*, 197–219. https://doi.org/10.1016/j.mam.2012.07.002.
33. Yamashita, A., Singh, S.K., Kawate, T., Jin, Y., and Gouaux, E. (2005). Crystal structure of a bacterial homologue of Na+/Cl–dependent neurotransmitter transporters. Nature *437*, 215–223. https://doi.org/10.1038/nature03978.
34. Gotfryd, K., Boesen, T., Mortensen, J.S., Khelashvili, G., Quick, M., Terry, D.S., Missel, J.W., LeVine, M.V., Gourdon, P., Blanchard, S.C., et al. (2020). X-ray structure of LeuT in an inward-facing occluded conformation reveals mechanism of substrate release. Nat. Commun. *11*, 1005. https://doi.org/10.1038/s41467-020-14735-w.
35. Stitziel, N.O., Tseng, Y.Y., Pervouchine, D., Goddeau, D., Kasif, S., and Liang, J. (2003). Structural Location of Disease-associated Single-nucleotide Polymorphisms. J. Mol. Biol. *327*, 1021–1030. https://doi.org/10.1016/S0022-2836(03)00240-7.
36. Betts, M.J., and Russell, R.B. (2003). Amino acid properties and conseqeunces of substitutions. In Bioinformatics for Geneticits, M.R. Barnes and I.c. Gray, eds.
37. Cao, H., Wang, J., He, L., Qi, Y., and Zhang, J.Z. (2019). DeepDDG: Predicting the Stability Change of Protein Point Mutations Using Neural Networks. J. Chem. Inf. Model. *59*, 1508–1514. https://doi.org/10.1021/acs.jcim.8b00697.
38. Rodrigues, C.H., Pires, D.E., and Ascher, D.B. (2018). DynaMut: predicting the impact of mutations on protein conformation, flexibility and stability. Nucleic Acids Res. *46*, W350–W355. https://doi.org/10.1093/nar/gky300.
39. Wickstrom, J., Farmer, C., Green Snyder, L., Mitz, A.R., Sanders, S.J., Bishop, S., and Thurm, A. (2021). Patterns of delay in early gross motor and expressive language milestone attainment in probands with genetic conditions versus idiopathic ASD from SFARI registries. J. Child Psychol. Psychiatry *62*, 1297–1307. https://doi.org/10.1111/jcpp.13492.
40. Alirezaie, N., Kernohan, K.D., Hartley, T., Majewski, J., and Hocking, T.D. (2018). ClinPred: Prediction Tool to Identify Disease-Relevant Nonsynonymous Single-Nucleotide Variants. Am. J. Hum. Genet. *103*, 474–483. https://doi.org/10.1016/j.ajhg.2018.08.005.
41. Kim, S., Jhong, J.-H., Lee, J., and Koo, J.-Y. (2017). Meta-analytic support vector machine for integrating multiple omics data. BioData Min. *10*, 2. https://doi.org/10.1186/s13040-017-0126-8.
42. Li, C., Zhi, D., Wang, K., and Liu, X. (2022). MetaRNN: differentiating rare pathogenic and rare benign missense SNVs and InDels using deep learning. Genome Med. *14*, 115. https://doi.org/10.1186/s13073-022-01120-z.
43. Gulko, B., Hubisz, M.J., Gronau, I., and Siepel, A. (2015). A method for calculating probabilities of fitness consequences for point mutations across the human genome. Nat. Genet. *47*, 276–283. https://doi.org/10.1038/ng.3196.
44. Pollard, K.S., Hubisz, M.J., Rosenbloom, K.R., and Siepel, A. (2010). Detection of nonneutral substitution rates on mammalian phylogenies. Genome Res. *20*, 110–121. https://doi.org/10.1101/gr.097857.109.
45. Malhis, N., Jacobson, M., Jones, S.J.M., and Gsponer, J. (2020). LIST-S2: taxonomy based sorting of deleterious missense mutations across species. Nucleic Acids Res. *48*, W154–W161. https://doi.org/10.1093/nar/gkaa288.
46. Lynch, M. (2010). Rate, molecular spectrum, and consequences of human mutation. Proc. Natl. Acad. Sci. USA *107*, 961–968. https://doi.org/10.1073/pnas.0912629107.
47. International League Against Epilepsy Consortium on Complex Epilepsies, Stevelink, R., Campbell, C., Chen, S., Abou-Khalil, B., Adesoji, O.M., Afawi, Z., Amadori, E., Anderson, A., Anderson, J., et al. (2023). GWAS meta-analysis of over 29,000 people with epilepsy identifies 26 risk loci and subtype-specific genetic architecture. Nat. Genet. *55*, 1471–1482. https://doi.org/10.1038/s41588-023-01485-w.
48. Kristensen, A.S., Andersen, J., Jørgensen, T.N., Sørensen, L., Eriksen, J., Loland, C.J., Strømgaard, K., and Gether, U.

(2011). SLC6 neurotransmitter transporters: structure, function, and regulation. Pharmacol. Rev. *63*, 585–640. https://doi.org/10.1124/pr.108.000869.

49. Cai, G., Salonikidis, P.S., Fei, J., Schwarz, W., Schülein, R., Reutter, W., and Fan, H. (2005). The role of N-glycosylation in the stability, trafficking and GABA-uptake of GABA-transporter 1: Terminal N-glycans facilitate efficient GABA-uptake activity of the GABA transporter. FEBS J. *272*, 1625–1638. https://doi.org/10.1111/j.1742-4658.2005.04595.x.
50. Freissmuth, M., Stockner, T., and Sucic, S. (2018). SLC6 Transporter Folding Diseases and Pharmacochaperoning. Handb. Exp. Pharmacol. *245*, 249–270. https://doi.org/10.1007/164_2017_71.
51. Chen, N.-H., Reith, M.E.A., and Quick, M.W. (2004). Synaptic uptake and beyond: the sodium- and chloride-dependent neurotransmitter transporter family SLC6. Pflugers Arch. *447*, 519–531. https://doi.org/10.1007/s00424-003-1064-5.
52. Whitworth, T.L., and Quick, M.W. (2001). Substrate-induced Regulation of γ-Aminobutyric Acid Transporter Trafficking Requires Tyrosine Phosphorylation. J. Biol. Chem. *276*, 42932–42937. https://doi.org/10.1074/jbc.M107638200.
53. Wang, D., and Quick, M.W. (2005). Trafficking of the Plasma Membrane γ-Aminobutyric Acid Transporter GAT1. J. Biol. Chem. *280*, 18703–18709. https://doi.org/10.1074/jbc.M500381200.
54. Scholze, P., Freissmuth, M., and Sitte, H.H. (2002). Mutations within an Intramembrane Leucine Heptad Repeat Disrupt Oligomer Formation of the Rat GABA Transporter 1. J. Biol. Chem. *277*, 43682–43690. https://doi.org/10.1074/jbc.M205602200.
55. Cristóvão-Ferreira, S., Vaz, S.H., Ribeiro, J.A., and Sebastião, A.M. (2009). Adenosine A2A receptors enhance GABA transport into nerve terminals by restraining PKC inhibition of GAT-1. J. Neurochem. *109*, 336–347. https://doi.org/10.1111/j.1471-4159.2009.05963.x.

The American Journal of Human Genetics, Volume *111*

Supplemental information

Haploinsufficiency underlies the neurodevelopmental consequences of *SLC6A1* variants

Dina Buitrago Silva, Marena Trinidad, Alicia Ljungdahl, Jezrael L. Revalde, Geoffrey Y. Berguig, William Wallace, Cory S. Patrick, Lorenzo Bomba, Michelle Arkin, Shan Dong, Karol Estrada, Keino Hutchinson, Jonathan H. LeBowitz, Avner Schlessinger, Katrine M. Johannesen, Rikke S. Møller, Kathleen M. Giacomini, Steven Froelich, Stephan J. Sanders, and Arthur Wuster

Supplemental Figures

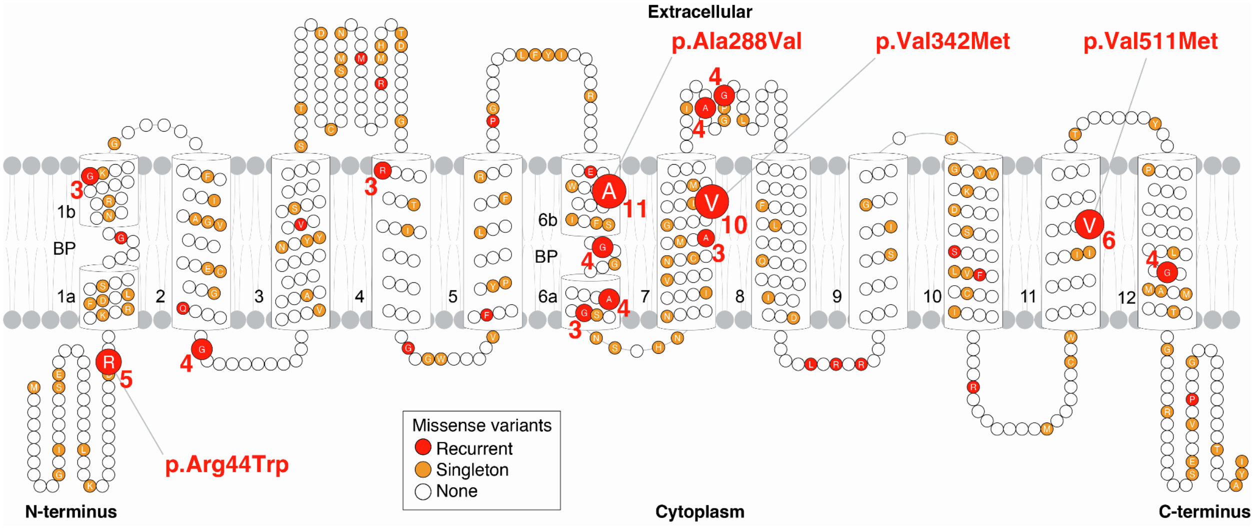

Figure S1. Recurrent missense variants in *SLC6A1*. 2D representation of the 599 amino acids in the GAT-1 protein showing singleton (orange) and recurrent (red) missense variants. Missense variants identified in three or more individuals are represented by the size of the amino acid and the number next to it (Table S2).

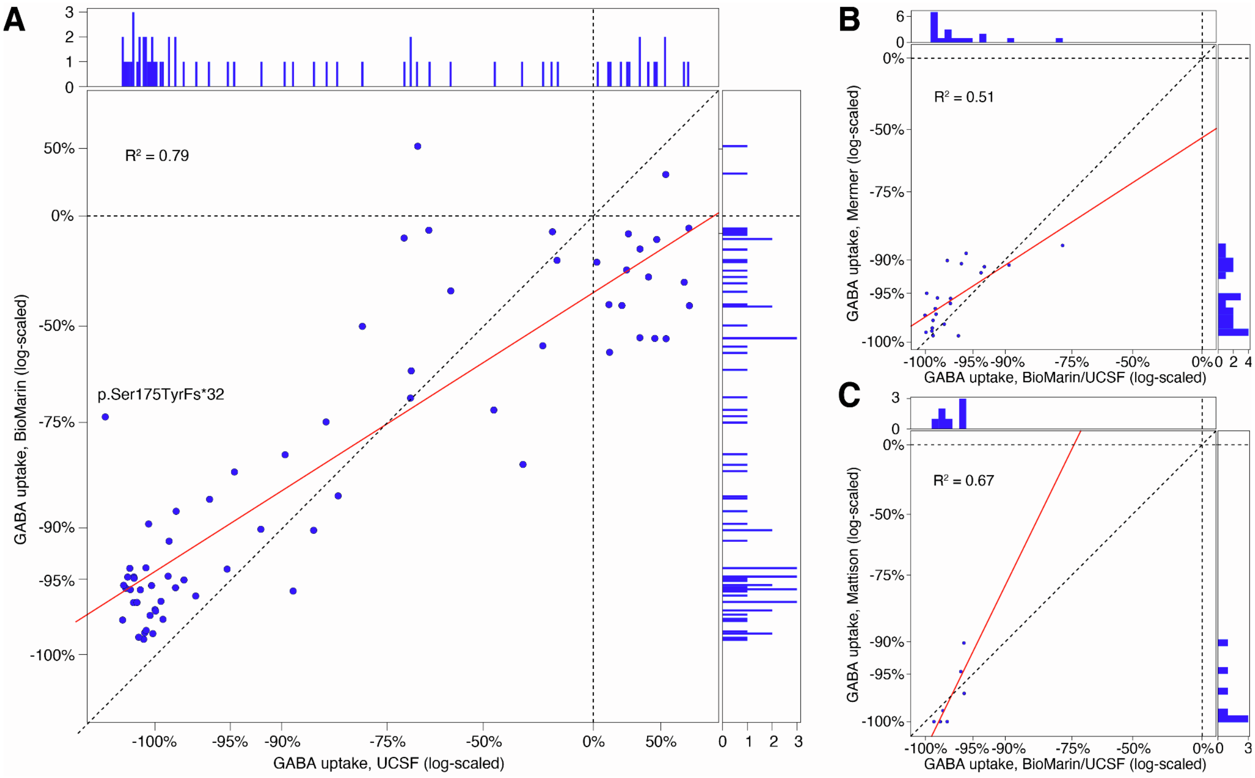

Figure S2. Correlation between GABA uptake data across datasets. A) GABA uptake functional data as a percentage of wildtype generated for 68 *SLC6A1* variants assayed by both the UCSF group (x-axis) and the BioMarin group (y-axis). The red line shows the linear regression model (R^2 =0.79). **B)** GABA uptake data for 21 variants assayed by UCSF/BioMarin (x-axis) and Mermer group[1] (y-axis). **C)** GABA uptake data for 7 variants assayed by UCSF/BioMarin (x-axis) and Mattison group[2] (y-axis).

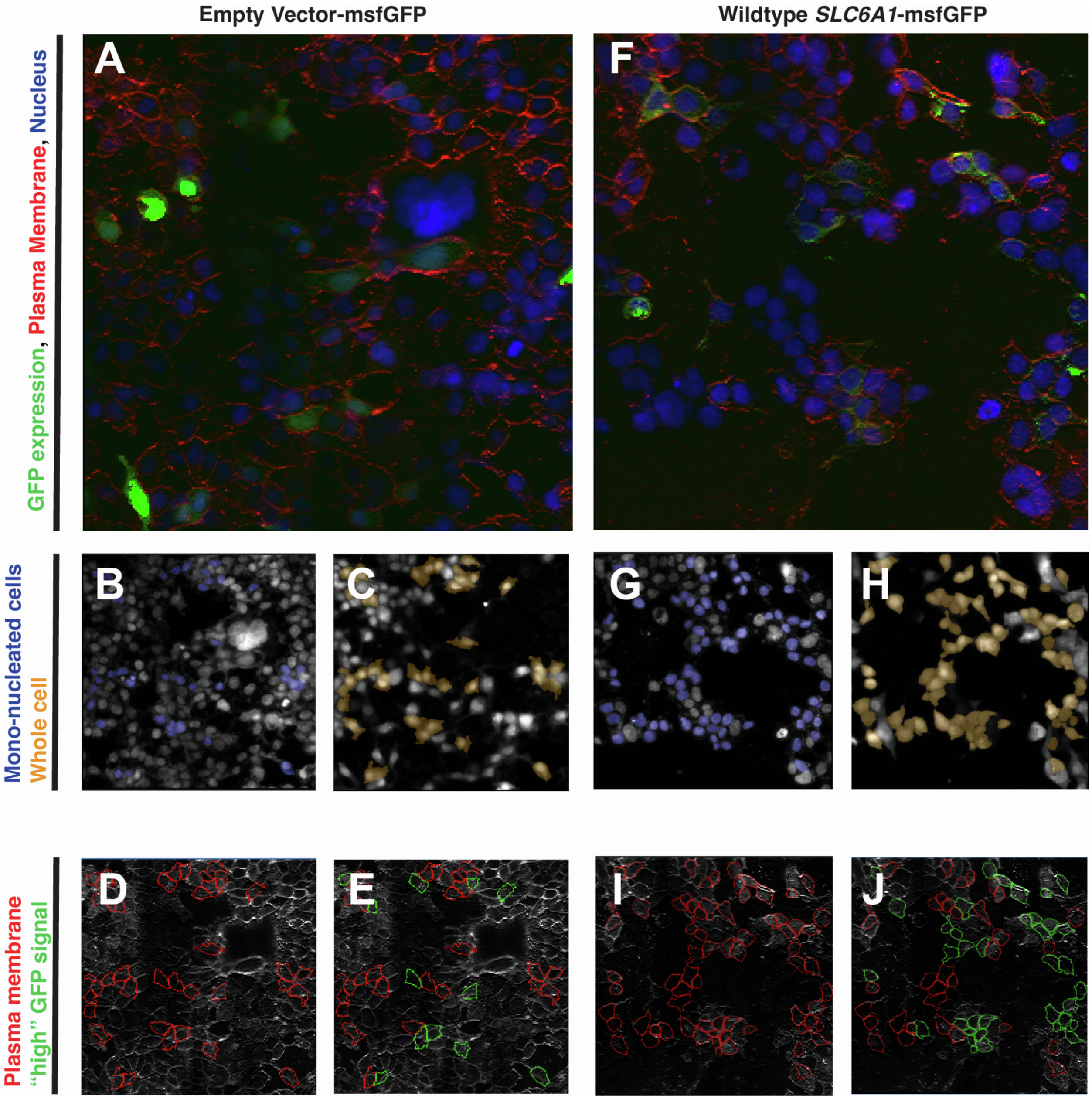

Figure S3: High content imaging colocalization and segmentation. A) HEK293 cells transfected with GFP-tagged empty vector shown with colocalized fluorescence tags. IN Carta image analysis was used to identify **B)** mono-nucleated cells, **C)** whole cells, **D)** the plasma membrane, and **E)** plasma membrane in cells with "high" GFP signal. These panels are repeated for HEK293 cells transfected with GFP-tagged wildtype *SLC6A1* vector shown with colocalized fluorescence tags in **F, G, H, I,** and **J**.

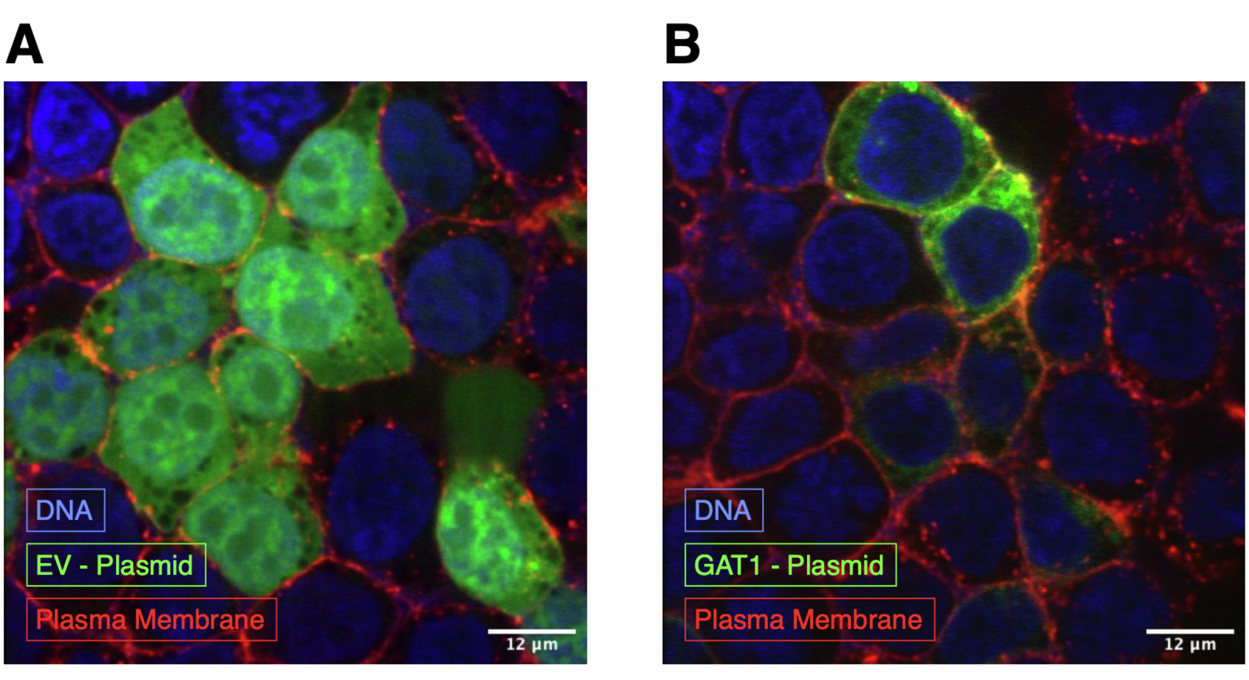

Figure S4. Confocal imaging of HEK293 cells. Transient transfected HEK293 cells overexpressing **A)** empty vector GFP (EV), or **B)** GAT-1 GFP plasmid.

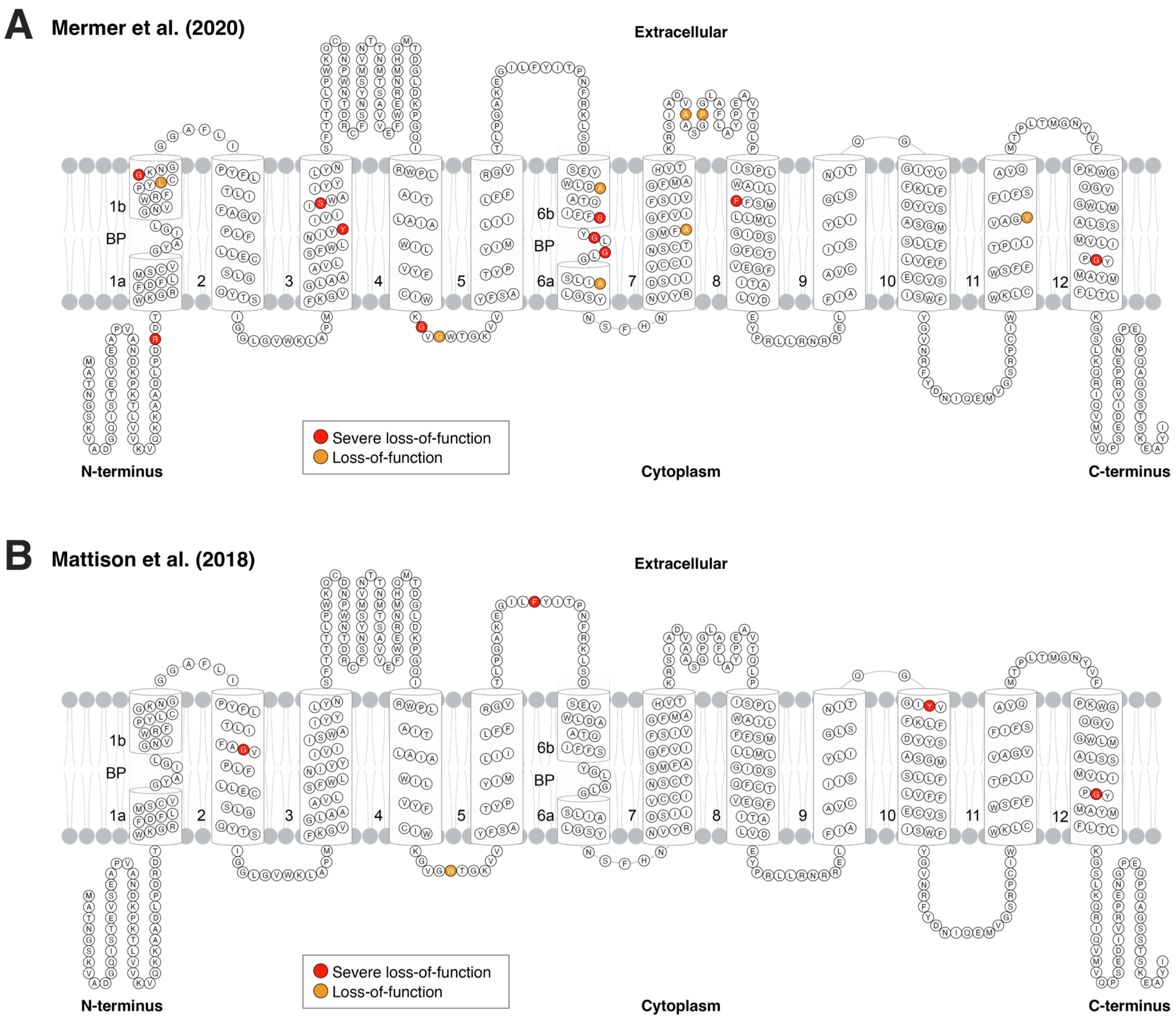

Figure S5. Topology of the GAT-1 protein and GABA uptake values of previous datasets. 2D representation of the GAT-1 protein showing functional results for missense and in-frame variants from: **A)** Mermer group[1] and **B)** Mattison group[2].

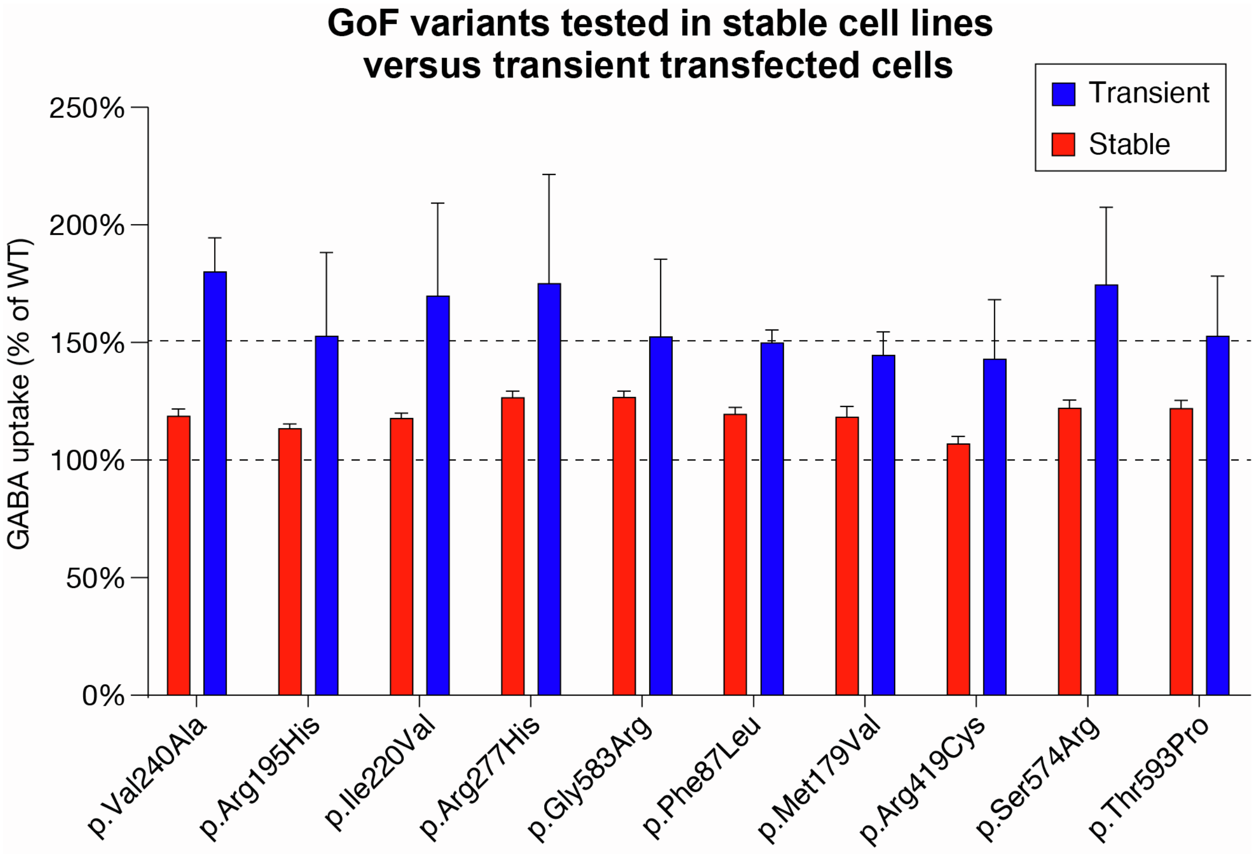

Figure S6: GABA uptake assay of potential gain of function variants in transient transfected cells versus stable expressing HEK293 cells. These results (**Table S4**) are based on the highest functioning variants from UCSF data (blue bars, transient) and replicated in stable cell lines (red bars). Results are shown as a percentage of wildtype (y-axis) and error bars represent mean ± SEM of 3 biological replicates.

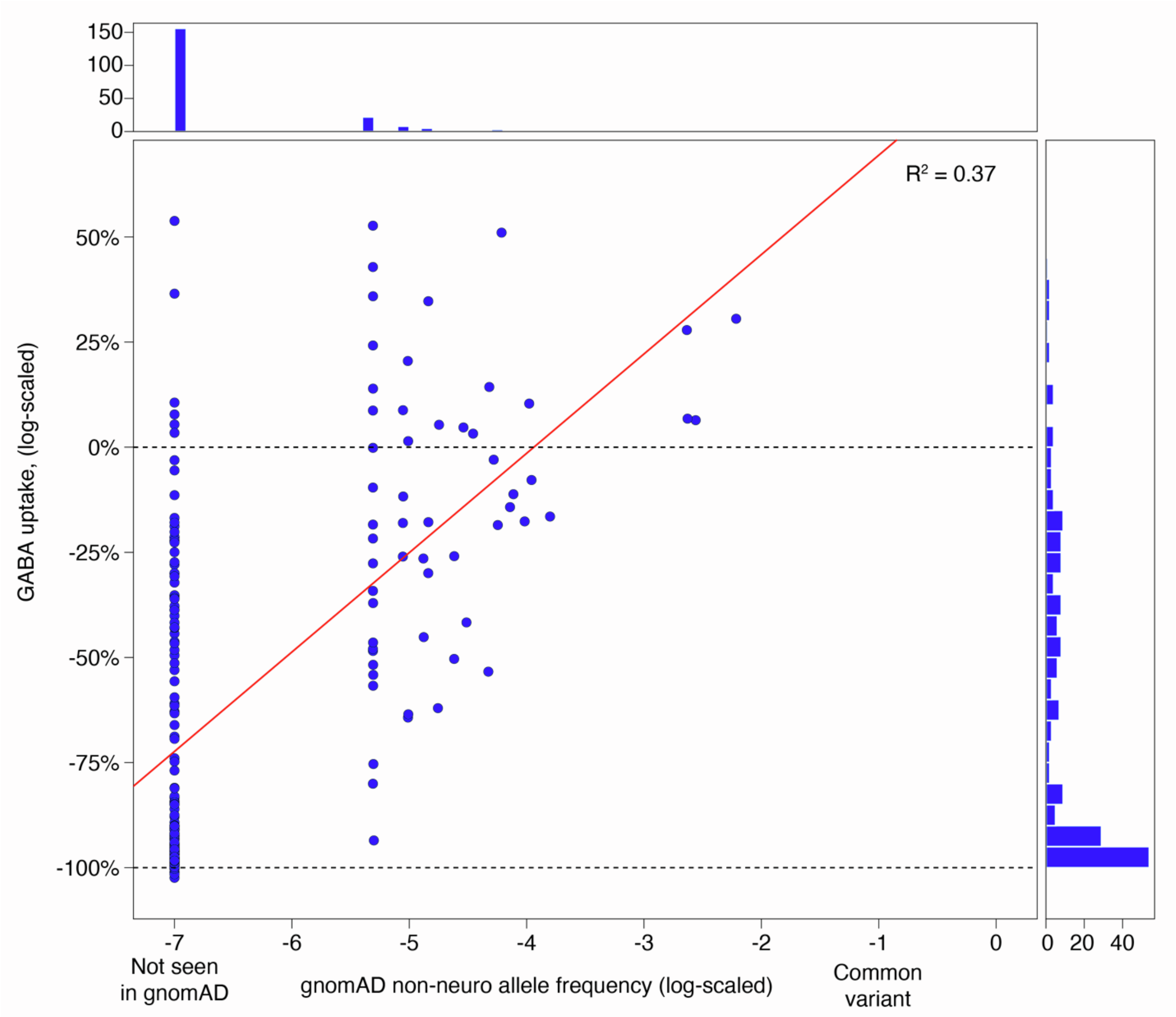

Figure S7. Correlation between GABA uptake and population allele frequency. Relationship between GABA uptake functional data as a percentage of wildtype (y-axis) and population allele frequency (x-axis) based on gnomAD (v2 non-neuro) for all 213 *SLC6A1* variants assayed.

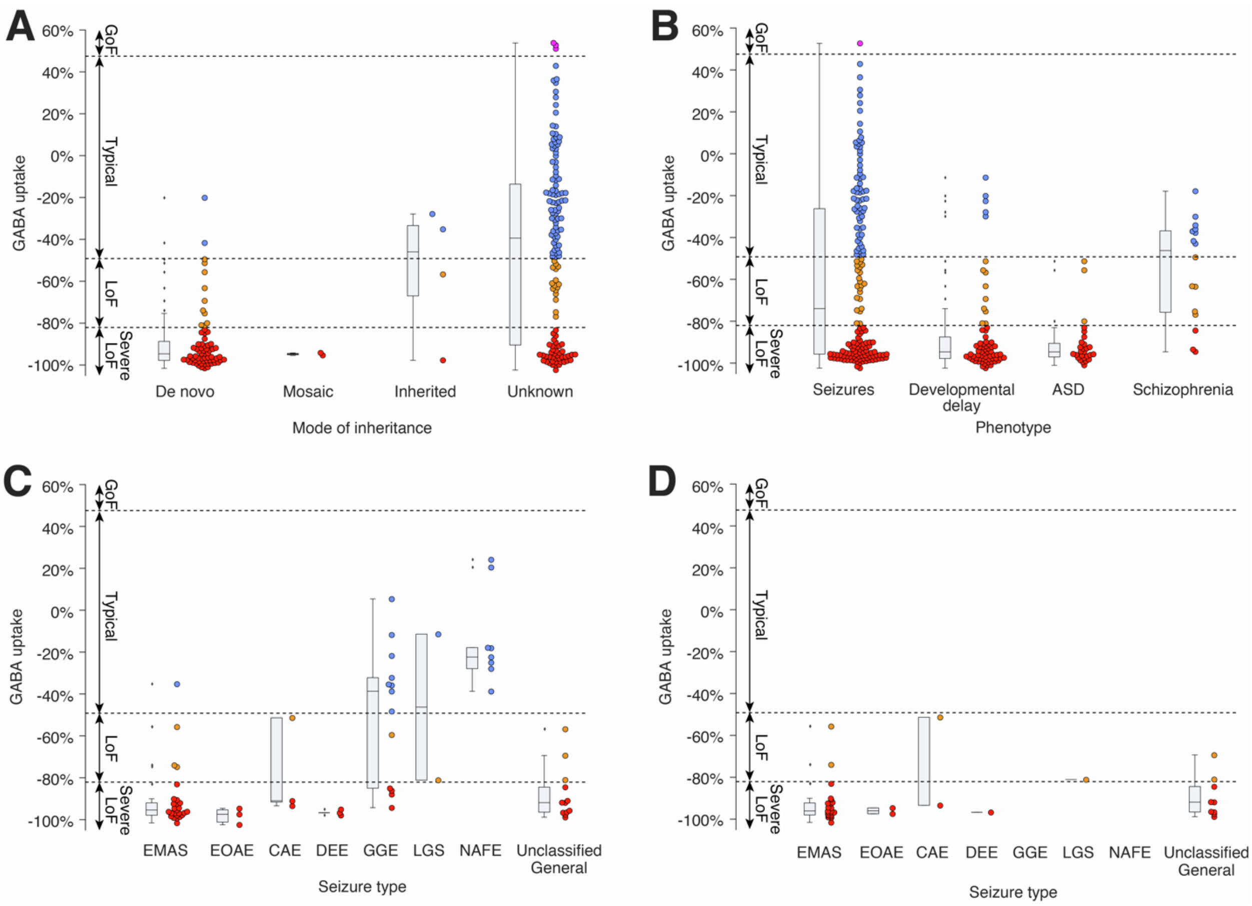

Figure S8. GABA uptake by inheritance, phenotype, and seizure type. A) GABA uptake data for 213 variants by reported mode of inheritance. Each variant is only represented once; recurrent variants with multiple modes of inheritance (e.g., *de novo* and inherited) are shown in the leftmost category of any individual (**Table S1**). **B)** GABA uptake data for 197 variants by diagnosis. If multiple diagnoses are reported for the variant it is reported for all diagnoses. Sixteen variants without a diagnosis are not shown (**Table S2**). **C)** GABA uptake data for 70 variants by seizure type; each variant is assigned to only one category (leftmost if multiple seizure types). A further 93 variants were associated with seizures without a specific seizure type listed. **D)** Panel 'C' is repeated for 36 variants reported to be germline *de novo*. Abbreviations: ASD: Autism spectrum disorder, NDD: neurodevelopmental delay, EMAS: Epilepsy with myoclonic atonic seizures, EOAE: Early-onset absence epilepsy, CAE: Childhood absence epilepsy, DEE: Developmental and epileptic encephalopathy, GGE: Genetic generalized epilepsy, LGS: Lennox-Gastaut Syndrome, NAFE: Non-familial non-acquired focal epilepsy.

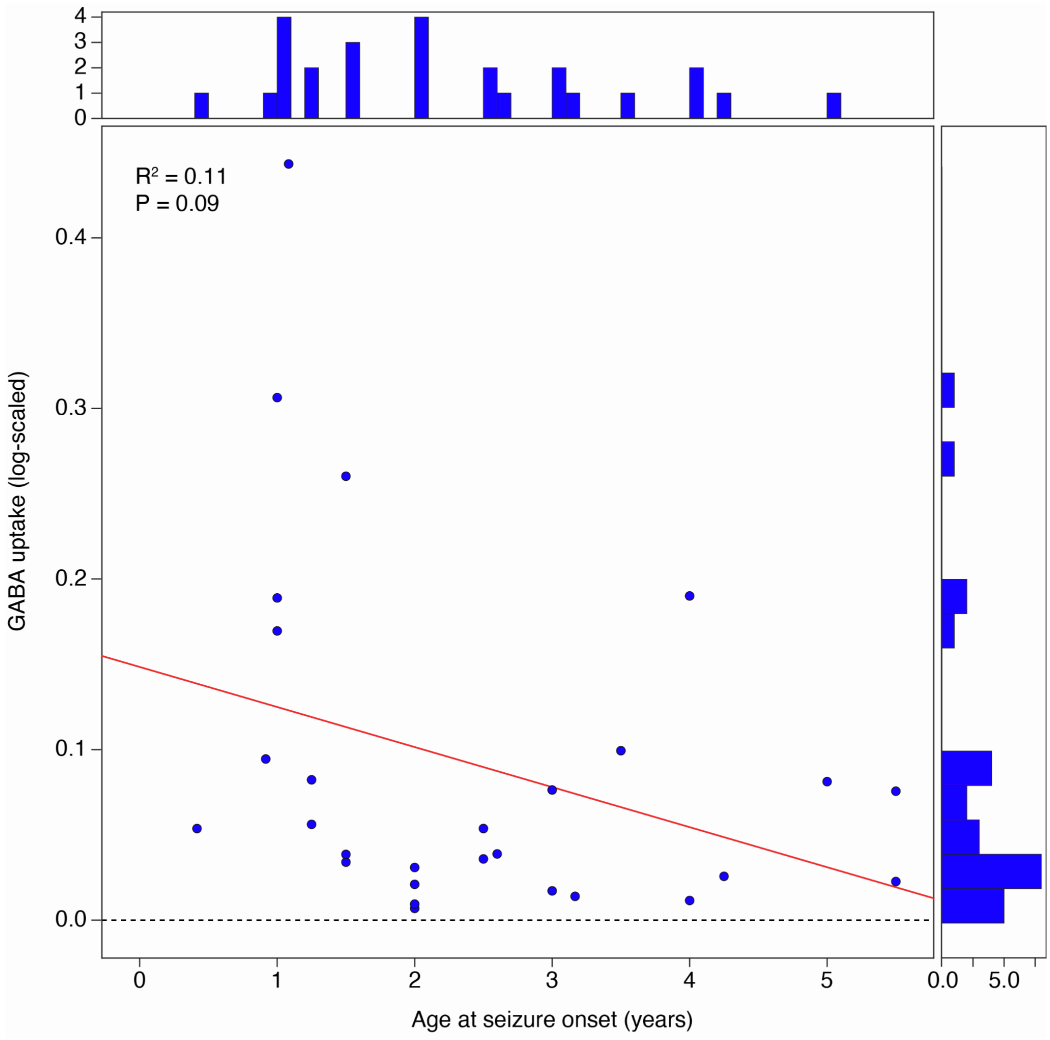

Figure S9. Relationship between GABA uptake and age of seizure onset. Relationship between GABA uptake functional data as a percentage of wildtype (y-axis) and age of seizure onset (x-axis) for all 34 *SLC6A1*variants where this information was reported.

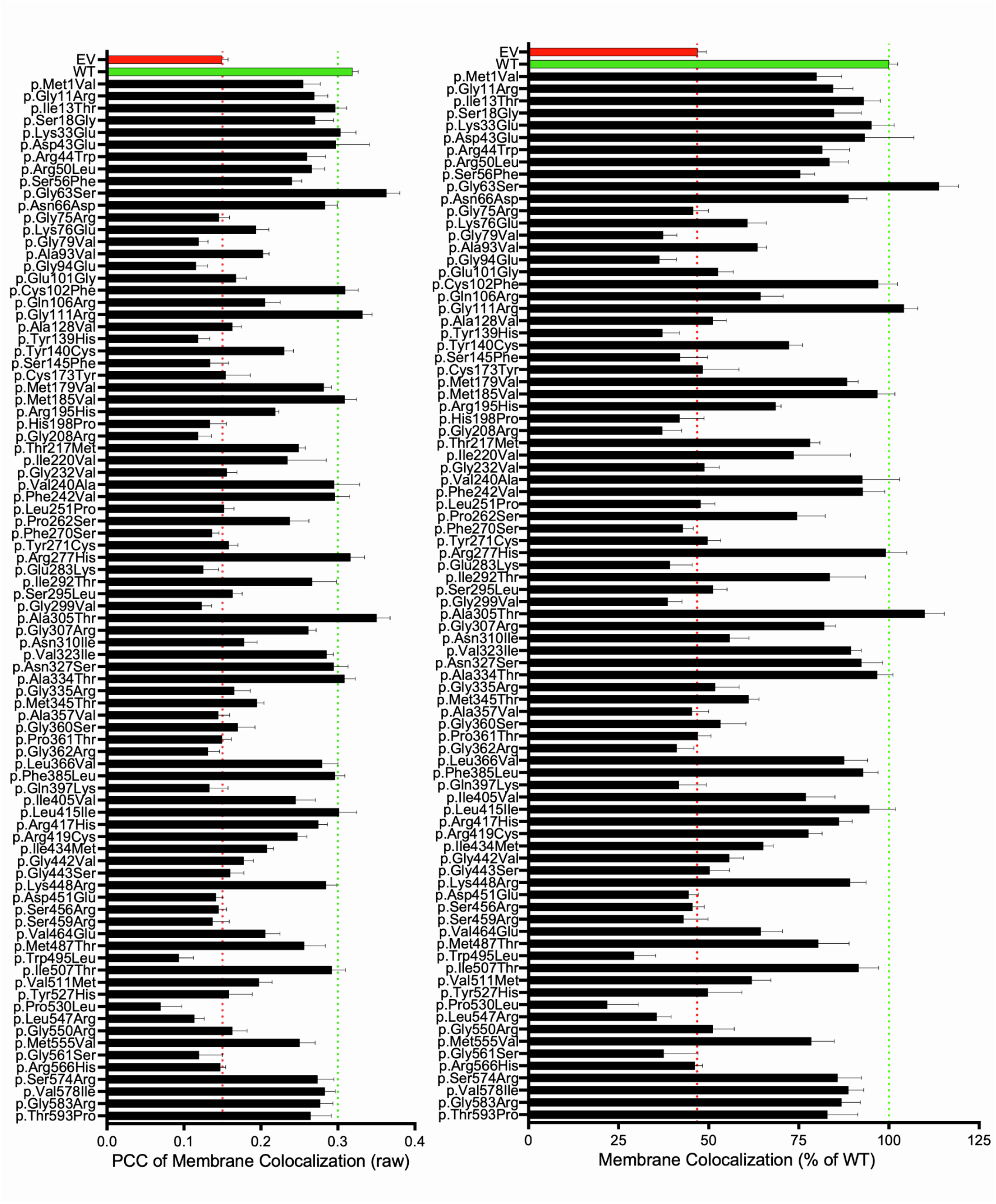

Figure S10: Quantitative results of high content imaging and colocalization of 86 GAT-1 variants. Represented as **A)** Pearson Correlation colocalization (PCC) values, and **B)** as a percent of wildtype. Error bars represent mean ±SEM of three biological replicates performed in triplicates.

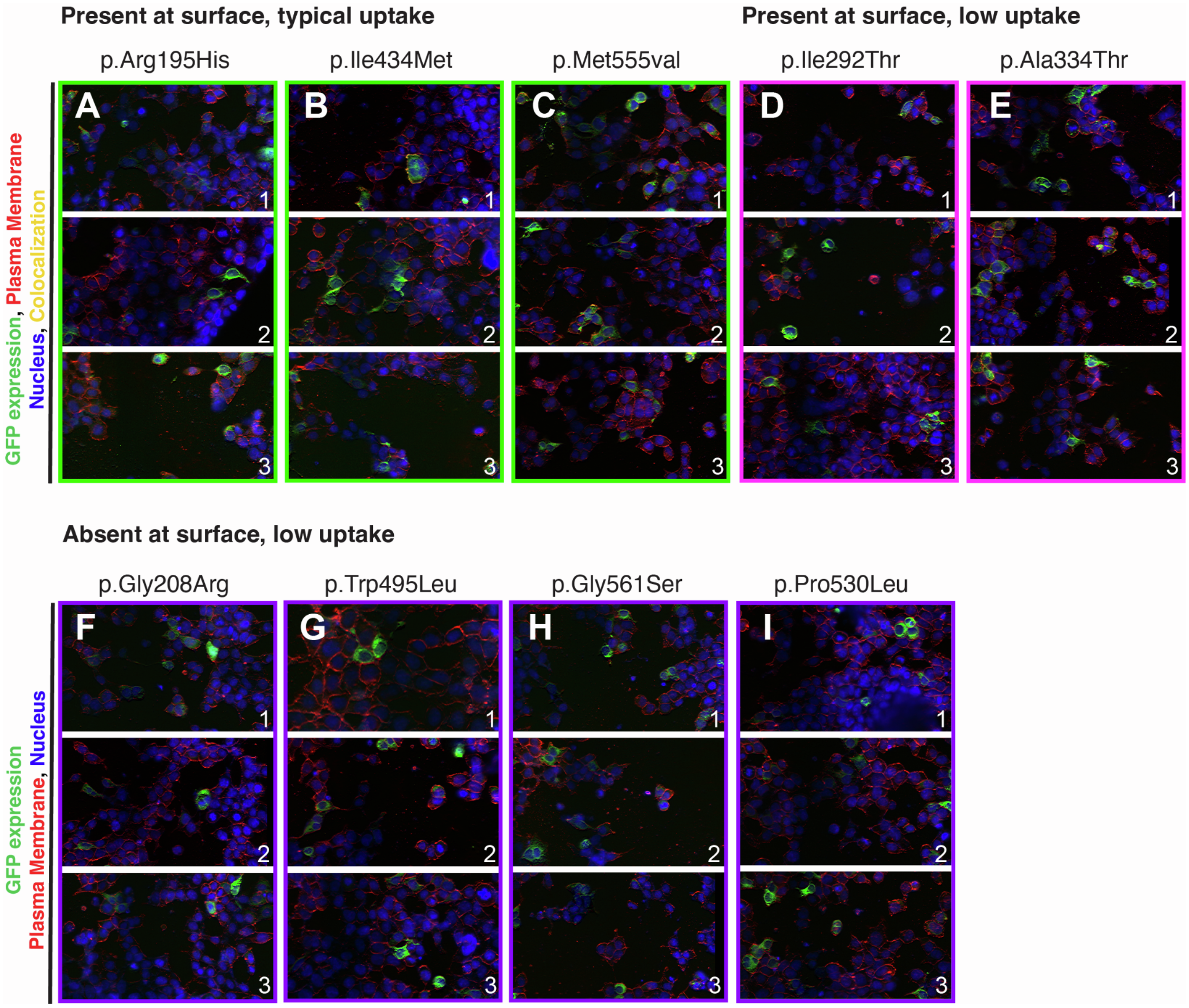

Figure S11: Raw images of high content imaging colocalization for a subset of missense variants. Images are divided by clusters of GAT-1 cell surface expression presence and uptake activity and each variant is shown with three different fields of view. **A, B, C)** Present at surface and typical GABA uptake. **D, E)** Present at surface, low GABA uptake. **F, G, H, I)** Absent at surface and low GABA uptake.

A Group 1 (green) - Typical uptake, present on the cell surface

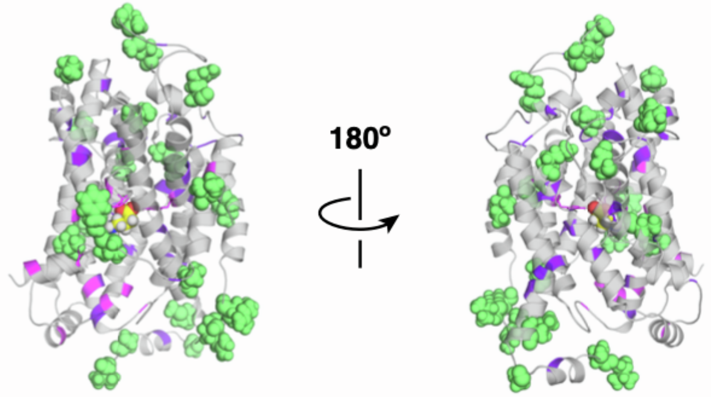

B Group 2 (purple) - Low uptake, absent on the cell surface

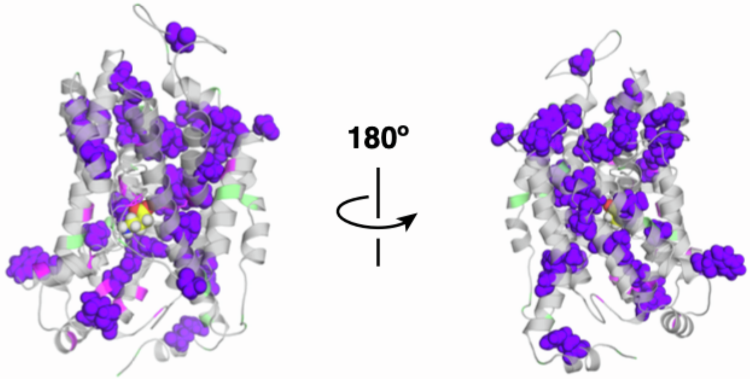

C Group 3 (pink) - Low uptake, present on the cell surface

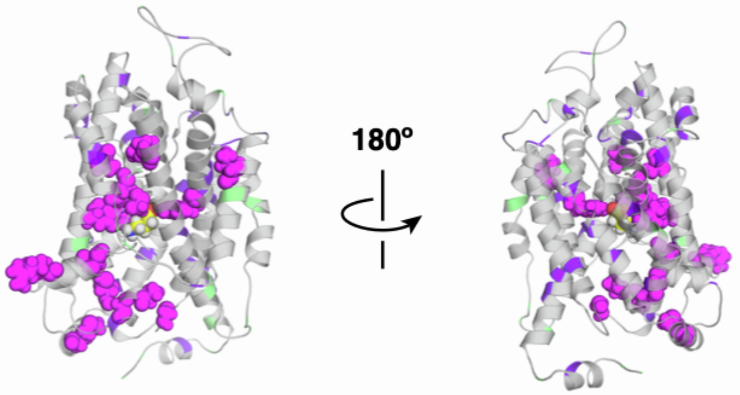

D

**Group**	outer-surface count	inner-surface count	**outer-surface (% of total)**	**inner-surface (% of total)**
1 - green	14	8	**63.6%**	**36.4%**
2 - purple	8	32	**20%**	**80%**
3 - pink	1	15	**6.3%**	**93.8%**

Figure S12. Mapping of GAT-1 variants onto 3D structure by group of surface expression and functional type. Variants are highlighted by group **A)** present at surface and typical GABA uptake, **B)** absent at surface, low GABA uptake, and **C)** present at surface and low GABA uptake. GABA is represented as a yellow molecule in all groups. **D)** Individual variants mapped in A, B, and C are counted whether they appear facing at the outer-surface of the protein or the inner-surface and represented as a percent of the total within that group.

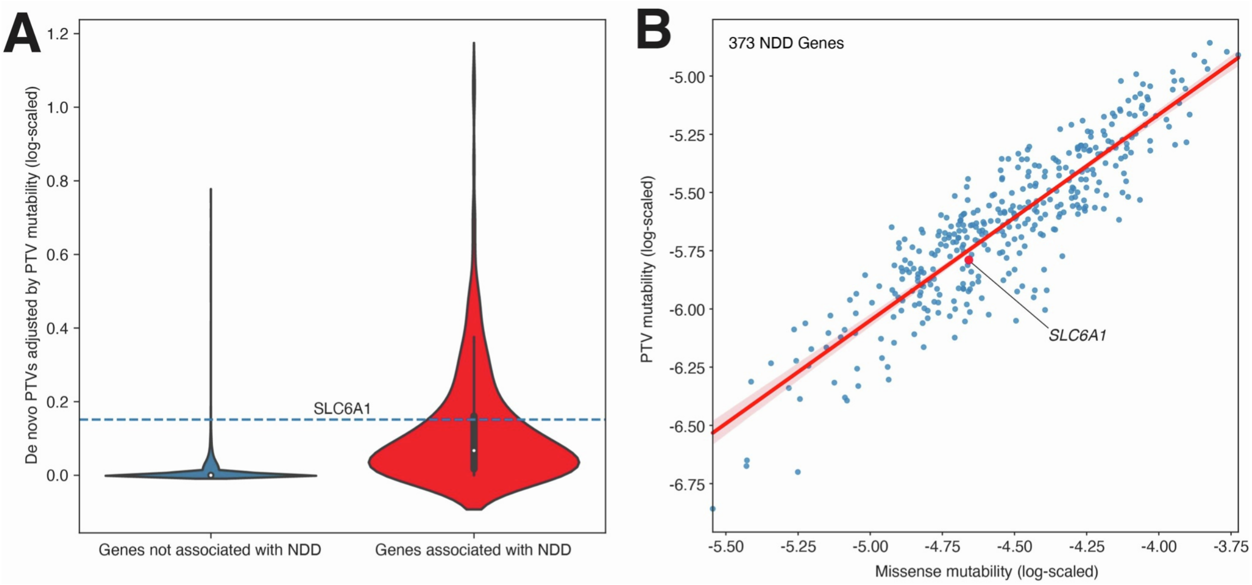

Figure S13: Frequency of *de novo* PTVs and gene-level mutability. A) The observed number of *de novo* PTVs in individuals with neurodevelopmental delay (NDD)[3] is divided by the estimated PTV mutability rate[4]. Metrics are shown for all autosomal protein-coding genes split into 373 genes associated with NDD (red, FDR ≤ 0.001) and 16,511 genes not associated with NDD (blue, FDR > 0.1). The equivalent value for SLC6A1 is shown as the blue dashed line. **B)** The mutability is shown for 373 NDD-associated genes for missense variants (x-axis) and PTVs (y-axis).

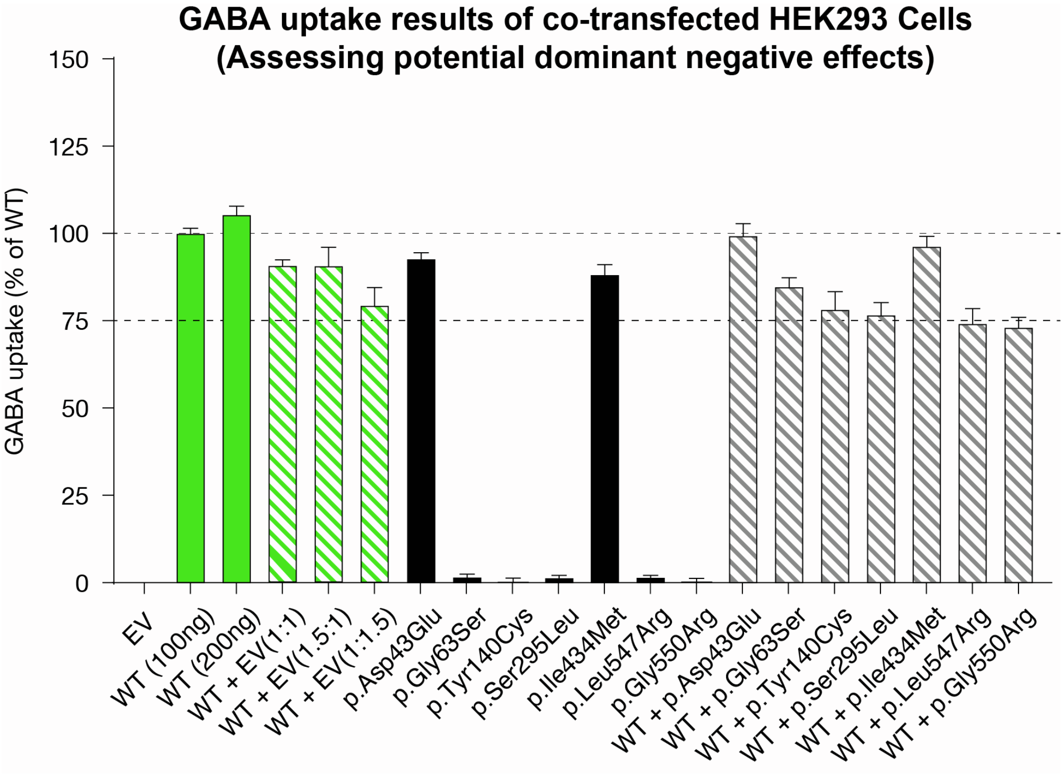

Figure S14: Assessing potential dominant negative effects in co-transfected HEK293 cells. Several distinct plasmid ratios of WT to EV are shown as positive controls of co-transfection (green stripes) and WT plus variant plasmids co-transfected are at a 1:1 plasmid ratio (gray stripes). Solid green and solid black bars represent single plasmids transfected into cells at a time. Results are shown as a percentage of wildtype (y-axis) and error bars represent mean ± SEM of 3 biological replicates. No significant difference is demonstrated between co-transfected experimentals and controls (stripes).

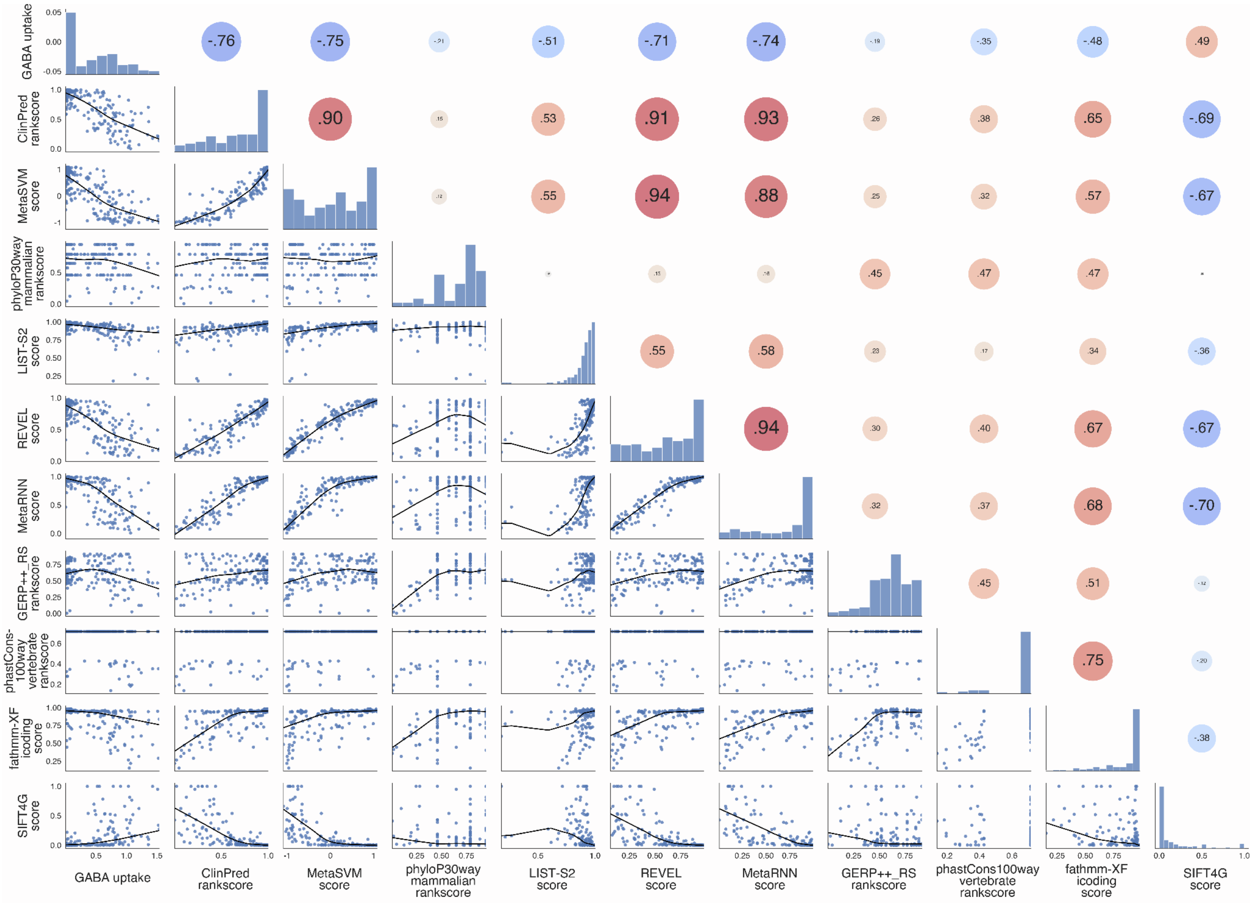

Figure S15: Correlation of the top ten prediction scores with GABA uptake. Relationship between observed GABA uptake values and top ten missense severity scores from stepwise linear regression model for 178 missense variants in *SLC6A1*. Each plot represents the comparison between two out of eleven metrics (GABA uptake and ten missense scores). Plots in the bottom left show a scatter plot with a Locally Weighted Scatterplot Smoothing (LOWESS) regression curve. Plots on the diagonal (i.e., each metric against itself) show a histogram of how the metric is distributed. Plots on the upper right show the Pearson correlation (text) with the size and shade of the dot increasing with the strength of the correlation and the color representing the direction of the correlation (blue for negative, red for positive).

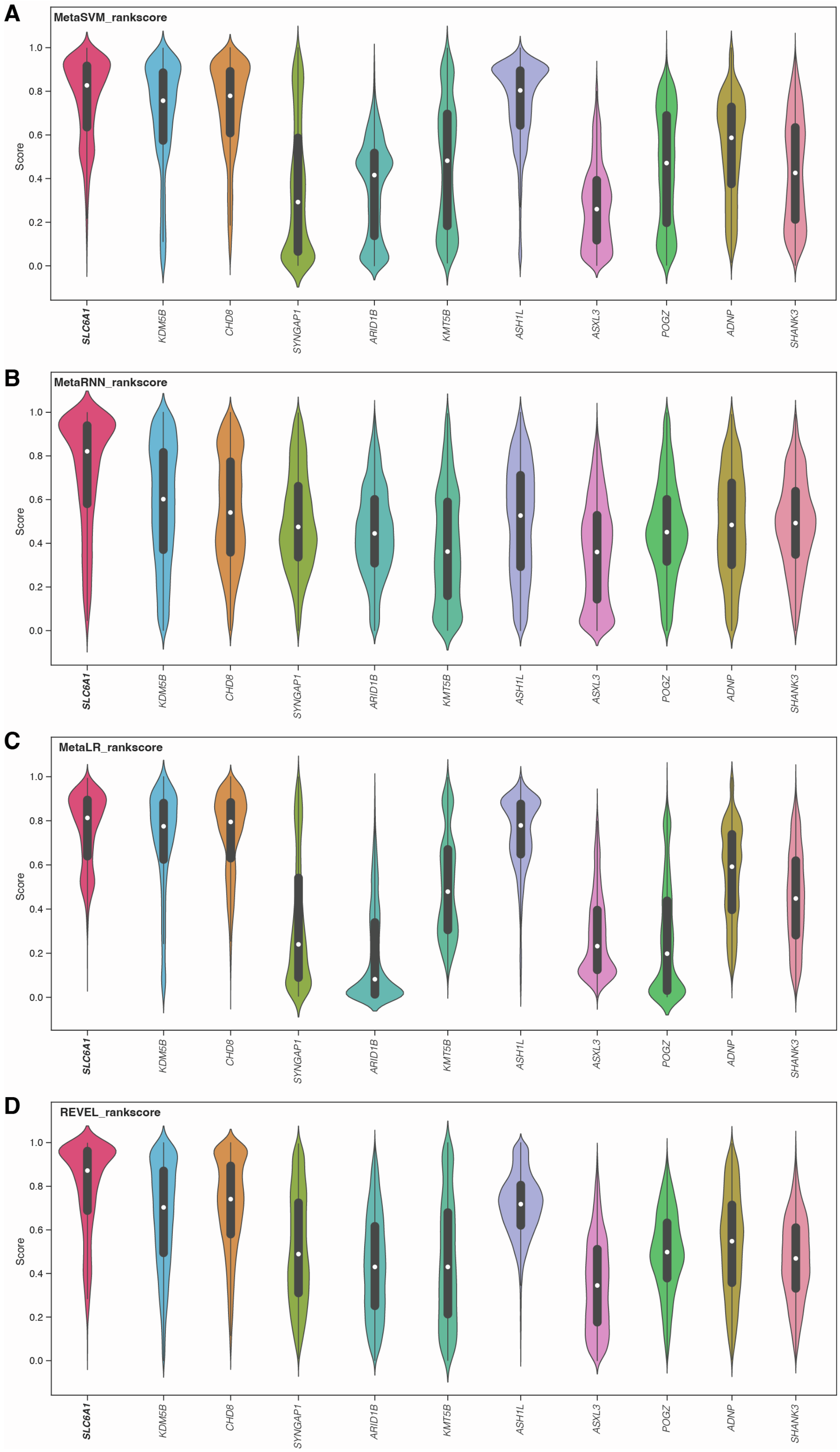
A
MetaSVM_rankscore
B
MetaRNN_rankscore
C
MetaLR_rankscore
D
REVEL_rankscore
Score
1.0
0.8
0.6
0.4
0.2
0.0
SLC6A1
KDM5B
CHD8
SYNGAP1
ARID1B
KMT5B
ASH1L
ASXL3
POGZ
ADNP
SHANK3

Figure S16: Distribution of missense severity scores in all possible missense variants in *SLC6A1* and ten PTV-enriched genes. Violin plots represent the distribution of **A)** MetaSVM, **B)** MetaRNN, **C)** MetaLR, and **D)** REVEL for all possible missense variants in *SLC6A1* genes and ten equivalent PTV-enriched ASD- and NDD-associated genes (See Fig. 4B).

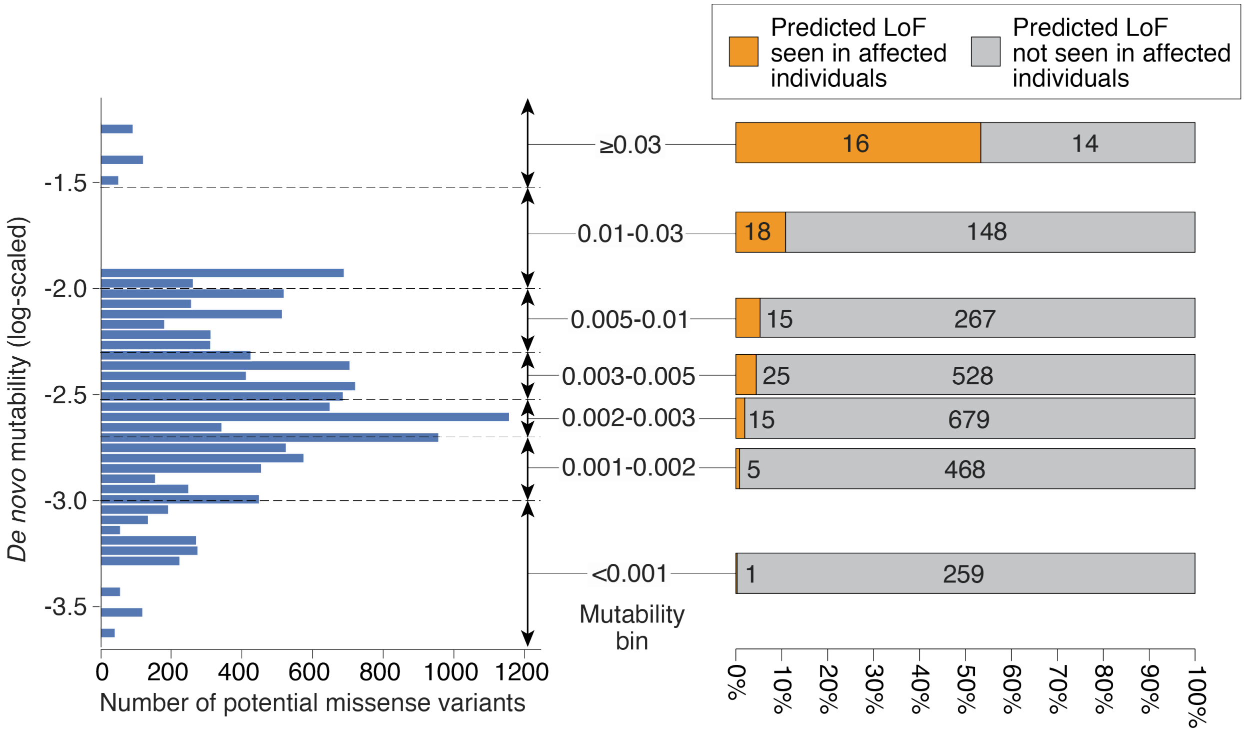

Figure S17. Mutability distribution of *SLC6A1* and observed frequencies for predicted loss-of-function variants. The distribution of *de novo* mutability, estimated from base-substitution mutation rate based on triplet DNA sequence from whole-genome sequencing of families is shown across all possible missense variants in *SLC6A1* (left graph). Mutability is used to define seven bins (middle). In each of these mutability bins, the total number of missense variants predicted to be loss-of-function and observed at least once in an affected individual is shown in orange, while the total number of missense variants predicted to be loss-of-function and not observed in affected individuals is shown in gray. Abbreviations: LoF: Loss-of-function.

Supplementary Tables

Table S1: Individual-level data. Excel spreadsheet in which each row represents an individual with data on the SLC6A1 variant and phenotype.

Table S2: Variant-level data. Excel spreadsheet in which each row represents a unique variant with data on the SLC6A1 variant and phenotype combined across individuals.

Table S3: Plasmid sequences. Excel spreadsheet with four sheets detailing the plasmids used for the GABA uptake and surface expression assays.

Table S4: GABA Uptake and Surface Expression data. Excel spreadsheet with four sheets detailing the results of the GABA uptake and surface expression assays.

Table S5: ClinVar Categories. Excel spreadsheet showing the relationship between ClinVar category and GABA uptake.

Table S6: Linear Regression. Excel spreadsheet with 10 sheets detailing the stepwise linear regression model building for missense variants.

Table S7: All SLC6A1 SNVs. Excel spreadsheet showing functional annotation, GAT-1 location, and missense severity scores (including predicted from linear regression model and random forest model) for all possible SNVs in SLC6A1 cDNA.

Supplemental Materials and Methods

The following data generation and data processing methods are separated by the two research groups: BioMarin and UCSF. The main materials and methods section of this manuscript has a summary of combined methods from both groups.

Initial variant selection (BioMarin)

We identified individuals with potentially pathogenic *SLC6A1* variants from multiple sources[2,4–15] (**Table S1**). The individuals and organizations who collected and generated this original data bear no responsibility for further analysis or interpretation in this publication. After taking into account individuals represented in more than one cohort, the total number of carriers was 213. After taking into account variants present in multiple individuals and after excluding large insertion or deletion variants (indels) and most noncoding variants, we arrived at 182 variants we successfully tested (**Table S2**). Of those, 90 were variants seen in individuals with epilepsy and developmental delay, 11 were seen in individuals with schizophrenia (3 of those with a high probability of being pathogenic), 73 were variants from various sources which are seen in individuals with epilepsy but for which pathogenicity has not been claimed and the remaining 5 were likely benign and included as negative controls (**Table S2**).

Initial variant selection (UCSF)

Individuals with variants in *SLC6A1* were identified from multiple sources, including: ClinVar[16], gnomAD[4], multiple cohort studies[3,5,7,17,18], case series and reports[7], and three previously undescribed individuals from the Moller group in Denmark (**Table S1**). The gnomAD aggregation database was used to select the 10 missense variants with the highest population frequencies as controls. Another 10 variants were selected as a representative group of protein truncating variants. The remaining 80 missense variants were selected based on: recurrence across multiple individuals, variants in individuals with detailed phenotyping data, and distribution across the GAT-1 protein. The full list of variants and extensive information on each can be found in **Table S2**.

Creation of *SLC6A1* Constructs (BioMarin)

The *SLC6A1* consensus coding-sequence (CCDS: CCDS2603.1) was synthesized by Genewiz (South Plainfield, NJ) and cloned into the pUC57- Kan plasmid with a CMV promoter driving the production of *SLC6A1*, opposite a reverse-oriented EF1-alpha promoter driving a Beta-lactamase reporter. Genscript (Piscataway, NJ) performed site-specific mutagenesis to generate 183 distinct variants. Mutagenesis was confirmed by Sanger sequencing.

Creation of *SLC6A1* Construct and GFP-tagged plasmids (UCSF)

The vector pCMV6-Entry containing the human cDNA of SLC6A1 (NM_003042) was purchased from OriGene (OriGene Technologies Inc., Rockville, MD, USA). The plasmid pCDNA5/FRT SLC6A1 (WT-SLC6A1) was created by digesting the pCMV-Entry SLC6A1 (OriGene) with KpnI and NotI (NEB) to excise the SLC6A1 transcript. The mammalian expression vector pcDNA5/FRT was also digested by KpnI and NotI to create compatible restriction sites. Digested DNA sequences and size was confirmed by MCLAB sequencing (South San Francisco, CA, USA). Ligation of the digested backbone and excised cDNA were performed using T4 Ligase and instructions were followed by manufacture protocol (New England BioLabs, Inc.). The resulting reaction was transformed and the plasmid was sequenced by MCLAB, confirming proper ligation of cDNA SLC6A1 into pcDNA5/FRT. The final WT-SLC6A1 plasmid was then tested by [3H]-GABA transport assay and the reuptake of GABA was compared to the original plasmid pCMV-

Entry SLC6A1. Compared to empty vector plasmid, both constructs demonstrated a 13-fold uptake of GABA, further confirming successful ligation.

The WT-SLC6A1 construct was sent to Genscript (Piscataway, NJ) and site-specific mutagenesis was performed to make 100 individual plasmids with its respective variant. Genscript internally confirmed the quality and sequence of all plasmids. For the GFP tagged plasmids, the 249 amino acid superfolder GFP (sfGFP, ASSN: ASL68970) was synthesized by Genscript and then cloned into the c-terminal end of SLC6A1 transcript in WT-SLC6A1 construct with no spacer or linker by the same company. The final plasmid, WT-SLC6A1-sfGFP, was verified by Genscript and tested in uptake assay to compare relative uptake to WT-SLC6A1. To create the 86 GFP tagged variant plasmids, Genscript performed site-directed mutagenesis on the WT-SLC6A1-sfGFP plasmid and verified every individual variant. All final sequences are reported in **Table S3**.

CRISPR/Cas9 knockout of *SLC6A1* in HEK293T cells (BioMarin)

We generated a *SLC6A1* deficient cell line, using HEK293T transfected with CRISPR/Cas9 RNP using the following crRNAs targeting exon 5 with PAM in bold:

- Hs.Cas9.SLC6A1.1.AD: 5'-[AGTGGCCAGCGGATCTGACC**TGG**]-3'
- Hs.Cas9.SLC6A1.1.AQ: 5'-[ATACACAAGGATCCAGGCGA**TGG**]-3'

Briefly, we annealed equimolar ratios of ATTO 550 labeled tracrRNA (IDT Cat. 1075928) and the above crRNA. Twelve picomoles of the resulting gRNA were combined with 104 pmol HiFi Cas9 (IDT 1081061) to form RNPs, according to the manufacturer's protocol. 2E5 HEK293T cells were transfected with RNP using a 96-well Shuttle nucleofector (Lonza) with Amaxa SF solution (Lonza) and program CM-138. Transfected cells were seeded into a 96-well plate and cultured for 3 days at 37°C, with 5% CO2, in 200 L of high-glucose DMEM (Invitrogen, Cat.11995-065) supplemented with 10% (v/v) Fetal Bovine Serum (VWR, Cat. 97068-085) and 1X GlutaMAX-ITM (GIBCO, Cat. 35050-061).

Next, single cells were sorted into 96-well plates containing 200 µL media using a FACSMelody cell sorter (BD Biosciences). Clones were monitored by imaging (Cell Metric, Solentim) until confluent, then expanded into six-well plates and genotyped. Genomic DNA was extracted using Quanta Extracta solution (Quanta Biosciences, Cat. 95091). DNA amplicons containing the gRNA target-region were produced using the following primers (*SLC6A1*-binding sequences in bold):

- FW: 5'-TCGTCGGCAGCGTCAGATGTGTATAAGAGACAG**GCTCCCACCAGCTCTGTGTA**-3'
- REV: 5'-GTCTCGTGGGCTCGGAGATGTGTATAAGAGACAG**CTATCCAGTGCCCTCCTGTCC**-3'

A second round of PCR incorporated NGS barcodes and Illumina sequencing-adapters via NexteraTM–Compatible Indexing Primers, which annealed to the adapters (underlined above) added during the first PCR. Amplicons were purified and pooled according to the manufacturer's instructions (Illumina Document # 15031942 v05), then paired-end sequenced (149x149) on a MiSeq 550 with a v2 reagent kit (Illumina, Cat. MS-102-2002). Indels were quantified using CRISPResso (version 2.0.27) to identify a clonal population with homozygous *SLC6A1* deficiency. The clonal cell line selected for experimentation was homozygous for a 37 bp deletion in exon 5:
5'-
[GCGCAACATGCATCAGATGACGGACGGGCTGGATAAGCCAGGTCCTGGATCCTTGTGTATTTCTGT
ATCTGGAAGGGTGTTGGCTGGACTGGAAAG]-3'

Resulting in the following truncated *SLC6A1* protein coding sequence:
MATNGSKVADGQISTEVSTEVSEAPVANDKPKTLVVKVQKKAADLPDRDTWKGRFDFLMSCVGYAIGLG
NVWRFPYLCGKNGGGAFLIPYFLTLIFAGVPLFLLECSLGQYTSIGGLGVWKLAPMFKGVGLAAAVLSFW

LNIYYIVIISWAIYYLYNSFTTTLPWKQCDNPWNTDRCFSNYSMVNTTNMTSAVVEFWERNMHQMTDGLD KPGPGSLCISVSGRVLAGLERWSSTFQPHTPTSC*

The GABA uptake assay was used to confirm loss of SLC6A1 activity in the knockout line.

Cell culture preparation of HEK293-FlpIn cells and creation of transient and stable cell lines (UCSF)

Human embryonic kidney cells (HEK293) with the Flp-In integration system (Life Technologies Corporation, Carlsbad, CA, USA) were used for this study. Cell culture preparation of HEK293 cells requires growth and maintenance in Dulbecco’s Modified Eagle Medium (DMEM) media with 100 units/mL penicillin, 100 units/mL streptomycin, and 10% fetal bovine serum (FBS). Stable or transient cell lines were transfected with the WT-SLC6A1 plasmid or the empty vector pcDNA5/FRT (EV) cDNA in HEK293 cells. The reagent Lipofectamine LTX (Life Technologies Corporation, Carlsbad, CA, USA) was used to transfect cells transiently in triplicates on 3 separate days or stably. Appropriate DNA to LTX ratios were followed according to the manufacturer's protocol (final 2:1). Using poly-D-lysine treated 96-well plates and an optimized seeding density of $3.3x10^5$ cells/well, cells were reverse transfected with 200 ng of DNA and 0.4 µL of Lipofectamine LTX in Opti-MEM medium (Life Technologies Corporation) to transfect each well. FBS only DMEM media is used on seeding and transfecting day. Plates were left for 45 minutes in the hood at room temperature before placing in the incubator with 5% CO_2 at a stable 37 ℃ to allow cells to settle and spread out evenly on the well surface. Cells were grown for 48 hours post-reverse transfection then assayed for GABA transport. To create stable cell lines, HEK293 Flp-In cells were transfected using Lipofectamine LTX with a 1:2 DNA (in pcDNA5/FRT mammalian expression vector) to LTX ratio and a 1:9 ratio of DNA to pOG44 (Flp-Recombinase expression vector) in a 6-well plate treated with poly-D-lysine. After 48 hours, cells were split into a 10 mm dish and allowed to sit overnight. Positive clone selection began the next day by using fresh DMEM media with 100 µg/mL of Hygromycin B (Life technologies Corporation) and changed every 2-3 days for about two weeks.

Transfection of Variants and GABA uptake assay (BioMarin)

HEK293T *SLC6A1*-deficient cells were transfected in sextuplicate, using a 96-well Shuttle nucleofector (Lonza) integrated onto a MICROLAB STAR liquid-handling robot (Hamilton Robotics, Reno, NV). Briefly, 250 ng of plasmid per 300,000 cells was transfected using Amaxa solution SF (Lonza) and program CM-138. Cells were seeded onto 96-well plates, then cultured for 3 days in 200 µL media, as described above.

Prior to the reuptake assay, cells were centrifuged at 300 xg for 5 min and all media was aspirated. Cells were then incubated at 37°C for 15 min with 80 µL of Pre-Incubation solution containing 140 mM NaCl, 5 mM KCl, 2 mM CaCl2, 1 mM MgSO4, 2 nM Glucose, 2.5 mM HEPES at pH 7.4 and osmolarity 310. Pre-incubation solution was aspirated, following centrifugation at 300 xg for 5 min. Cells were incubated for 30 min at room temperature with 100 µL of 4800 nM d6-GABA (SIGMA, Cat. 615587) in a pre-incubation solution. After centrifuging for 5 min at 300 xg, the uptake solution was aspirated, and cells were washed twice with 80 µL preincubation solution. Supernatant was aspirated and plates were flash frozen at -80°C. Cells were harvested by adding 60 µL MPER (Thermo Fisher Scientific, Cat. 78501) and lysed with rigorous pipetting. 10 µL of lysate was used to determine protein concentrations with a Pierce BCA Protein Assay (Thermo Fisher Scientific, Cat. 23227), according to the manufacturer’s instructions.

Beta-lactamase (BLA) activity was determined by measuring hydrolysis of d7-penicillin G (Toronto Research Chemicals, Cat. B288600) into d7-5R,6R-benzylpenicilloic acid (d7-BPA). The BLA Assay Cocktail consisted of 60 µM d7-penicillin G substrate and 1.5 µM 5R,6R- benzylpenicilloic

acid (Toronto Research Chemicals, Cat. B288593), in 50 mM Tris-HCl (pH 7.5±0.02). For absolute quantification, separate BPA and d6-GABA standard curves were prepared with the following ranges: 120 µM, 60 µM, 20 µM, 10 µM, 2 µM, 1 µM and 200 nM for BPA; and 6 µM, 3 µM, 1 µM, 500 nM, 100 nM, 50 nM, 10 nM, 1 nM and 500 pM for GABA. To avoid hydrolysis, d7-penicillin G and BPA were stored dry, under inert gas at -20°C, and the BLA Assay Cocktail and BPA standards were prepared fresh. BPA standards and lysates were processed in parallel with sample lysates. Briefly, 20 µL of BLA Assay Cocktail was combined with 20 µL lysate or BP A standard, then incubated for 1 hour at 37°C, on an orbital shaker set to 500 rpm. The reaction was quenched with 160 µL of acetonitrile containing 25nM d2-GABA (Sigma, Cat. 617458), then vortexed at 1500 rpm for 5 min and centrifuged at 3000×G for 10 min. 100 µL of supernatant was combined with an equal volume of HPLC-grade water and reserved for the BLA-assay injection. An additional 20 µL of supernatant was combined with 10 µL 100 mM Sodium Carbonate in a 96-well plate, then vortexed for 5 min at 1500 rpm. GABA was derivatized with 10 µL 6% (v/v) Benzoyl-Chloride in acetonitrile, vortexing at 1500 rpm for 5 min. The reaction was quenched with 0.2% Formic Acid (v/v) in water, then centrifuged at 4000 rpm for 4 min, to produce the final GABA analyte.

UPLC-MS/MS analysis was performed on an Agilent 6495 Triple-Quadrupole LC/MS, equipped with an Agilent 1290 Infinity II series HPLC. Separation of analytes was achieved using an Acquity UPLC BEH C18 1.7 µm, 2. x 50 mm column (Waters Crop., Cat. 176001692) at 40°C. Mobile phase A (MPA) contained water with 0.1% formic acid, and mobile phase B (MPB) was acetonitrile with 0.1% formic acid. The BLA and GABA analytes were analyzed in separate, 10 µL injections, utilizing the same LC-MSMS instruments and mobile phases, but different gradients. For the BLA assay, a flowrate of 0.4 mL/min was applied with the following gradient: 0-1 min, 5% mobile phase B; 1-2 min, 5%-95% MPB; 2-2.9 min, 95% MPB; 2.9-3 min 5% MPB. The enzymatic product (d7-5R,6R-benzylpenicilloic acid) and internal standard (5R,6R- benzylpenicilloic acid) were detected through multiple reaction monitoring (MRM) using the following transitions: 360.3 > 160 and 353.3 > 160, respectively. For the GABA assay, the flowrate was set to 0.4 mL/min with the following gradient: 0-4 min, 5-42% mobile phase B; 4- 4.4 min, 42%-95% MPB; 4.4-4.9 min 95% MPB; 4.9-5% MPB. We monitored the GABA transported into cells by SLC6A1 (d6-GABA-BZ, following derivatization) and the internal standard (benzoylated d2-GABA) by MRM, using distinct transitions: d6-GABA-BZ, 214.1 > 105; d2-GABA-BZ, 210.1 > 105. In both assays, the dwell time was set to 50 msec. The cone voltage in both assays was 4500 V and the collision energy varied, using 17 V in the GABA assay and 11 V for the BLA method. Method scheduling was performed in Mass Hunter (Agilent, version 10.0). Peak calling and standard-curve calculations were executed in QQQ Quantitative Analysis (Agilent, version 10.1).

[^{3}H]-GABA Transport Assay (UCSF)

HEK293 cells were transiently transfected as described above in 96-well plates. Uptake of [^{3}H]-GABA was assayed 48 hours post-reverse transient transfection or 24 hours after plating stable cells. Warm choline buffer (150 mM choline chloride, 5 mM KP_i, 0.5 mM $MgSO_4$, and 0.3 mM $CaCl_2$, pH 7.4) was used to wash cells twice on the day of assaying. Cells were then incubated with the same choline solution for 30 minutes in the incubator. Transport solution containing 150 mM NaCl, 5 mM KP_i, 0.5 mM $MgSO_4$, and 0.3 mM $CaCl_2$ at pH 7.4 was used to dilute 4 µCi of [^{3}H]-GABA (35 Ci/mmol). The choline solution from each well was then replaced with prepared [^{3}H]-GABA transport solution for an incubation of 10 minutes. Prior to beginning this study, the transport assay was studied at an incubation of 10 minutes, 15 minutes and 20 minutes. No difference in uptake was apparent. After 10 min, the reaction was then slowed down and terminated by washing the cells twice with ice-cold NaCl transport buffer only. Cells were completely aspirated of solution and lysed with 0.28 mL of lysis buffer (0.1N sodium hydroxide (NaOH) and 1% v/v SDS solution). After shaking the cells with lysis buffer for 60-90 min, 0.25 mL

of cell lysate was transferred to scintillation fluid (EcoLite(+)™, MP Biomedicals, Santa Ana, CA, USA) for scintillation counting on the Beckman Coulter LS6500. The remaining lysate (20 μL) was used to measure total protein using the BCA assay kit. Uptake values were corrected for protein concentration using BCA values for each well.

Transfections for dominant negative effect (UCSF)

Dominant negative study design was optimized from previous groups[19–21]. HEK293 cells were seeded at an optimized cell density of $3.3x10^5$ cells/well in a poly-D-lysine treated 96-well plate and reverse transfected with respective plasmid(s) on the same day. The reagent Lipofectamine LTX (Life Technologies Corporation, Carlsbad, CA, USA) was used to transfect cells transiently in triplicates. Using the manufacturing protocol, we optimized our DNA to LTX ratio to 2:1, or 2 μL of Lipofectamine LTX for every 1 μg of DNA. A master mix with DNA, LTX and Opti-MEM medium (Life Technologies Corporation) is prepared according to the manufacturer's protocol of the LTX reagent. For dominant negative studies, we tested a 1:1 ratio of two distinct plasmids co-transfected in the same well, with a total of 100 ng per well. For example, the WT-SLC6A1 plasmid was mixed with a mutated SLC6A1 plasmid (50 ng WT:50 ng Mut) during master mix preparation. For those wells transfected with control or mutant only, we tested 200 ng, 100 ng, and 50 ng of the plasmid alone to compare activity to co-transfections and evaluate transfection efficiency. FBS only DMEM media is used on seeding/transfecting day. Plates were left for 45 minutes in the hood at room temperature before placing in the incubator with 5% CO_2 at a stable 37 ℃ to allow cells to settle and spread out evenly on the well surface. Cells were grown for 48 hours post-reverse transfection then assayed for GABA transport as described above (UCSF).

Data analysis (BioMarin)

BLA activity (nmol/hr/mg_protein) was determined using nanomoles d7-BPA, as calculated from the BPA standard curve, then divided by the incubation time (1 hour). This rate was further divided by the milligrams of lysate protein, as a proxy for variable cell counts between wells. SLC6A1 re-uptake activity (nmol/hr/mg_protein) was calculated similarly, by determining the nanomoles of d6-GABA from the d2-GABA standard curve, then dividing by both the incubation time (0.5 hours) and milligrams of protein in the lysate. To account for variable transfection and expression efficiencies, SLC6A1 re-uptake activity (nmol/hr/mg_protein) was then divided the BLA activity, to derive the GABA uptake normalized to BLA activity. While the BLA-normalized GABA activity is an accurate measure of a variant's transport activity, the functional consequence of a variant is best described in relation to wildtype-GAT-1 transport—to convey this, we calculated "Percent Wildtype" activities, where each variant's BLA-normalized SLC6A1-activity was divided by the average BLA-normalized SLC6A1 activity of all wildtype replicates present within an assay plate reported in **Table S4**. Figures were generated in Python matplotlib, seaborn, or SankeyMATIC (https://sankeymatic.com/). Figure 6 was generated using Protter (version 1.0)[22].

Distribution of measurement variation (BioMarin)

To account for variability in transfection efficiencies, GABA reuptake activity was normalized to activity of a Beta-lactamase (BLA) reporter present on the *SLC6A1* expression construct. BLA activity was determined by measuring hydrolysis of d7-penicillin G. While the BLA-normalized GABA reuptake activity is an accurate measure of a variant's transport activity, there exists experimental variability that is biased towards *SLC6A1* variants with high activity. The experimental variation is heightened due to the high sensitivity of the mass spectrometry assays which can detect even slight variations that could be caused by miniscule pipetting errors. Ultimately biological replicates were included to assess the variability in transporter activity of SLC6A1 variants which was normalized to activity in wildtype cells.

Stepwise linear regression

To build a predictor of missense severity in *SLC6A1*, we annotated all 179 missense variants with functional data from BioMarin and/or UCSF against estimates of mutability from whole-genome sequence of families[23], numerous predictors of missense severity using annoVar protocol 'dbnsfp42a' and build 'hg38'[[24]], and estimates of protein stability: DynaMut[25] and DeepDDG[26]. Linear regression was performed for all 81 quantitative variables against 147 missense variants with values for all variables (**Table S2**). The lowest p-value was achieved by ClinPred rankscore ($P=2.6x10^{-23}$, $R^2=0.49$). Linear regression was repeated for the other 80 variables, but ClinPred rankscore was included in the model too. This process of picking the top ranked variable by P-value was repeated for 10 rounds, achieving a model with an R^2 of 0.63. Three variables were only available for a subset of missense variants: MutPred (166 variants), DynaMut (160 variants), DeepDDG (160 variants). Of these only MutPred score was included in the model (step 4, P=0.02). To assess if increasing the number of variants improved the model, we repeated the stepwise linear regression excluding MutPred, DynaMut, and DeepDDG. This yielded 177 missense variants with functional data and annotations for all 78 quantitative metrics. Repeating the linear regression (**Table S6**), the lowest p-value was again achieved by ClinPred rankscore ($P=7.1x10^{-35}$, $R^2=0.58$, beta=-1.085, intercept=0.204) and ten rounds of stepwise linear regression led to a model with an R^2 of 0.68. Given the risk of overfitting to a relatively small dataset, we elected to use the ClinPred rankscore metric alone (scaled from no predicted impact at 0 to severe predicted impact at 1).

To extrapolate the GABA uptake functional data to all possible missense variants in *SLC6A1* we used the ENST00000287766.10 transcript for the ENSG00000157103.12 *SLC6A1* gene as defined by GENCODE v39 and the GRCh38/hg38 genome build to define every possible DNA variant in *SLC6A1* (**Table S7**). Variants were annotated as for the variants with GABA uptake data and the ClinPred rankscore was used to predict GABA uptake (**Table S7**).

Random forest analysis

We performed a random forest model to provide a second regression method predicting functional uptake. We used the R packages 'randomForest' and 'caret', and R defaults for number of trees sampled, 500, and number of independent variables sampled at each split, 2. Built in out-of-bag error estimates were used to evaluate performance.

Initial independent variables included location of variant (represented by exon number), initial amino acid, altered amino acid, and mutability score. Using the training set consisting of 179 missense variants with protein uptake set as the outcome variable, we analyzed 75 pre-existing prediction tools to optimize variance explained by the model. We iteratively added the best performing tool until addition of another tool led to <1% improvement in variance explained. MetaRNN raw score, ClinPred rankscore, and Provean converted rankscores were added to the model in that order via this method. This model resulted in between 54.13-55.18% of the variance explained over the course of ten runs.

This model was then applied to known possible missense variants of the GAT-1 protein. Predicted uptake for all possible missense variants are included in **Table S7**. When converting continuous uptake outcomes to equivalent categorical outcomes prespecified in main body of the paper, 414 fell into the Severe LoF range, 1,735 were predicted to fall into the LoF range, 1,803 were predicted to be wild type, and 0 were predicted in the gain of function range.

Supplemental References

1. Mermer, F., Poliquin, S., Rigsby, K., Rastogi, A., Shen, W., Romero-Morales, A., Nwosu, G., McGrath, P., Demerast, S., Aoto, J., et al. (2021). Common molecular mechanisms of SLC6A1 variant-mediated neurodevelopmental disorders in astrocytes and neurons. Brain *144*, 2499–2512. 10.1093/brain/awab207.

2. Mattison, K.A., Butler, K.M., Inglis, G.A.S., Dayan, O., Boussidan, H., Bhambhani, V., Philbrook, B., da Silva, C., Alexander, J.J., Kanner, B.I., et al. (2018). SLC6A1 variants identified in epilepsy patients reduce γ-aminobutyric acid transport. Epilepsia *59*, e135–e141. 10.1111/epi.14531.

3. Fu, J.M., Satterstrom, F.K., Peng, M., Brand, H., Collins, R.L., Dong, S., Wamsley, B., Klei, L., Wang, L., Hao, S.P., et al. (2022). Rare coding variation provides insight into the genetic architecture and phenotypic context of autism. Nat Genet *54*, 1320–1331. 10.1038/s41588-022-01104-0.

4. Karczewski, K.J., Francioli, L.C., Tiao, G., Cummings, B.B., Alföldi, J., Wang, Q., Collins, R.L., Laricchia, K.M., Ganna, A., Birnbaum, D.P., et al. (2020). The mutational constraint spectrum quantified from variation in 141,456 humans. Nature *581*, 434–443. 10.1038/s41586-020-2308-7.

5. Rees, E., Han, J., Morgan, J., Carrera, N., Escott-Price, V., Pocklington, A.J., Duffield, M., Hall, L.S., Legge, S.E., Pardiñas, A.F., et al. (2020). De novo mutations identified by exome sequencing implicate rare missense variants in SLC6A1 in schizophrenia. Nat Neurosci *23*, 179–184. 10.1038/s41593-019-0565-2.

6. Cai, K., Wang, J., Eissman, J., Wang, J., Nwosu, G., Shen, W., Liang, H.-C., Li, X.-J., Zhu, H.-X., Yi, Y.-H., et al. (2019). A missense mutation in SLC6A1 associated with Lennox-Gastaut syndrome impairs GABA transporter 1 protein trafficking and function. Exp Neurol *320*, 112973. 10.1016/j.expneurol.2019.112973.

7. Goodspeed, K., Pérez-Palma, E., Iqbal, S., Cooper, D., Scimemi, A., Johannesen, K.M., Stefanski, A., Demarest, S., Helbig, K.L., Kang, J., et al. (2020). Current knowledge of SLC6A1-related neurodevelopmental disorders. Brain Commun *2*, fcaa170. 10.1093/braincomms/fcaa170.

8. Friesner, R.A., Murphy, R.B., Repasky, M.P., Frye, L.L., Greenwood, J.R., Halgren, T.A., Sanschagrin, P.C., and Mainz, D.T. (2006). Extra precision glide: docking and scoring incorporating a model of hydrophobic enclosure for protein-ligand complexes. J Med Chem *49*, 6177–6196. 10.1021/jm051256o.

9. Carvill, G.L., McMahon, J.M., Schneider, A., Zemel, M., Myers, C.T., Saykally, J., Nguyen, J., Robbiano, A., Zara, F., Specchio, N., et al. (2015). Mutations in the GABA Transporter SLC6A1 Cause Epilepsy with Myoclonic-Atonic Seizures. Am J Hum Genet *96*, 808–815. 10.1016/j.ajhg.2015.02.016.

10. Epi25 Collaborative (2019). Ultra-Rare Genetic Variation in the Epilepsies: A Whole-Exome Sequencing Study of 17,606 Individuals. Am J Hum Genet *105*, 267–282. 10.1016/j.ajhg.2019.05.020.

11. Firth, H.V., Richards, S.M., Bevan, A.P., Clayton, S., Corpas, M., Rajan, D., Van Vooren, S., Moreau, Y., Pettett, R.M., and Carter, N.P. (2009). DECIPHER: Database of Chromosomal Imbalance and Phenotype in Humans Using Ensembl Resources. Am J Hum Genet *84*, 524–533. 10.1016/j.ajhg.2009.03.010.

12. Heyne, H.O., Singh, T., Stamberger, H., Abou Jamra, R., Caglayan, H., Craiu, D., De Jonghe, P., Guerrini, R., Helbig, K.L., Koeleman, B.P.C., et al. (2018). De novo variants in neurodevelopmental disorders with epilepsy. Nat Genet *50*, 1048–1053. 10.1038/s41588-018-0143-7.

13. Johannesen, K.M., Gardella, E., Linnankivi, T., Courage, C., de Saint Martin, A., Lehesjoki, A.-E., Mignot, C., Afenjar, A., Lesca, G., Abi-Warde, M.-T., et al. (2018). Defining the phenotypic spectrum of SLC6A1 mutations. Epilepsia *59*, 389–402. 10.1111/epi.13986.

14. Zafar, S., and Jabeen, I. (2018). Structure, Function, and Modulation of γ-Aminobutyric Acid Transporter 1 (GAT1) in Neurological Disorders: A Pharmacoinformatic Prospective. Front. Chem. *6*, 397. 10.3389/fchem.2018.00397.

15. Skovstrup, S., Taboureau, O., Bräuner-Osborne, H., and Jørgensen, F.S. (2010). Homology Modelling of the GABA Transporter and Analysis of Tiagabine Binding. ChemMedChem *5*, 986–1000. 10.1002/cmdc.201000100.

16. Landrum, M.J., Lee, J.M., Benson, M., Brown, G.R., Chao, C., Chitipiralla, S., Gu, B., Hart, J., Hoffman, D., Jang, W., et al. (2018). ClinVar: improving access to variant interpretations and supporting evidence. Nucleic Acids Res *46*, D1062–D1067. 10.1093/nar/gkx1153.

17. Kaplanis, J., Samocha, K.E., Wiel, L., Zhang, Z., Arvai, K.J., Eberhardt, R.Y., Gallone, G., Lelieveld, S.H., Martin, H.C., McRae, J.F., et al. (2020). Evidence for 28 genetic disorders discovered by combining healthcare and research data. Nature *586*, 757–762. 10.1038/s41586-020-2832-5.

18. Epi25 Collaborative (2021). Sub-genic intolerance, ClinVar, and the epilepsies: A whole-exome sequencing study of 29,165 individuals. Am J Hum Genet *108*, 965–982. 10.1016/j.ajhg.2021.04.009.

19. Stergachis, A.B., Pujol-Giménez, J., Gyimesi, G., Fuster, D., Albano, G., Troxler, M., Picker, J., Rosenberg, P.A., Bergin, A., Peters, J., et al. (2019). Recurrent SLC1A2 variants cause epilepsy via a dominant negative mechanism. Ann Neurol *85*, 921–926. 10.1002/ana.25477.

20. Arribas-González, E., De Juan-Sanz, J., Aragón, C., and López-Corcuera, B. (2015). Molecular Basis of the Dominant Negative Effect of a Glycine Transporter 2 Mutation Associated with Hyperekplexia. Journal of Biological Chemistry *290*, 2150–2165. 10.1074/jbc.M114.587055.

21. Zariñán, T., Perez-Solís, M.A., Maya-Núñez, G., Casas-González, P., Conn, P.M., Dias, J.A., and Ulloa-Aguirre, A. (2010). Dominant negative effects of human follicle-stimulating hormone receptor expression-deficient mutants on wild-type receptor cell surface expression. Rescue of oligomerization-dependent defective receptor expression by using cognate decoys. Molecular and Cellular Endocrinology *321*, 112–122. 10.1016/j.mce.2010.02.027.

22. Omasits, U., Ahrens, C.H., Müller, S., and Wollscheid, B. (2014). Protter: interactive protein feature visualization and integration with experimental proteomic data. Bioinformatics *30*, 884–886. 10.1093/bioinformatics/btt607.

23. An, J.-Y., Lin, K., Zhu, L., Werling, D.M., Dong, S., Brand, H., Wang, H.Z., Zhao, X., Schwartz, G.B., Collins, R.L., et al. (2018). Genome-wide de novo risk score implicates promoter variation in autism spectrum disorder. Science *362*, eaat6576. 10.1126/science.aat6576.

24. Wang, K., Li, M., and Hakonarson, H. (2010). ANNOVAR: functional annotation of genetic variants from high-throughput sequencing data. Nucleic Acids Res *38*, e164. 10.1093/nar/gkq603.

25. Rodrigues, C.H., Pires, D.E., and Ascher, D.B. (2018). DynaMut: predicting the impact of mutations on protein conformation, flexibility and stability. Nucleic Acids Res *46*, W350–W355. 10.1093/nar/gky300.

26. Cao, H., Wang, J., He, L., Qi, Y., and Zhang, J.Z. (2019). DeepDDG: Predicting the Stability Change of Protein Point Mutations Using Neural Networks. J Chem Inf Model *59*, 1508–1514. 10.1021/acs.jcim.8b00697.
